# Mass spectrometry reveals age‐dependent collagen decline in murine atria

**DOI:** 10.1111/nyas.15341

**Published:** 2025-04-28

**Authors:** Nathalie Ringström, Charlotte Edling, Giovanna Nalesso, Javier Barallobre‐Barreiro, Kamalan Jeevaratnam

**Affiliations:** ^1^ School of Veterinary Medicine Faculty of Health and Medical Sciences, University of Surrey Guildford UK; ^2^ James Black Centre King's College London London UK

**Keywords:** atria, cardiac aging, collagen, extracellular matrix, mass spectrometry

## Abstract

The cardiac atrial extracellular matrix (ECM) is central to age‐associated cardiac remodeling and subsequent decline in cardiac functioning. Despite this, the composition of the atrial ECM and how it changes with age is not yet known. This study utilized mass spectrometry to evaluate the composition of murine atria in young (12 weeks) and old (77 weeks) C57BL/6J mice. The tissue was decellularized, ECM and ECM‐associated proteins were extracted with GuHCl, and proteins were deglycosylated to enable identification of glycosylated peptides. Two hundred and thirty‐seven ECM and ECM‐associated proteins were found to be significantly differentially expressed with age. Some proteins (MMP9, S100A9, VWA3A, CTSD, CCL8) were more than threefold increased with age, proteoglycans were modestly decreased, while the overall collagen content was markedly decreased. STRING network mapping of physical associations predicted that both PLOD3 and PDGFA interact with the collagens that decreased with age. The results suggest that the mechanism behind age‐associated atrial stiffness is not due to an increase in collagen content as previously believed, but an increase in cross‐linking, potentially facilitated by PLOD3. Additionally, several of the significant proteins have not previously been associated with cardiac aging and thus are potential drug targets for age‐associated cardiac fibrosis and other age‐associated conditions.

## Graphical abstract

Using mass spectrometry, we analyzed the atrial ECM of young (12 weeks) and old (77 weeks) C57BL/6J mice. Results showed a marked decrease in collagen content with age, alongside increases in specific ECM‐related proteins (e.g., MMP9, S100A9, VWA3A). Network analysis suggests that PLOD3 may contribute to increased collagen cross‐linking rather than collagen accumulation, challenging previous assumptions about atrial stiffness. These findings reveal new potential targets for age‐related cardiac fibrosis.

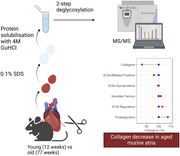

## INTRODUCTION

The cardiac extracellular matrix (ECM) and the balance of its proteomic components are crucial for normal cardiac function. For instance, collagen type I lends strength to the tissue and is essential for the proper functioning of electrical circuits, while elastin is required for the heart to spring back to its original shape after each heartbeat.^1^ As such, changes in the composition of the cardiac ECM can lead to consequences such as increased prevalence of fibrosis, which typically precedes various forms of arrhytmias.^1^ Not surprisingly, the cardiac ECM plays a role in various cardiac pathological conditions, including, but not limited to, myocardial infarction, hypertension, and atrial fibrillation (AF).^2^, ^3^, ^4^ The importance of the cardiac ECM does not only extend to the progression of these and other diseases, but it is also linked to the decline in cardiac functioning associated with aging. Some age‐associated pathological conditions that involve the ECM, such as cardiac fibrosis, remain very difficult to treat, as described previously by Travers et al.^5^

The composition of the cardiac ECM has mainly been studied in the ventricles, especially the left ventricle. At the same time, adverse age‐associated cardiac remodeling of the atria contributes to different types of atrial conduction disorders, most notably AF.^6^, ^7^ In addition to causing fatigue, chest pain, and shortness of breath, AF is associated with an increased risk of stroke and heart failure.^8^, ^9^

The cardiac ECM is rich in post‐translational modifications (PTMs) such as oxidation, phosphorylation, and distinct types of glycosylation. Depending on the PTM and where it occurs in the protein, the modification may change how the protein interacts with its surroundings and it may even alter its structure.^10^, ^11^, ^12^ The oxidation of collagens, hydroxylation, has been proven to change the properties of the molecules in terms of stability and their interaction with other proteins such as integrins.^13^, ^14^ *N*‐glycosylation of, for example, serum and plasma has previously been linked to aging.^15^, ^16^ The relationship between age and *N*‐glycosylation of plasma is so robust that the extent of glycosylation of immunoglobulin G in plasma can be utilized as a biomarker of biological and chronological age.^16^ The relationship between *N*‐glycosylation and aging also extends to the heart.^17^ Thus, it is clear that it is not only the composition of the cardiac ECM in terms of the ratio and abundances of various ECM proteins that plays important roles for normal functioning of the heart, but also the extent and type of PTMs.

Despite the importance of the cardiac ECM for normal cardiac function, little is known about the precise composition of the ECM and how it changes with age. This is largely due to intrinsic difficulties related to studying the cardiac ECM.^1^ Additionally, glycosylation is especially challenging to study with mass spectrometry, due to glycans being very large, diverse, and having poor ionization efficiencies.^18^

The aim of this study was to gain a better understanding of the proteomic composition of murine atria and how it changes with age, with a focus on ECM and ECM‐associated proteins. Tandem mass spectrometry (MS) was employed to evaluate the proteomic composition, including the extent of oxidation and *N*‐glycosylation, of atria from C57BL/6J mice aged 12 and 77 weeks. The tissue was enriched for ECM and ECM‐associated proteins through the process of decellularization with subsequent solubilization and deglycosylation, which has been proven to greatly improve protein coverage of glycoproteins in mass spectrometry studies.^19^ Following liquid chromatography and tandem MS, bioinformatic and statistical analysis was performed.

## METHODS

### Animals

C57BL/6J (Charles River UK Ltd, Janvier Laboratories) male mice aged 12 weeks (10 mice) and 77 weeks (10 mice) were maintained in a controlled environment (23±2°C, 12 h light/dark cycle). After a 3‐day habituation period, the mice were culled by cervical dislocation. The atria were separated from the ventricles, snap‐frozen in liquid nitrogen, and stored at −80°C until further processing. All animal procedures were undertaken by trained personnel in accordance with local legislation, NASPA‐1819‐25. One of the aged samples was removed from further processing due to technical issues isolating the atria.

### ECM protein extraction

A modified three‐step protocol published previously by Barallobre‐Barreiro et al. was used to extract the ECM proteins.^20^ Whole atria were washed five times in a 10× (v/w) wash buffer consisting of cold PBS, 25 mM EDTA, 1/100 (v/v) broad spectrum protease inhibitor cocktail (P8340, Sigma‐Aldrich). Following this, loosely bound and newly synthesized ECM proteins were extracted with a 5× (v/w) buffer consisting of 0.5 M NaCl, 25 mM EDTA, 10 mM Tris HCl pH 7.5, 1/100 (v/v) broad spectrum protease inhibitor cocktail (P8340, Sigma‐Aldrich) through vortexing at low speed for 1 h at room temperature (RT). Samples were subsequently washed with fresh NaCl buffer followed by ddH2O. Next, the tissue was enriched for ECM proteins through decellularization using a 10× (v/w) decellularization buffer consisting of 0.1% SDS, 25 mM EDTA, 1/100 (v/v) broad spectrum protease inhibitor cocktail (P8340, Sigma‐Aldrich), through vortexing at low speed for 16 h at RT, and the tissue was washed with ddH2O to remove residual SDS. Finally, the heavily bound ECM proteins were solubilized through incubation in a 5× (v/w) GuHCl buffer for 72 h at RT through high‐speed vortexing. Silica beads were used to further homogenize the tissue. The samples were centrifuged at 16.000 *g*, 10 min, 4°C. Protein concentration was estimated by the absorbance measurement at 280 nm using the following formula:

$$c\left(\frac{\mathrm{mg}}{\mathrm{mL}}\right)=A280.$$

GuHCl was precipitated from the samples through the incubation with 8× (v/v) ice‐cold EtOH at −20°C ON. The samples were centrifuged 16.000 *g*, 30 min, 4°C. While keeping the samples cold at all times, the ethanol was aspirated, and the pellets were dried using a vacuum centrifuge. The pellets were resuspended in a deglycosylation buffer (1 µL/1 µg protein), consisting of 150 mM NaCl, 50 mM sodium acetate, pH 6.8. 1:200 (v/v) endo‐α‐*N*‐acetylgalactosaminidase, A2‐3, 6, 8, 9‐neuraminidase, and β‐*N*‐acetylglucosaminidase. 1:500 (v/v) endo‐β‐galactosidase and heparinase II. 1:100 (v/v) chondroitinase ABC, 1/100 (v/v) broad spectrum protease inhibitor cocktail (P8340, Sigma‐Aldrich). Samples were incubated at 37°C for 24 h at 180 rpm and then the water was evaporated from the samples and replaced with an equal amount of H_2_^18^O water containing 1:100 PNGase‐F. The samples were then incubated for 48 h at 37°C, 180 rpm.

### Mass spectrometry

#### In‐solution digestion with trypsin

Samples were reduced and alkylated with dithiothreitol and iodoacetamide. Following this, samples were digested in solution with 1:50 (v/v) trypsin, initial incubation 37°C 2 h, then at RT overnight. In solution digestion, liquid chromatography and tandem mass spectrometry and initial data processing was performed by the proteomics facility at King's College London, for a more detailed materials and methods description, see supplementary material to the study by Barallobre‐Barreiro.^21^

#### Liquid chromatography and tandem mass spectrometry

Twenty micrograms from each of the digested peptide samples were separated using an Ultimate 3000 NanoLC system (ThermoFisher Scientific). The peptides were resolved by reverse phase chromatography on a 75 µm * 50 cm C18 column using a three‐step gradient of water in 0.1% formic acid and 80% acetonitrile in 0.1% formic acid over a gradient of 250 nL/min over 120 min. The elute was ionized by electrospray ionization using an Orbitrap Fusion Lumos (ThermoFisher Scientific) operating under Xcalibur v4.4 programmed to acquire data using a Universal CID method by defining a 3 s cycle time among a full MS scan and MS/MS fragmentation. Full‐scan MS spectrum was acquired at a resolution of 120.000 at 200 m/z with 100% normalized AGC target and a scan range of 400–1500 m/z and a maximum injection time of 100 ms. The MS/MS fragmentation was performed CID and quadrupole ion trap analyzer. The following parameters were used: 100% normalized AGC target, normalized collision energy 35, *q*‐value 35, isolation window 1.2 Th, and maximum injection time 50 ms.

#### Data processing

Proteome Discoverer (ThermoScientific v2.5) was used to process the raw mass spectrometry data into a peak list which was matched to Swiss‐Prot's mouse database using Sequest, with two missed cleavages allowed. Carbamidomethylation was included as a fixed modification, and oxidation of proline and lysine, and deamination of asparagine were included as dynamic modifications. Oxidation of proline and lysine were included to accommodate the heavy oxidation typically associated with collagen, and deamination of asparagine in the presence of ^18^O water was included to enable the characterization of *N*‐glycosylation sites. The mass tolerances were set at 10 ppm for the precursor ions, and 0.6 Da for fragment ions. When validating the peptides, a false discovery rate of 0.05 was used. The Minora algorithm in Proteome Discoverer was used to calculate abundances.

#### Statistical analysis

##### Removal of non‐ECM proteins

While decellularization with SDS is efficient in removing cells from cardiac tissue, cells can remain, particularly in murine hearts. Therefore, tissue enriched in ECM proteins through the process of decellularization will contain a degree of contaminating cellular proteins. Thus, these proteins were filtered out prior to subsequent analysis. The MatrisomeDB mouse database, with the addition of a small selection of manually added ECM‐associated proteins, was used to filter for ECM proteins and ECM‐associated proteins.^22^

##### Data preparation

To preprocess the raw data of both the whole protein‐ and peptide data sets, filtering for missing values with cut‐off value set at a minimum of eight samples with detected abundance was performed. The remaining missing values were imputed using the kNN method from the R package DEP. The data set was quantile normalized to account for differences in loading. The log_2_ transformed, filtered, imputed, and normalized abundances were used for all figures.

Sample similarity and principal component analysis (PCA) was performed using R. Outliers were identified as samples with a pairwise correlation average larger than 1.5 times the interquartile range. Two of the samples from the aged group were identified as outliers and, therefore, removed from downstream analysis.

Peptide abundance data is typically less reliable than whole‐protein abundance data in label‐free MS approaches, and any results from this analysis were interpreted with this in mind.

##### Differential expression analysis of whole protein

Differential expression analysis was performed using the R package DEqMS, with the following settings: linear model fitted with empirical Bayesian method and an additional fit using the number of peptides values per protein.

##### Pathway analysis

Interactions between differentially expressed proteins with unadjusted *p*‐value <0.05 were visualized with STRING analysis (default settings). The STRING database consists of data from several different sources including KEGG and Reactome.^23^

#### Post‐translational modification analysis

The abundances of individual peptides, modified and unmodified, were filtered to remove peptides associated with proteins not present in the whole‐protein analysis. The peptide data set was filtered, missing values imputed, and quantile normalized as described above. The differential expression analysis of the modified peptides was done using Limma.

## RESULTS

### Clear divide between young and old

**FIGURE 1:**
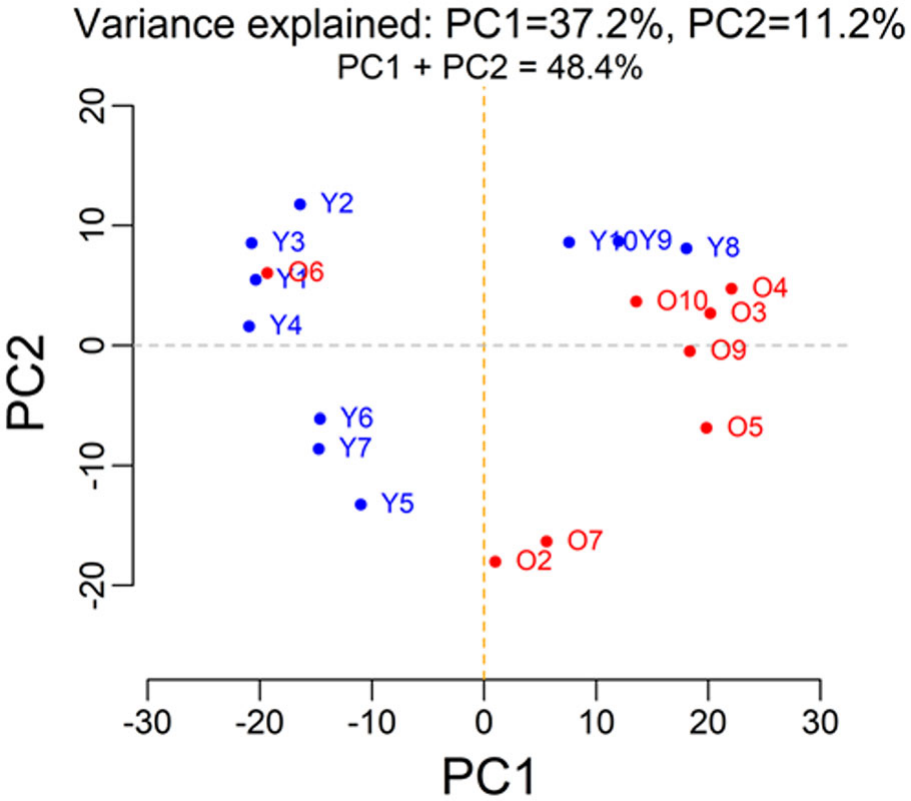
PCA of protein abundances derived from the MS analysis. Principal component 1 (PC1), which explains the largest variance in the data (37%), separates the samples mainly into the age groups. There are three young samples in proximity to the aged samples, and one aged sample in proximity with the young samples; however, none of these were considered outliers by pairwise correlation.

PCA revealed that there is a divide between the two age groups with the exception of a few samples (Figure 1). One of the aged samples was located within the young group on the PCA plot, and three of the young samples formed a group of their own close to the aged group. The PCA of the peptide abundances appeared very similar to the whole‐protein PCA (see Figure S1).

### Aging causes significant changes in the abundance of several ECM‐associated proteins

Differential expression analysis with DEqMS was used to obtain fold changes and its associated statistical significance. The differential expression analysis revealed 237 proteins with *p*‐value <0.05, and 22 proteins with an adjusted *p*‐value <0.05; see Table 1 for significant proteins with a *p*‐value <0.05 and Table S1 for the proteins with an unadjusted *p*‐value <0.05. Twenty‐two collagens were identified as significant (unadjusted *p*‐value; Table 2). All of the collagens, apart from COL8A1, exhibited decreased abundances with age.

**TABLE 1:** Proteins that were significantly differentially expressed with age (adjusted *p*‐value <0.05), their corresponding MatrisomeDB category, and division and log‐fold change (log[old] − log[young]).

Gene	Category	logFC	*p*‐value
*mmp9*	ECM regulators	1.8502	2.38E‐06
*col7a1*	Collagens	−0.360288	4.82E‐05
*s100a9*	Secreted factors	2.06872	0.00029
*col28a1*	Collagens	−0.461349	0.000269
*col5a2*	Collagens	−0.329592	0.000418
*vwa3a*	ECM glycoproteins	1.78598	0.000343
*podnl1*	Proteoglycans	1.14942	0.000143
*emilin2*	ECM glycoproteins	−0.594224	0.000389
*crlf1*	Secreted factors	1.93073	0.000313
*mfap5*	ECM glycoproteins	−1.0183	0.000154
*ccl8*	Secreted factors	3.80268	0.000192
*col6a4*	Collagens	−0.378437	0.00077
*tspear*	ECM glycoproteins	1.09519	0.000722
*adam15*	ECM regulators	1.26778	0.000673
*ctsd*	ECM regulators	2.82345	0.000899
*wnt7a*	Secreted factors	1.06243	0.000784
*inhbb*	Secreted factors	0.86993	0.000971
*pcolce*	ECM glycoproteins	−0.865274	0.000943
*shh*	Secreted factors	0.96633	0.000544
*megf10*	Secreted factors	−0.819676	0.000905
*mepe*	ECM glycoproteins	0.61975	0.000824
*htra1*	ECM regulators	1.00265	0.000952

*Note*: Twenty‐two proteins were identified as being significantly differentially expressed.

**TABLE 2:** Significant (unadjusted *p*‐value <0.05) collagens, corresponding *p*‐values, and log‐fold change (young vs. old).

Gene	logFC	*p*‐value
*col7a1*	−0.36029	4.82E‐05
*col28a1*	−0.46135	0.000269
*col5a2*	−0.32959	0.000418
*col6a4*	−0.37844	0.00077
*col9a2*	−0.36054	0.001772
*col1a2*	−0.27084	0.002036
*col1a1*	−0.2954	0.002104
*col11a2*	−0.6885	0.00256
*col6a5*	−0.49852	0.002869
*col11a1*	−0.2071	0.002978
*col23a1*	−0.38256	0.003304
*col4a2*	−0.3265	0.004386
*col14a1*	−0.41851	0.004683
*col4a4*	−0.22913	0.006751
*col2a1*	−0.33207	0.008515
*col16a1*	−0.36744	0.013305
*col4a1*	−0.23718	0.014265
*col9a1*	−0.38474	0.016597
*col15a1*	−0.28111	0.029822
*col8a1*	0.350151	0.032822
*col12a1*	−0.26782	0.03791
*col5a1*	−0.36851	0.047481

*Note*: All 22 collagens, apart from COL8A1, had decreased abundance in old, with log_2_‐fold changes ranging from −0.6886 to 0.350151.

**FIGURE 2:**
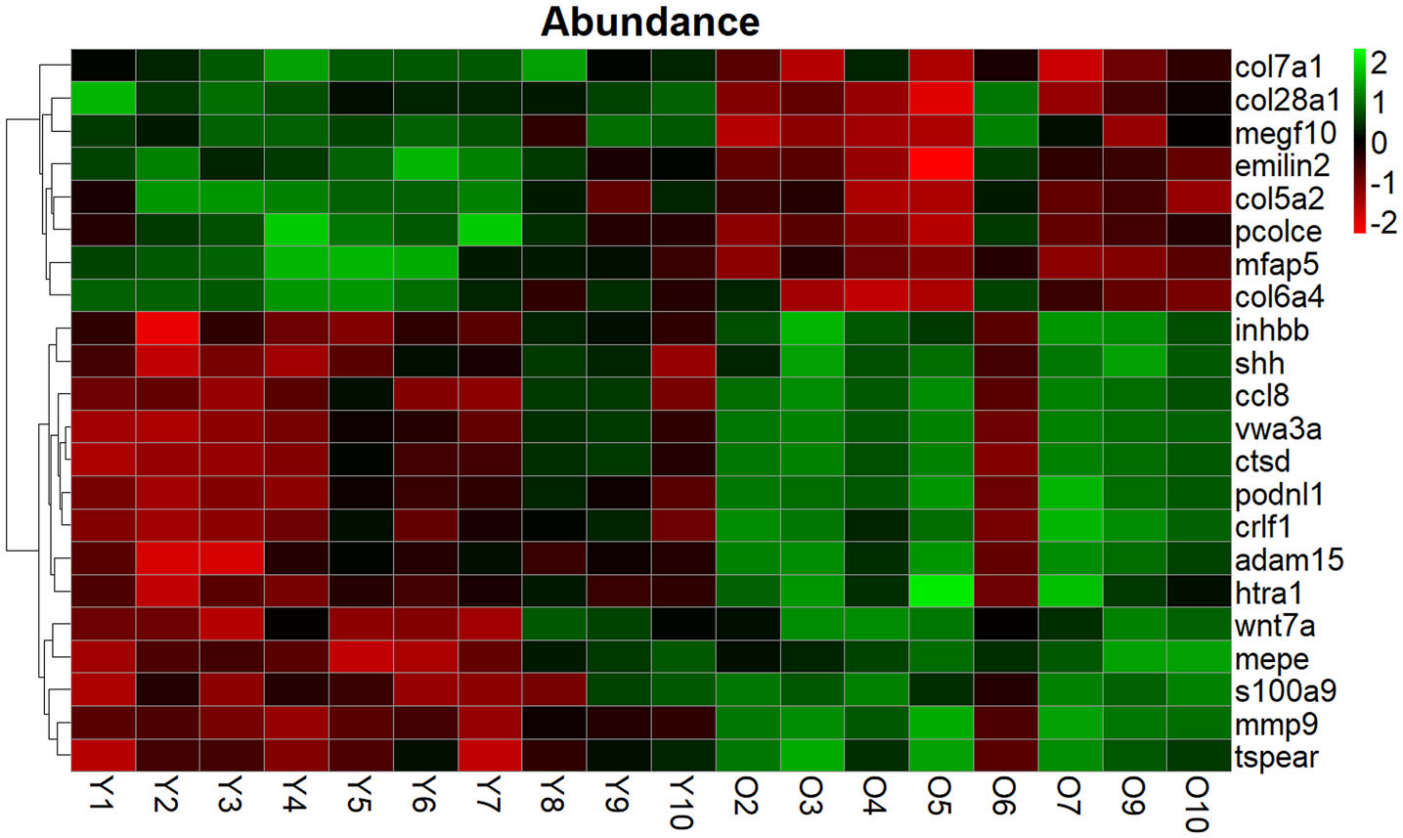
Heatmap showing the whole‐protein abundance of proteins, with Benjamini−Hochberg adjusted *p*‐value <0.05. Green cells correspond to proteins with increased abundance, and red cells correspond to proteins that exhibited decreased abundance with age. The data has been row scaled using the mean and standard deviation, and row clustering was performed based on Euclidean distance. The abundance levels in each age group possessed similar intensities, with the exceptions of three young samples and one old sample.

For the heatmap visualization, the whole‐protein abundances were row scaled based on mean and standard deviation (Figure 2). The heatmap shows the same pattern as the PCA plot in terms of the four samples being dissimilar to their respective age group, with green cells corresponding to proteins with increased abundance and red cells corresponding to proteins with decreased abundance. Sample O6 had a similar abundance of the selected proteins as any of the young samples, while samples Y8 and Y9, and partly O10, had abundance in between the two age groups. As can be seen, several collagens were among the most significantly decreased with age. In the analysis, 980 ECM and ECM‐associated proteins were identified in total (Table 3).

**TABLE 3:** Nine hundred and eighty ECM and ECM‐associated proteins were identified.

Division	Category	Number of detected unique proteins	Percent/category based on raw abundances
Core matrisome	Collagens	38	19.6%
	ECM glycoproteins	172	24.2%
	Proteoglycans	36	4.6%
Matrisome‐associated	ECM‐affiliated proteins	189	17.4%
	ECM regulators	226	17.7%
	Secreted factors	319	16.5%

*Note*: Most proteins were secreted factors (319), followed by ECM regulators (226), ECM‐affiliated proteins (189), ECM glycoproteins (172), collagens (38), and lastly proteoglycans (36). The abundancies of the proteins in each MatrisomeDB category were not correlated to the number of proteins identified, as, for example, collagens was one of the two categories with the lowest number proteins identified, but with a relatively high abundance of 19.6%.

**FIGURE 3:**
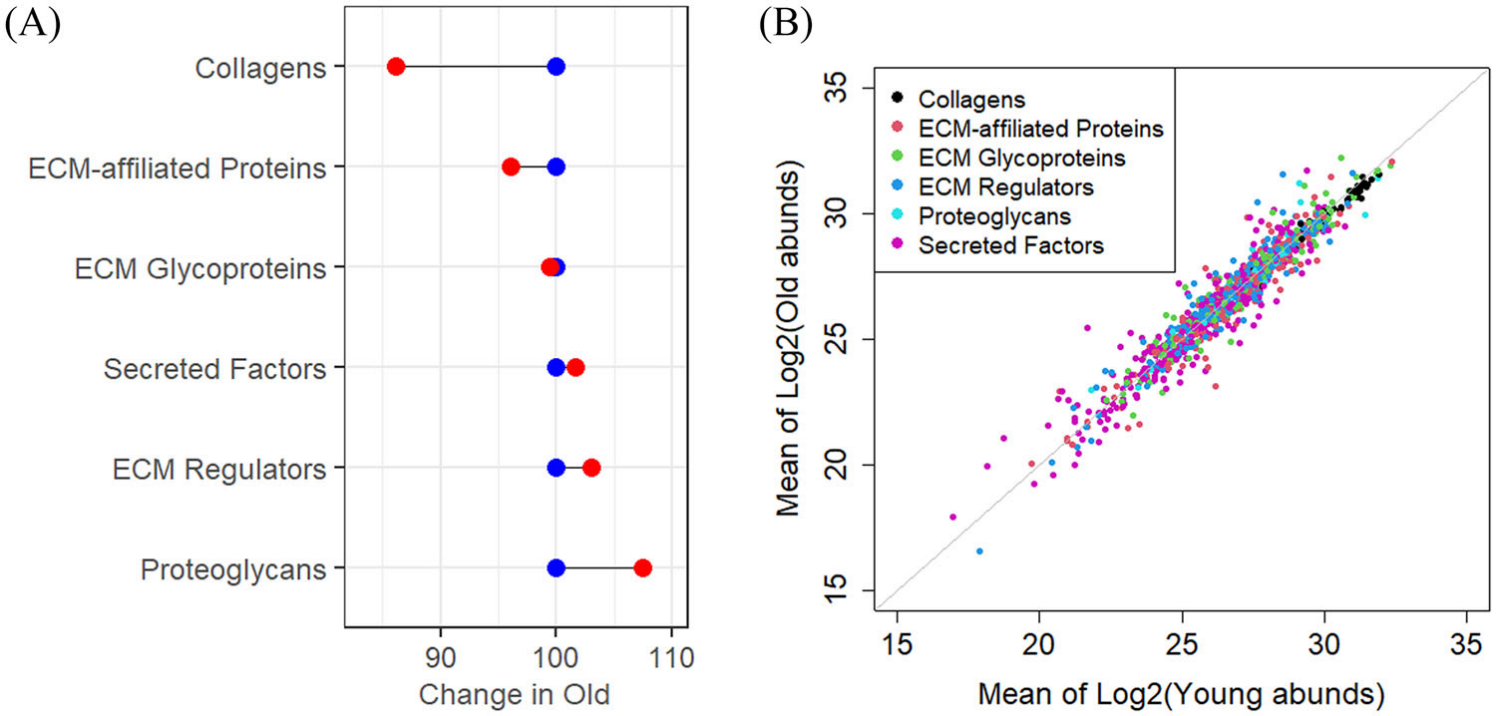
(A) Cleveland plot visualizing changes in protein abundance levels of MatrisomeDB categories with age, blue dots representing young abundance levels and red dots representing aged abundance levels. The mean of log_2_‐fold changes from the differential expression analysis, translated to percentages with young samples set to 100%, were plotted per MatrisomeDB category. As the plot is based solely on the calculated fold change, neither the number of proteins in each group nor the size of the proteins impacts the change in abundance per category. The largest change was seen in collagens that decreased by 15% with age. Proteoglycans were found to be increased with age, while the other categories were mainly unaltered in the two age groups. (B) Mean log_2_ transformed whole protein abundances plotted young against old abundances. Dots have been colored according to the corresponding MatrisomeDB category. In total, glycoproteins were the most abundant proteins. However, as this group contained a high number of proteins, glycoproteins were not the category with the highest mean abundance. The collagen group contained fewer unique proteins, however, larger and more plentiful, therefore, resulting in the highest mean abundance values, in both the aged and young groups, with a higher abundance in young. Glycoproteins was the second most abundant MatrisomeDB category, and the remaining categories exhibited mixed abundance levels.

To evaluate how aging affects the proteomic composition in a broader manner, the proteins were sorted based on their MatrisomeDB category. The proteins in the MatrisomeDB database are divided into two classes: core matrisome and matrisome‐associated. These, in turn, are divided into categories: collagens, proteoglycans, glycoproteins, ECM‐affiliated, ECM regulators, and secreted factors, based on their structure, function, and/or location. The mean of the log_2_‐fold changes of all proteins per category were calculated and visualized with a Cleveland plot (Figure 3A). Interestingly, the data demonstrate that aging markedly reduced the abundance of collagens, with a reduction of almost 15% as a group. This reduction was also seen at a protein‐independent level as indicated in the heatmap (Figure 2). Proteoglycans showed increased abundance in the old, while other ECM categories showed only modest changes at the group level. As a complement to the relative differential expression depicted in Figure 3A, absolute protein abundances in young and old were summarized as a dot plot (Figure 3B). The plot highlights that collagens as a group do not have the largest number of unique proteins; however, due to their high abundance and generally large size, collagens yield more spectra that can be matched to the master protein in mass spectrometry analysis, ultimately resulting in the higher abundance values. In addition, almost all collagens are more abundant in the young versus the old. Glycoproteins were also revealed to be relatively high in abundance, while the other proteins were more evenly distributed, indicating category‐independent abundance levels and less consistent changes with age.

Peptide analysis revealed a slight increase in the abundance of oxidized peptides with age of all MatrisomeDB categories, except for 2× oxidized peptides (see Figure S2). There was also a small decrease in N‐glycosylated peptide abundances with age. Due to the limitations of unlabeled peptide data and the dynamic nature of oxidation modifications and the absence of a control for spontaneous deamination, these results will not be discussed further here, but a more detailed description can be found in the Supplementary Data, with peptide coverage graphs for fibrillar collagens showing the abundance intensities for the different peptides with their PTMs. The coverage percentages for fibrillar collagens ranged from 55% to 77% in the young sample group and 52% to 74% in the old sample group (Figure S3). In addition, the number of uniquely identified peptides in each category, and how many of these were N‐glycosylated are presented in Table S2. A comparison of uniquely identified peptides in young and old samples revealed that of the 16,416 peptides identified in the young samples, 14,709 were overlapping with the 15,574 peptides identified in the old samples (Figure S4).

**FIGURE 4:**
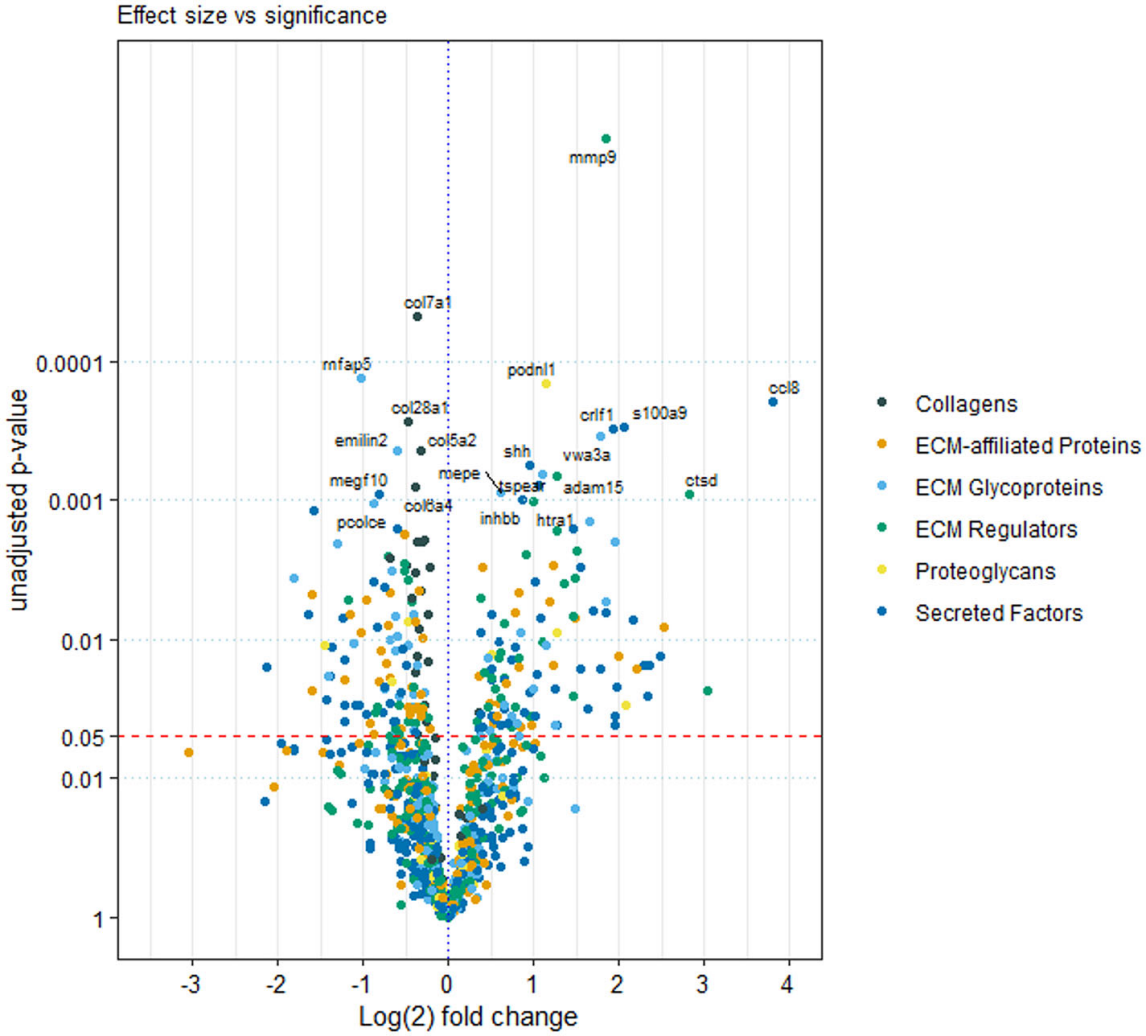
Volcano plot visualizing the relationship between fold change (old − young) and significance. Proteins with adjusted *p*‐value < 0.05 are labeled in the plot. With age, there were more highly significantly upregulated proteins simultaneously exhibiting a large change in abundance as opposed to downregulated proteins, with the most noteworthy example being MMP9.

To demonstrate the relationship between the differential expression effect size and statistical significance, a volcano plot was generated for visualization (see Figure 4). A greater number of significant proteins increased with age, with chemokine ligand 8 (CCL8), cathepsin D (CTSD), S100 calcium‐binding protein A9 (S100A9), cytokine receptor‐like factor 1 (CRLF1), and MMP9 displaying the largest log_2_‐fold changes. Fewer proteins were decreased, and these also displayed a smaller log_2_‐fold change and a lower variability in fold change. The significantly decreased proteins were collagen type VII alpha 1 chain (COL7A1), microfibril‐associated protein 5 (MFAP5), collagen type XXVIII alpha 1 chain (COL28A1), elastin microfibril interfacer 2 (EMILIN2), multiple EGF‐like domains 10 (MEGF10), and procollagen C endopeptidase enhancer (PCOLCE). Proteins were colored based on their corresponding MatrisomeDB category, and demonstrated that similar to Figure 3, the abundance of collagens predominantly decreased in old atria, while the other categories displayed more of a mixed level of effect size.

**FIGURE 5:**
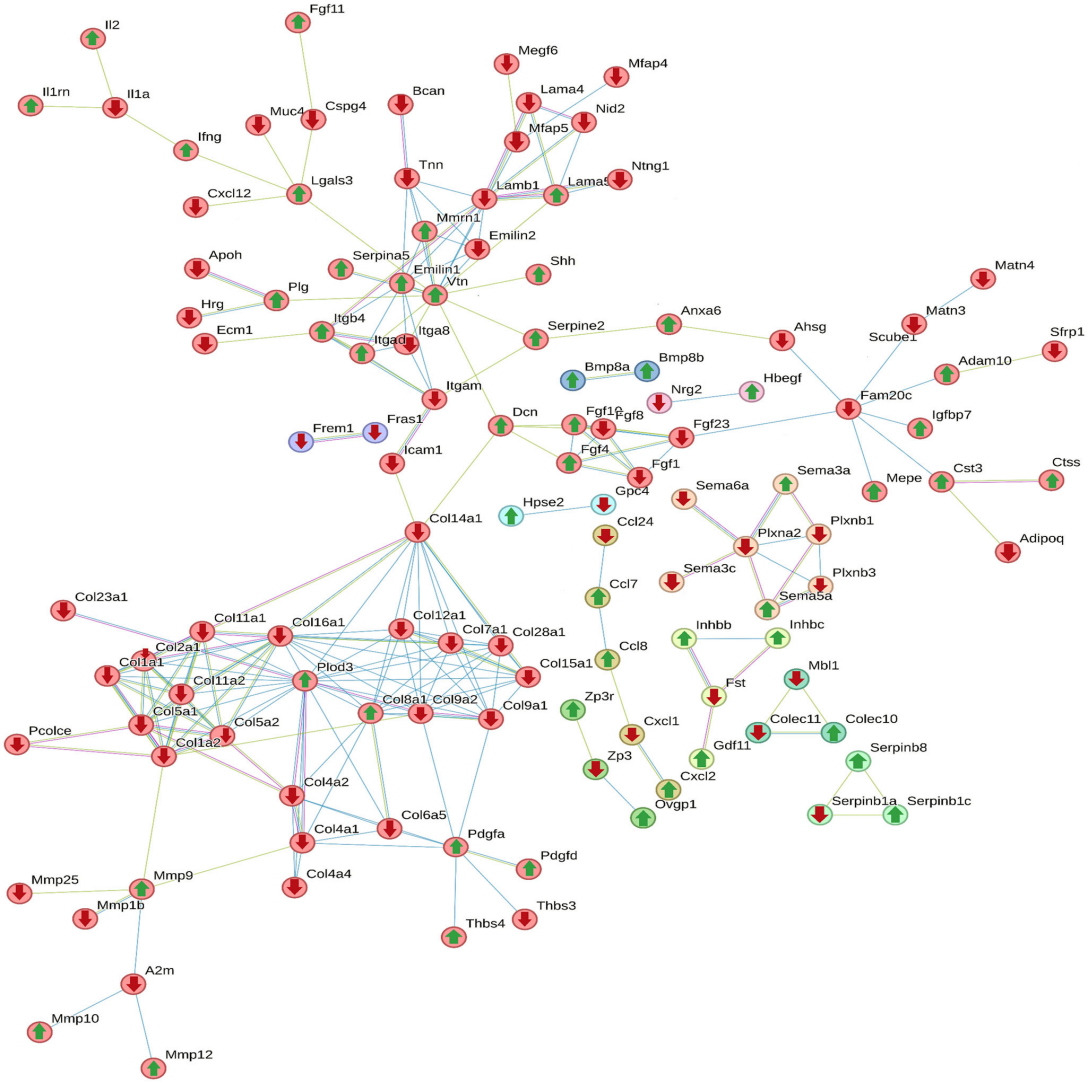
STRING network visualizing predicted physical associations between differentially expressed (unadjusted *p*‐value < 0.05) proteins. K means clustering was employed to generate the clusters. Proteins without any associations have been removed for clarity. The color of the line between nodes signifies the type of evidence, blue corresponding to curated databases such as KEGG, green text mining, and pink corresponds to experimentally validated evidence. Solid lines signify physical interactions between proteins in the same cluster. Proteins with an increased abundance have been marked with an upward facing green arrow, and proteins with decreased abundance have been marked with a downward facing red arrow. PLOD3 had the biggest number of predicted associations and interacted with the surrounding collagens. FAM20C, PLXNA2, PDGFα, FGF4, and MMP9 also had a noteworthy number of predicted physical associations. The cluster containing TNN at the top consisted of proteins with a particularly high degree of physical connections between each other.

STRING pathway analysis of the differentially expressed proteins (*p*‐value <0.05) generated a network map revealing that the collagens with decreased abundance belonged in the same cluster, all interacting with the increased procollagen‐lysine, 2‐oxoglutarate 5‐dioxygenase 3 (PLOD3) in its center (Figure 5). Additionally, the collagens within this cluster had many predicted associations with each other. Family with sequence similarity 20, member C (FAM20C), plexin A2 (PLXNA2), platelet‐derived growth factor α (PDGFα), fibroblast growth factor 4 (FGF4), and MMP9 also had a noteworthy number of predicted physical associations. One of the clusters at the top of the network map contained proteins with a particularly high degree of predicted interactions between the contained proteins; these proteins included, among others, tenascin N (TNN), vitronectin (VTN), laminin subunit beta 1 (LAMB1), and elastin microfibril interfacer 1 (EMILIN1).

## DISCUSSION

As discussed in the introduction, little is known about how aging changes the cardiac ECM. This is especially true for the atria. This study reveals that with aging, the composition of the cardiac atrial ECM, including ECM‐associated proteins, changes considerably. A benefit of evaluating changes in protein composition by evaluating changes as per MatrisomeDB category, as opposed to changes in individual proteins only, is that the data is more statistically powerful, especially concerning mass spectrometry data that is inherently more predictive than conclusive. Interestingly, it was found that the most sizeable change is the decrease of collagens, with the most significant examples being COL7A1, COL28A1, COL5A1, and COL6A4. While mass spectrometry, especially label‐free mass spectrometry, does not produce fully quantitative data due to sample processing and limitations in the peptide searches, the data based on collagen abundances are robust due to the high abundances and number of peptides in this category. Different normalization and missing value assignment methods were trialed (not shown) in this study, and the collagens decreased significantly as a category irrespective of the methods used. Previous studies have reported an increase in collagen overall with age in atria.^24^, ^25^, ^26^ However, these studies were based on other methods such as Masson's trichrome and picrosirius red which produce less quantitative data and consequently have limitations of their own. In conclusion, while our results on the decrease of collagen with age are surprising, they are robust because of the high abundance of collagens in both age groups, the statistical pipeline, and the relative quantitative aspects of mass spectrometry.

As mentioned in the introduction, most studies on how the cardiac ECM changes with age have been conducted on ventricles, especially the left ventricle. While not necessarily relevant to age‐associated changes in the atria, it may still be valuable to compare the results in this study with the results from ventricle‐focused studies. The most substantial difference in the results in this study when compared to those performed on ventricles is the decrease in collagens, which have been found to generally increase in ventricles with age in several different species, with the exception of collagen type III.^24^, ^25^, ^26^, ^27^, ^28^, ^29^, ^30^, ^31^, ^32^, ^33^, ^34^, ^35^, ^36^ However, like the very few studies that have been conducted on how aging affects the collagen in the atria, the majority of these have been conducted utilizing qualitative methods such as Masson's trichrome staining or western blot, rather than mass spectrometry. Fewer studies have explored how aging affects the abundance of ECM proteins other than collagens. Several studies have been conducted that evaluated how aging affects the abundance of fibronectin, laminin, integrin, mimecan, and SPARC. Briefly, the abundance of fibronectin has been shown to increase with age in ventricles from mice but is decreased in hamsters and rats.^28^, ^31^, ^37^ Laminin β1 is increased with age in the left ventricles from mice, but laminin β2 is decreased with age.^30^ Integrin α1 and α5 increase with age in the left ventricles from mice, but integrin β1 decreases.^28^ Mimecan has been shown to decrease with age in whole hearts and left ventricles from mice.^31^, ^38^ Additionally, SPARC has been shown to increase with age in left ventricles from both mice and sheep.^27^, ^34^

In contrast to the age‐associated changes of collagen abundance with age in ventricles and atria, which traditionally are considered to increase with age, the abundance and synthesis of collagen in some other organs such as skin decreases with age.^39^, ^40^ However, in some other organs such as the lungs, there is an increased abundance and synthesis of collagen.^41^ Interestingly, while there are discrepancies in the effects of aging on collagen synthesis and abundance depending on the precise type of tissue, a recent study revealed that with age in *Caenorhabditis elegans*, there was an overall decrease of collagen by ∼20% in samples produced from whole worms.^42^ Through longevity interventions, the expression of COL‐120 was prolonged in the worms. Taken together with the fact that overexpression of COL‐120 in *C. elegans* has already been proven to increase the lifespan of the worms, the study raises the question of whether increased collagen abundance in the heart may have similar effects in humans also, and if these beneficial effects would also extend to the heart, and further emphasizing the need for more studies in this field.^43^

While it is highly relevant to evaluate static abundance values, this study does not address the balance between synthesis and degradation of ECM and ECM‐associated proteins, and it does not address whether the significant changes in abundances stem from an increased expression or a decreased degradation. As MMPs and TIMPs are examples of enzymes highly relevant for the turnover of a number of different ECM proteins, the sharp increase of MMP9 provides a fragment of information about how the relationship between synthesis and degradation may change with age. Similarly, there are many additional proteins involved in this phenomenon, suggesting that there is a plethora of information yet to be discovered.^44^, ^45^, ^46^ It is advisable for future studies to include the NaCl extract with loosely bound and newly synthesized ECM and ECM‐associated proteins to aid understanding of the turnover kinetics, in combination with targeted proteomic methods to evaluate how aging affects the abundances of MMPs and TIMPs especially and methods to evaluate gene expression such as qPCR or RNA sequencing. However, the results from such an experiment including NaCl would have to be interpreted with care, as the NaCl extract would also include markedly higher levels of secreted proteins from the blood circulation, rather than the ECM and ECM‐associated proteins of the interest.

The increasing prevalence of cardiac fibrosis with age would indicate that collagens overall, especially fibrillar collagen, would increase with age. However, there may be other potential causes for age‐associated cardiac fibrosis. With age, the extent of collagen cross‐linking increases in cardiac tissue. Collagen cross‐linking can either occur through the enzymatic action of lysyl oxidase (LOX) and lysyl oxidase‐like proteins (LOXL), or through the reaction between advanced glycation end products and collagens.^47^, ^48^ It can, therefore, be speculated that it is not an increase in collagen that causes stiffening and fibrosis of atria with age, but rather an age‐associated increase of collagen cross‐linking. In the present study, four LOXL proteins were detected together with the LOX protein; none were significantly changed even though LOX showed a trend toward being increased with age, and LOXL1 and LOXL2 were borderline significantly decreased with age. There are, however, many other LOX and LOXL proteins, and it is likely that these were present at greater concentrations in the NaCl extract, which contained loosely bound and newly synthesized ECM proteins and, therefore, not included in the mass spectrometry analysis. Moreover, PLOD3 was significantly increased with age, and the STRING network map predicts that it physically associates with the surrounding collagens. PLOD3 catalyzes the hydroxylation of collagens, thereby enabling LOX and LOXL enzymes to crosslink the collagens.^49^ However, the increased abundance of PLOD3 with age would result in increased hydroxylation of newly synthesized collagens, rather than mature collagens, and in order to fully explore the relationship between PLOD3, hydroxylation, and cross‐linking of collagens, the NaCl extract should also be included in future studies, together with a method that enables evaluation of the cross‐linking of collagens, such as second harmonic generation microscopy. As such, while this study cannot offer conclusive evidence of the causes for age‐associated increase in atrial stiffness, the results and the methods used suggest that the mechanisms behind the increase in stiffness may not be an increase in the overall content of collagen, but rather an increase of collagen cross‐linking. Additionally, it is important to note that an increase in collagen cross‐linking may potentially partly explain the surprising decrease of collagen seen in this study, as this would render the collagens less soluble. While the GuHCl extraction protocol employed in the present study was lengthy, harsh, and has been used successfully in previous studies, it does not fully solubilize the tissue. Full solubilization is possible, for example, with the addition of a homogenization step using a homogenizer followed by sonication, but it would likely lead to missed identifications of ECM and ECM‐associated proteins less abundant than collagens.

The sharp increase of MMP9 with age has previously been reported in the ventricles.^50^ MMP9 and the less significant MMPs (e.g., MMP25 and MMP12) degrade ECM proteins such as collagens. However, the STRING network map only predicts that MMP9 interacts with collagen type IV alpha 1 chain (COL4A1) and collagen type I alpha 1 chain (COL1A2), and none of the other MMPs are predicted to interact with the differentially expressed collagens. However, it is known that MMP9 is capable of associating with and degrading many different types of collagens.^51^, ^52^, ^53^ As such, the sharp increase of MMP9 likely plays an important role in the general decrease of collagens with age in murine atria.

As the stiffness of the atria increases with age, it can be hypothesized that the increase of MMP9, which likely leads to a decrease in collagens in general, is a protective mechanism to prevent the atria from even greater stiffness than what is normally associated with aging. However, other studies suggest that MMP9 is associated with a decline in cardiac function in aging, possibly due to its impact on inflammatory phenomenon.^54^, ^55^ It is possible that MMP9 has both beneficial and detrimental effects on the aged atria: The degradation of collagen may reduce atrial stiffness, while the increase of collagen abundance also leads to an elevated level of inflammation in the tissue.

In addition to MMP9 and PLOD3, the increased abundance of platelet‐derived growth factor subunit A (PDGFA) was predicted to interact with several of the collagens with decreased abundance. PDGFα overexpression is associated with a number of pathological cardiac conditions such as cardiac hypertrophy and fibrosis.^56^ Overexpression of PDGFA leads to an increase of collagen type III alpha 1 (COL3A1) and LOX proteins in an MI rat model, and it also has beneficial effects in terms of ventricular function, partly due to revascularization of the infarcted tissue and an increased ratio of collagen type III to collagen type I.^57^ As collagen type I decreased in this study, and the changes in collagen III were not significant, our study suggests that the increase of PDGFA has protective effects in the aging atria, rather than detrimental effects stemming from increased levels of fibrosis.

While the most noteworthy finding from this study is the decrease of collagens with age in murine atria, as mentioned above, some of the most significantly differentially expressed proteins identified (e.g., MMP9, S100A9, WNT7A, and SHH) do not belong to the MatrisomeDB *core matrisome* division. Many of these proteins are not tightly bound to the ECM and are not typically considered to belong to the ECM. As such, it is likely that the NaCl extract containing loosely bound and newly synthesized ECM proteins would be more representative of the abundances of these proteins in young and old murine atria. It is advisable to consider this fact when studying the fold changes of the matrisome‐affiliated proteins. However, it is likely that the fold change patterns of these proteins would remain the same in the NaCl extracts, and, therefore, our result is a robust prediction of the age‐associated abundance change of these proteins. The composition of the atrial ECM and how it changes with age has not previously been investigated to this extent. The results in this study reveal many novel findings, mainly the overall decrease in collagen content. With this new data, it can be proposed that the mechanisms behind increased stiffness in atria are not due to the increase in collagen content, as commonly believed, but rather an increase in collagen cross‐linking, possibly facilitated by PLOD3. Several new potential key players for age‐related cardiac fibrosis can be identified from the list of differentially expressed proteins, such as COL7A1 and S100A9. In conclusion, the findings from this study aid a better understanding of age‐associated cardiac remodeling and that this information has the potential to be utilized for in vitro models of atrial aging or as drug targets.

## CONCLUSION

In conclusion, several ECM and ECM‐associated proteins exhibit significant changes in abundance with age in the murine atrial ECM, including MMP9, COL7A1, and S100A9. In general, the abundance of collagens drastically decreases with age, and predicted interactions with PLOD3 suggest that an age‐associated increase in cardiac stiffness may be due to an increase in collagen cross‐linking rather than an increase in fibrillar collagens as previously believed.

## AUTHOR CONTRIBUTIONS

K.J. and N.R. conceived the outline of the study, J.B.‐B. invented the protein extraction and deglycosylation protocol used, N.R. and J.B.‐B. performed the experiments, N.R. and C.E. performed the data analysis, N.R. wrote the manuscript, and K.J., C.E., G.N., and J.B.‐B. have edited and contributed to the manuscript.

## COMPETING INTERESTS

The authors declare no competing interests.

## PEER REVIEW

The peer review history for this article is available at https://publons.com/publon/10.1111/nyas.15341.

## Supporting information




**Figure S1**. PCA of peptide abundances derived from the MS analysis. Principal component 1 (PC1), which explains the largest variance in the data (32%), separates the samples mainly into the age groups. As for whole protein data, one of the aged samples (red) was located within the young group (blue) on the PCA plot, and three of the young samples formed a group of their own close to the aged group.
**Figure S2**. Changes in modified and unmodified peptide abundances of all peptides and fibrillar collagens (top panels) and aggregated fold changes (old compared to young) of all peptides and fibrillar collagens (bottom panels). The fibrillar collagens included collagen type I, II, III, V, XI, XXIV, and XXVII chains. For the analysis presented in the top panel, the abundance values were unlogged. There was an overall decrease of *N*‐glycosylation for all Matrisome DB categories with age, and an increase of oxidation of all categories. The fibrillar collagens showed a clear decrease overall in abundance with age and a slight relative increase of multi‐oxidized peptides.
**Figure S3**. Peptide coverage graphs of fibrillar collagens in young (left side) and old (right side). Protein gene symbol and percentage coverage are printed above each graph. The intensity per amino acid (*y*‐axis) is derived from the normalized peptide abundances. Amino acid number is displayed linearly on the *x*‐axis. Colored dots represent peptides with post‐translation modifications (PTMs). Pink dots indicate peptides with one identified oxidation, green dots indicate peptides with two identified oxidations, and turquoise dots indicate peptides with three identified oxidations. Brown dots indicate peptides with one identified *N*‐glycosylation, red dots indicate peptides with two identified *N*‐glycosylations, and blue dots indicate peptides with three identified *N*‐glycosylations. Peptides can have both oxidations and *N*‐glycosylations. To be able to distinguish differences of coverage in the two sample groups, the original unfiltered peptide data set was separated into the young sample group (*N* = 10) and the old sample group (*N* = 8 [two samples were removed for being outliers]). These data sets were then filtered separately with less strict filtering criteria than for the main differential analysis. For this analysis, peptides were considered present if they were detected in at least two out of eight samples in either the young or old sample group, respectively. For the young group, a random 8 of the 10 samples were used for each peptide so that the probability ratio would be the same in both groups. Graphs and coverage figures were produced with PrIntMap‐R (see Weaver, SD, DeRosa, CM, Schultz, SR, Champion, MM. (2023). “PrIntMap‐R: an Online Application for Intraprotein Intensity and Peptide Visualization in Bottom‐Up Proteomics” *Journal of Proteome Research*. 22 (2), 432–441. https://doi.org/10.1021/acs.jproteome.2c00606).
**Figure S4**. Venn diagram of number of identified unique peptides in all proteins (A) and in fibrillar collagens (B) in young, respectively, old groups. As for the coverage calculations, the peptides were considered present if they were detected in at least two out of eight samples in either the young or old sample group, respectively.
**Table S1**. All ECM and ECM‐associated proteins after filtering out low abundance and proteins with excessive NA values. MatrisomeDB was used to sort proteins into the ECM and ECM‐associated categories. In the table, the corresponding fold change and *p*‐value derived from the differential expression analysis performed with the DEqMS R package are presented together with the averaged log transformed abundances and summarized peptide details.
**Table S2**. Number of total unique peptides, per category, found in each sample group and number of peptides (and as percentage in brackets) with at least one identified *N*‐glycosylation. As for the coverage calculations, the peptides were considered present if they were detected in at least two out of eight samples in either the young or old sample group, respectively.
For details on protein level, see the peptide report available in the University of Surrey Open research repository.
