# Supplementary material for: Mass spectrometry reveals age‐dependent collagen decline in murine atria
Source: Ann N Y Acad Sci. 2025 Apr 28;1548(1):206–17. doi: 10.1111/nyas.15341 (PMC12220293; doi:10.1111/nyas.15341)
Supplement: Supplementary file 1 — Figure S1. PCA of peptide abundances derived from the MS analysis. Principal component 1 (PC1), which explains the largest variance in the data (32%), separates the samples mainly into the age groups. As for whole protein data, one of the aged samples (red) was located within the young group (blue) on the PCA plot, and three of the young samples formed a group of their own close to the aged group. Figure S2. Changes in modified and unmodified peptide abundances of all peptides and fibrillar collagens (top panels) and aggregated fold changes (old compared to young) of all peptides and fibrillar collagens (bottom panels). The fibrillar collagens included collagen type I, II, III, V, XI, XXIV, and XXVII chains. For the analysis presented in the top panel, the abundance values were unlogged. There was an overall decrease of N‐glycosylation for all Matrisome DB categories with age, and an increase of oxidation of all categories. The fibrillar collagens showed a clear decrease overall in abundance with age and a slight relative increase of multi‐oxidized peptides. Figure S3. Peptide coverage graphs of fibrillar collagens in young (left side) and old (right side). Protein gene symbol and percentage coverage are printed above each graph. The intensity per amino acid (y‐axis) is derived from the normalized peptide abundances. Amino acid number is displayed linearly on the x‐axis. Colored dots represent peptides with post‐translation modifications (PTMs). Pink dots indicate peptides with one identified oxidation, green dots indicate peptides with two identified oxidations, and turquoise dots indicate peptides with three identified oxidations. Brown dots indicate peptides with one identified N‐glycosylation, red dots indicate peptides with two identified N‐glycosylations, and blue dots indicate peptides with three identified N‐glycosylations. Peptides can have both oxidations and N‐glycosylations. To be able to distinguish differences of coverage in the two sample groups, the [file NYAS-1548-206-s001.docx]

Supplementary material

Mass Spectrometry Reveals Age-Dependent Collagen Decline in Murine Atria

Nathalie Ringström^1^, Charlotte Edling^1^, Giovanna Nalesso^1^, Javier Barallobre-Barreiro^2^, Kamalan Jeevaratnam^1^

1. ^University of Surrey School of Veterinary Medicine^
2. ^The James Black Centre, King’s College London^

Supplementary results

Ageing leads to relative changes of oxidation and N-glycosylation of the cardiac ECM

Mass spectrometry peptide level data is typically less consistent than whole-protein level data, with more missing values. In addition, as the whole-protein level data is based on modified as well as unmodified peptide abundances it can be challenging to interpret the peptide modification data. With age, abundance of oxidised peptides of all MatrisomeDB categories increased overall, except for 2x oxidised peptides, see supplementary figure 2. However, mean fold changes of all three oxidisation types increased with age. N-glycosylated peptide abundances decreased slightly with age, but the fold changes indicates that there was a larger relative decrease of N-glycosylation of all peptides. Abundances of all fibrillar collagen peptides belonging to type I, II, III, V, XI, XXIV and XXVII collagen chains decreased with age, including oxidised and N-glycosylated peptides. However, to properly interpret the changes of the modified collagen peptides, the changes of unmodified collagen peptides must be taken into consideration. As there was a decrease in both abundance and fold change of unmodified collagens, the very slight decrease of multi oxidised collagen peptides fold change indicates that there is a relative increase of oxidisation of fibrillar collagens with age. Interestingly, the fold change of 1x oxidised fibrillar collagens was very similar to the fold change of the unmodified fibrillar collagens, indicating no change or a decrease in relative abundance. The changes of N-glycosylated fibrillar collagen peptides were less obvious with negligible changes of abundances. However, the fold change of 2x N-glycosylated peptides indicates a small relative decrease of N-glycosylation with age of fibrillar collagen peptides. Due to the higher number of missing values and lower expression in the peptide data set compared to the whole protein data set (which is common in PTM mass spectrometry) the analyses are more predictive than conclusive.

Considering the presented decreased abundance of fibrillar collagens with age, we investigated any potential difference in peptide coverage of these proteins. The mapping (Supplementary figure 3.) revealed that the patterns of assigned peptides in young and old samples were overall similar with a small difference in coverage level with old samples having less coverage than young samples in 8 out of 10 fibrillar collagens. The coverage levels, 52-77% were within the expected range for these proteins. To further analyse differences on a peptide level, not accounting for abundances (calculated intensity), we counted and compared the number of unique peptides found, with PTMs or unmodified, in young respectively old samples. When counting a peptide as present if found in at least 2 of 8 samples in either group, we found that about 10% of all unique peptides were found in both sample groups (Supplementary figure 4.). In addition, the number of unique N-glycosylated peptides were very similar in both groups when compared per MatrisomeDB category (Supplementary table 2.). In conclusion the unique peptide data analysis indicated relatively small differences in regard to which unique peptides were found and most of the composition differences in the ECM related to how much of each peptide was present.


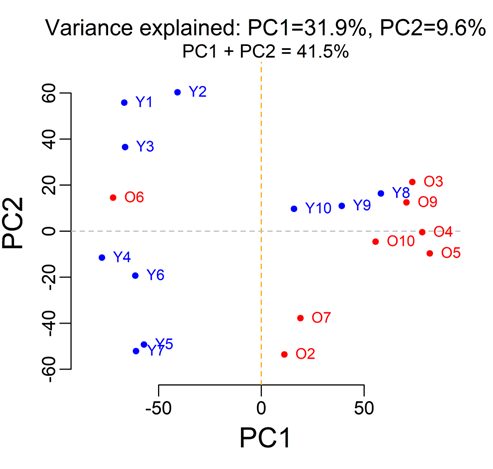


Supplementary figure 1. PCA of peptide abundances derived from the ms analysis. The Principal Component 1 (PC1), which explains the largest variance in the data (32%), separates the samples mainly into the age groups. As for whole protein data one of the aged samples (red) was located within the young group (blue) on the PCA plot, and three of the young samples formed a group of their own close to the aged group.


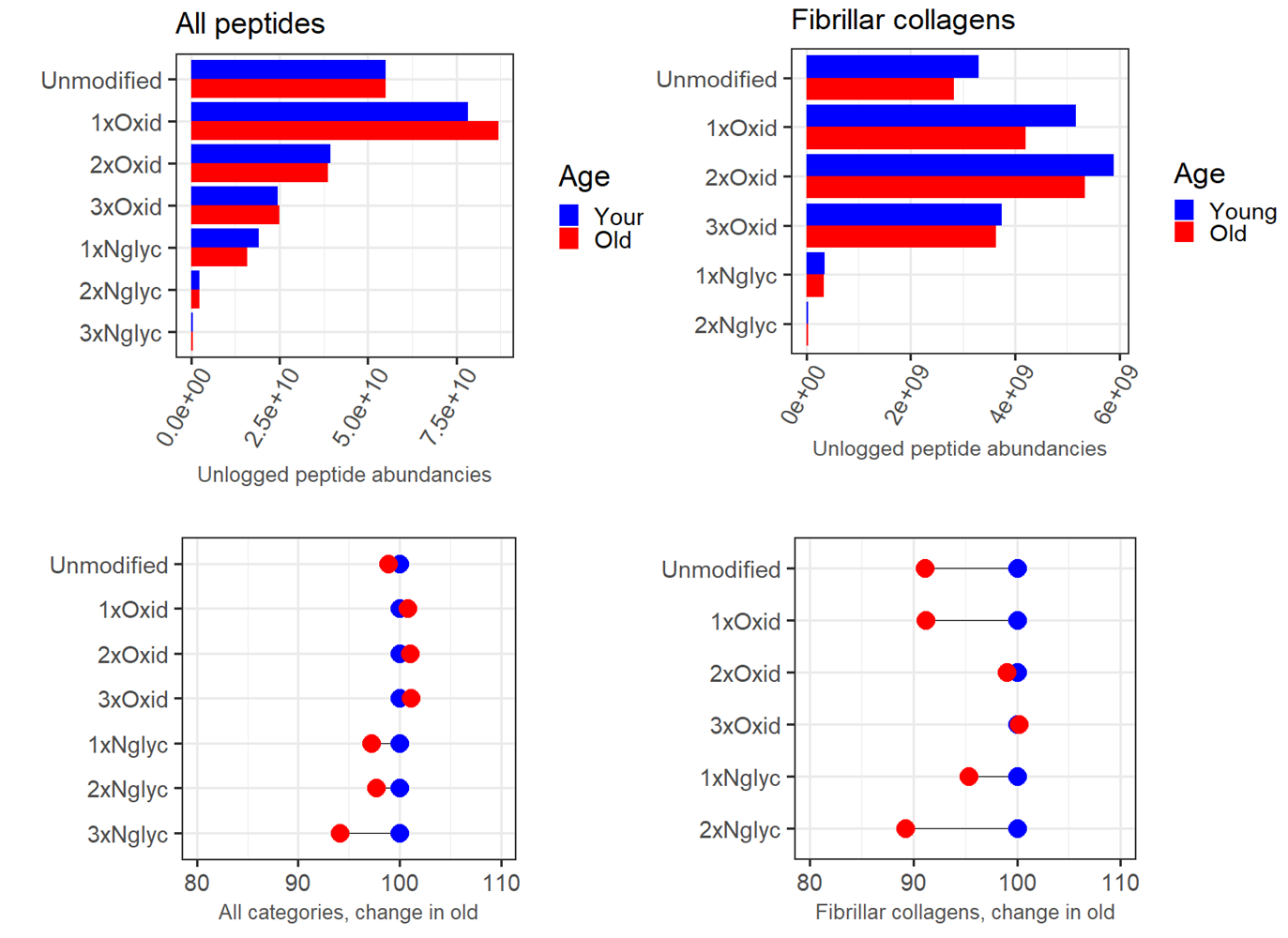


Supplementary figure 2. Changes in modified and unmodified peptide abundances of all peptides and fibrillar collagens (top panels) and aggregated fold changes (old compared to young) of all peptides and fibrillar collagens (bottom panels). The fibrillar collagens included collagen type I, II, III, V, XI, XXIV and XXVII chains. For the analysis presented in the top panel the abundance values were unlogged. There was an overall decrease of N-glycosylation for all Matrisome DB categories with age, and an increase of oxidation of all categories. The fibrillar collagens showed a clear decrease overall in abundance with age and a slight relative increase of multi-oxidised peptides.


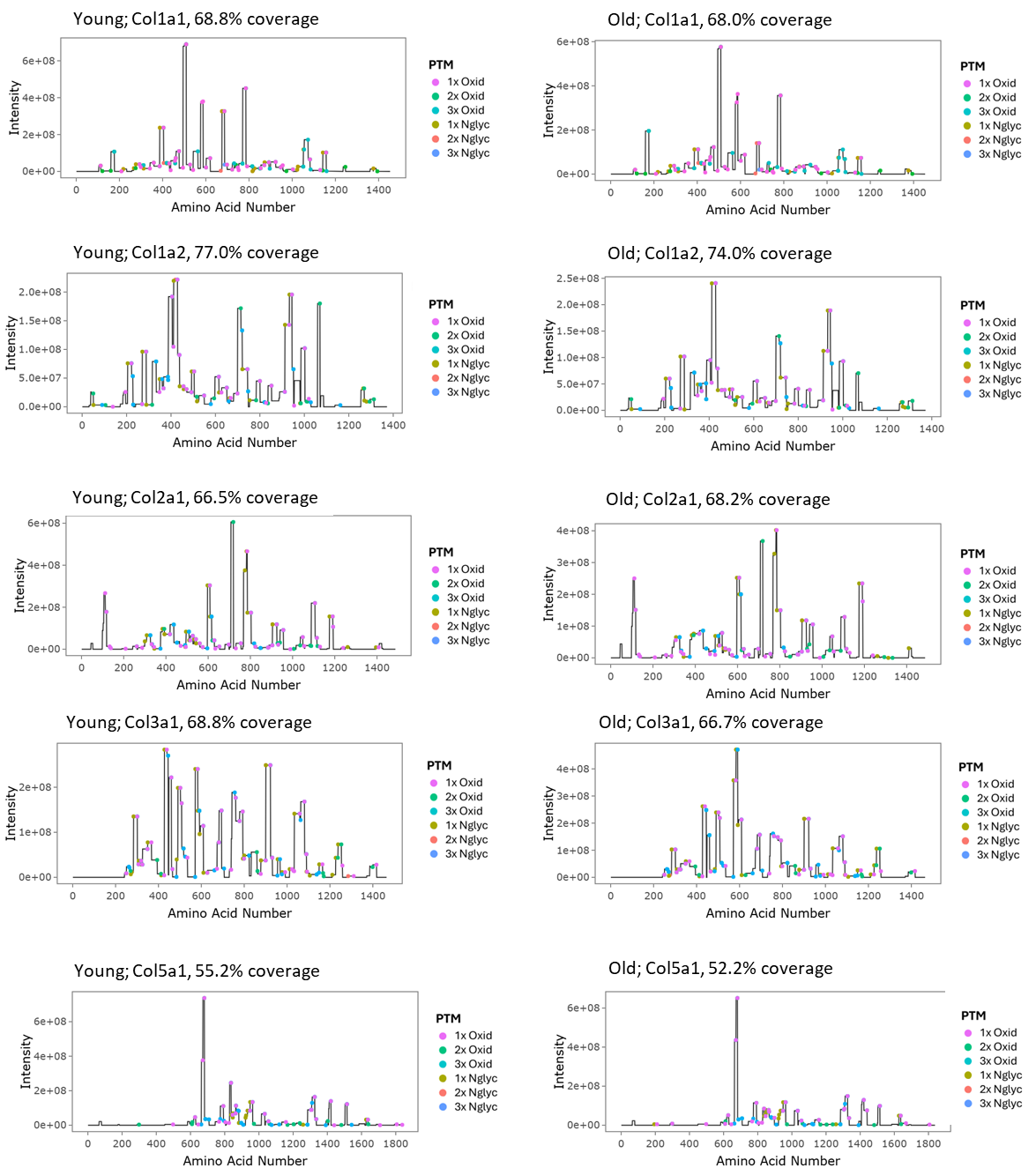


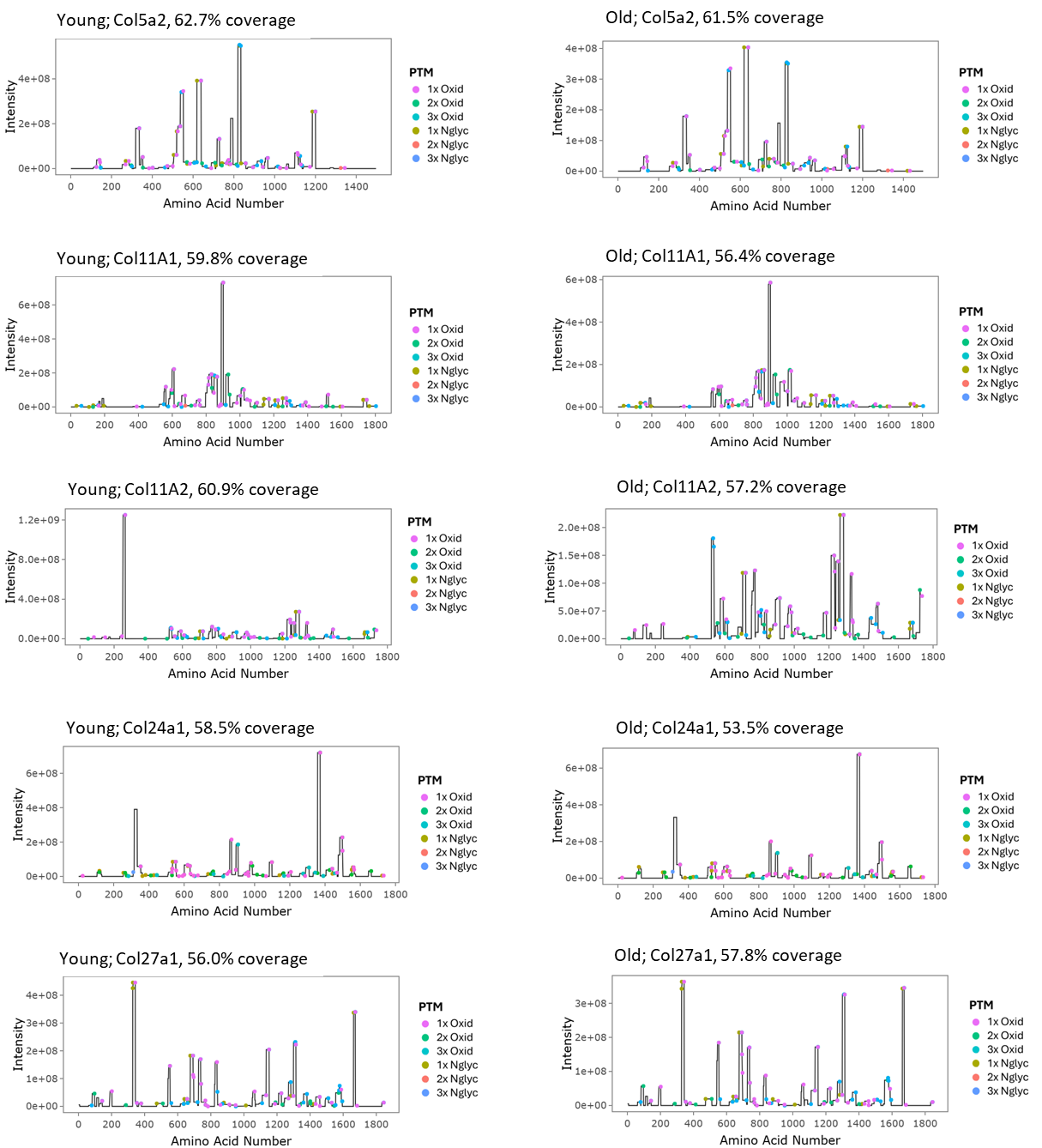


Supplementary fig 3. Peptide coverage graphs of fibrillar collagens in young (left side) and old (right side). Protein gene symbol and percentage coverage is printed above each graph. The intensity per amino acid (Y-axis) is derived from the normalised peptide abundances. Amino acid number is displayed linearly on the X-axis. Coloured dots represent peptides with post translation modifications (PTM). Pink dots indicate peptides with one identified oxidation, green dots indicate peptides with two identified oxidations, turquoise dots indicate peptides with three identified oxidations. Brown dots indicate peptides with one identified N-glycosylation, red dots indicate peptides with two identified N-glycosylations and blue dots indicate peptides with three identified N-glycosylations. Peptides can have both oxidations and N-glycosylations. Peptides were counted as present if found in at least two out of eight samples in the young respectively old group. Graphs and coverage figures were produced with PrIntMap-R (Weaver, SD, DeRosa, CM, Schultz, SR, Champion, MM. **(2023)**. “PrIntMap-R: an Online Application for Intraprotein Intensity and Peptide Visualization in Bottom-Up Proteomics” *Journal of Proteome Research.* 22 (2), 432–441. DOI: 10.1021/acs.jproteome.2c00606).

.


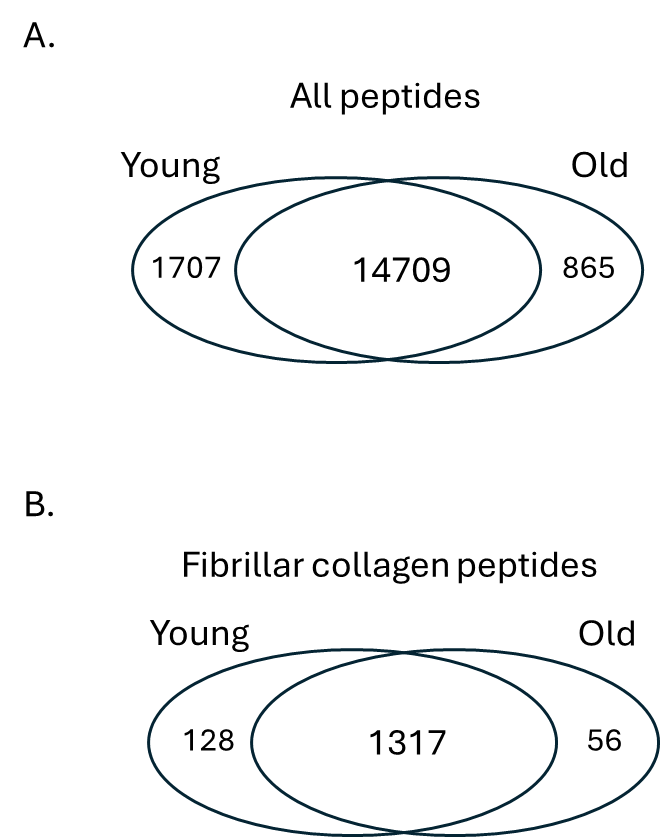


Supplementary fig 4. Venn diagram of number of identified unique peptides in all proteins (A.) and in fibrillar collagens (B.) in young respectively old groups. Peptides were counted as present if found in at least two out of eight samples in the young respectively old group.

Supplementary table 1. All ECM and ECM associated proteins after filtering out low abundance and proteins with excessive NA values. MatrisomeDB was used to sort proteins into the ECM and ECM associated categories. In the table, the corresponding fold change and p-value derived from the differential expression analysis performed with the DEqMS R package are presented together with the averaged log transformed abundances and summarised peptide details.

| **Accession** | **EntrezGeneSymbol** | **Category** | **Division** | **# Peptides** | **PSM** | **# Unique peptides** | **Log2 FC (old compared to young)** | **P-value** | **Adjusted p-value** | **Average abundance** | **Average abundance young** | **Average abundance old** |
| --- | --- | --- | --- | --- | --- | --- | --- | --- | --- | --- | --- | --- |
| **P41245** | mmp9 | ECM Regulators | Matrisome-associated | 21 | 39 | 21 | 1.850205 | 2.38E-06 | 0.002328 | 26.21454 | 25.39222 | 27.24243 |
| **Q63870** | col7a1 | Collagens | Core matrisome | 243 | 903 | 242 | -0.36029 | 4.82E-05 | 0.023598 | 31.75598 | 31.9161 | 31.55582 |
| **Q6P3Y9** | podnl1 | Proteoglycans | Core matrisome | 40 | 69 | 40 | 1.149419 | 0.000143 | 0.037277 | 27.95882 | 27.44797 | 28.59739 |
| **Q9QZJ6** | mfap5 | ECM Glycoproteins | Core matrisome | 5 | 7 | 5 | -1.0183 | 0.000154 | 0.037277 | 24.79497 | 25.24755 | 24.22925 |
| **Q9Z121** | ccl8 | Secreted Factors | Matrisome-associated | 3 | 5 | 3 | 3.802676 | 0.000192 | 0.037277 | 23.35376 | 21.66368 | 25.46636 |
| **Q2UY11** | col28a1 | Collagens | Core matrisome | 102 | 420 | 102 | -0.46135 | 0.000269 | 0.037277 | 30.96828 | 31.17333 | 30.71198 |
| **P31725** | s100a9 | Secreted Factors | Matrisome-associated | 2 | 4 | 2 | 2.068719 | 0.00029 | 0.037277 | 24.11402 | 23.19459 | 25.26331 |
| **Q9JM58** | crlf1 | Secreted Factors | Matrisome-associated | 24 | 86 | 24 | 1.930727 | 0.000313 | 0.037277 | 28.62909 | 27.77099 | 29.70171 |
| **Q3UVV9** | vwa3a | ECM Glycoproteins | Core matrisome | 36 | 84 | 36 | 1.785979 | 0.000343 | 0.037277 | 30.09819 | 29.30442 | 31.0904 |
| **Q8K482** | emilin2 | ECM Glycoproteins | Core matrisome | 43 | 69 | 43 | -0.59422 | 0.000389 | 0.037277 | 26.59873 | 26.86283 | 26.26861 |
| **Q3U962** | col5a2 | Collagens | Core matrisome | 134 | 456 | 133 | -0.32959 | 0.000418 | 0.037277 | 31.09833 | 31.24481 | 30.91522 |
| **Q62226** | shh | Secreted Factors | Matrisome-associated | 18 | 43 | 16 | 0.966327 | 0.000544 | 0.043269 | 26.98273 | 26.55325 | 27.51958 |
| **O88839** | adam15 | ECM Regulators | Matrisome-associated | 21 | 41 | 21 | 1.267775 | 0.000673 | 0.043269 | 26.7301 | 26.16664 | 27.43442 |
| **J3S6Y1** | tspear | ECM Glycoproteins | Core matrisome | 16 | 36 | 16 | 1.095195 | 0.000722 | 0.043269 | 26.81049 | 26.32374 | 27.41893 |
| **A2AX52** | col6a4 | Collagens | Core matrisome | 106 | 316 | 106 | -0.37844 | 0.00077 | 0.043269 | 30.39618 | 30.56437 | 30.18593 |
| **P24383** | wnt7a | Secreted Factors | Matrisome-associated | 18 | 29 | 14 | 1.062431 | 0.000784 | 0.043269 | 26.74941 | 26.27722 | 27.33965 |
| **Q8K4L6** | mepe | ECM Glycoproteins | Core matrisome | 37 | 91 | 37 | 0.619752 | 0.000824 | 0.043269 | 28.08406 | 27.80862 | 28.42837 |
| **P18242** | ctsd | ECM Regulators | Matrisome-associated | 11 | 18 | 11 | 2.823454 | 0.000899 | 0.043269 | 28.87808 | 27.62321 | 30.44667 |
| **Q6DIB5** | megf10 | Secreted Factors | Matrisome-associated | 39 | 91 | 39 | -0.81968 | 0.000905 | 0.043269 | 27.38126 | 27.74556 | 26.92588 |
| **Q61398** | pcolce | ECM Glycoproteins | Core matrisome | 20 | 33 | 20 | -0.86527 | 0.000943 | 0.043269 | 26.69319 | 27.07776 | 26.21248 |
| **Q9R118** | htra1 | ECM Regulators | Matrisome-associated | 21 | 36 | 21 | 1.002646 | 0.000952 | 0.043269 | 25.81254 | 25.36692 | 26.36957 |
| **Q04999** | inhbb | Secreted Factors | Matrisome-associated | 13 | 24 | 13 | 0.869929 | 0.000971 | 0.043269 | 24.5499 | 24.16327 | 25.0332 |
| **P27106** | amh | Secreted Factors | Matrisome-associated | 21 | 37 | 21 | -1.56797 | 0.001208 | 0.051475 | 28.49182 | 29.1887 | 27.62073 |
| **Q61001** | lama5 | ECM Glycoproteins | Core matrisome | 128 | 273 | 128 | 1.66347 | 0.001444 | 0.058983 | 31.31213 | 30.57281 | 32.23628 |
| **Q9JI81** | cts8 | ECM Regulators | Matrisome-associated | 19 | 34 | 19 | 1.264995 | 0.001605 | 0.059565 | 24.17886 | 23.61664 | 24.88164 |
| **P12025** | mdk | Secreted Factors | Matrisome-associated | 3 | 4 | 3 | 1.475106 | 0.001613 | 0.059565 | 24.23714 | 23.58154 | 25.05665 |
| **Q64727** | vcl | ECM-affiliated Proteins | Matrisome-associated | 67 | 143 | 67 | -0.52105 | 0.001641 | 0.059565 | 30.29434 | 30.52592 | 30.00487 |
| **Q07643** | col9a2 | Collagens | Core matrisome | 57 | 158 | 57 | -0.36054 | 0.001772 | 0.062029 | 29.50997 | 29.67021 | 29.30968 |
| **Q8C4U3** | sfrp1 | Secreted Factors | Matrisome-associated | 15 | 24 | 15 | -0.5917 | 0.001935 | 0.06418 | 24.5079 | 24.77088 | 24.17918 |
| **Q9D1H9** | mfap4 | ECM Glycoproteins | Core matrisome | 3 | 4 | 3 | -1.30901 | 0.002005 | 0.06418 | 22.67298 | 23.25476 | 21.94575 |
| **Q01149** | col1a2 | Collagens | Core matrisome | 123 | 683 | 123 | -0.27084 | 0.002036 | 0.06418 | 30.68011 | 30.80049 | 30.52965 |
| **P11087** | col1a1 | Collagens | Core matrisome | 141 | 779 | 139 | -0.2954 | 0.002104 | 0.06418 | 31.5108 | 31.64208 | 31.34669 |
| **Q91XD7** | creld1 | ECM Glycoproteins | Core matrisome | 8 | 85 | 8 | 1.948412 | 0.002316 | 0.06418 | 25.98682 | 25.12086 | 27.06927 |
| **Q8BNJ2** | adamts4 | ECM Regulators | Matrisome-associated | 26 | 64 | 26 | 1.505778 | 0.002329 | 0.06418 | 29.19468 | 28.52545 | 30.03122 |
| **B2RY83** | hpse2 | ECM Regulators | Matrisome-associated | 42 | 99 | 42 | 0.905478 | 0.002403 | 0.06418 | 29.39829 | 28.99586 | 29.90134 |
| **P58022** | loxl2 | ECM Regulators | Matrisome-associated | 24 | 47 | 24 | -0.70865 | 0.002418 | 0.06418 | 26.98294 | 27.2979 | 26.58925 |
| **Q9WVF9** | clec2i | ECM-affiliated Proteins | Matrisome-associated | 6 | 12 | 6 | -1.65142 | 0.00246 | 0.06418 | 22.36381 | 23.09777 | 21.44635 |
| **Q07968** | f13b | ECM Regulators | Matrisome-associated | 23 | 41 | 23 | -0.51594 | 0.002489 | 0.06418 | 24.79092 | 25.02022 | 24.50428 |
| **Q64739** | col11a2 | Collagens | Core matrisome | 152 | 451 | 147 | -0.6885 | 0.00256 | 0.064331 | 30.99206 | 31.29806 | 30.60956 |
| **A6H584** | col6a5 | Collagens | Core matrisome | 123 | 262 | 112 | -0.49852 | 0.002869 | 0.067101 | 30.94337 | 31.16494 | 30.66642 |
| **A6X935** | itih4 | ECM Regulators | Matrisome-associated | 22 | 46 | 22 | -0.5183 | 0.002924 | 0.067101 | 25.95856 | 26.18892 | 25.67062 |
| **Q61245** | col11a1 | Collagens | Core matrisome | 134 | 426 | 124 | -0.2071 | 0.002978 | 0.067101 | 30.96058 | 31.05263 | 30.84553 |
| **Q62217** | sema5a | ECM-affiliated Proteins | Matrisome-associated | 29 | 75 | 29 | 0.411663 | 0.002994 | 0.067101 | 28.46718 | 28.28421 | 28.69588 |
| **P11403** | fgf4 | Secreted Factors | Matrisome-associated | 15 | 28 | 15 | 1.551759 | 0.003068 | 0.067101 | 26.63833 | 25.94866 | 27.50042 |
| **O08665** | sema3a | ECM-affiliated Proteins | Matrisome-associated | 31 | 119 | 31 | 1.228857 | 0.003081 | 0.067101 | 30.78046 | 30.2343 | 31.46316 |
| **Q8K4G2** | col23a1 | Collagens | Core matrisome | 59 | 223 | 59 | -0.38256 | 0.003304 | 0.0704 | 29.61593 | 29.78596 | 29.4034 |
| **Q8R4G0** | ntng1 | ECM Glycoproteins | Core matrisome | 15 | 20 | 15 | -0.66463 | 0.003648 | 0.073633 | 26.72172 | 27.01712 | 26.35248 |
| **Q07235** | serpine2 | ECM Regulators | Matrisome-associated | 12 | 20 | 12 | 1.491956 | 0.003725 | 0.073633 | 25.90474 | 25.24165 | 26.7336 |
| **Q9Z132** | rspo1 | ECM Glycoproteins | Core matrisome | 12 | 16 | 12 | -1.81684 | 0.003732 | 0.073633 | 25.90593 | 26.71341 | 24.89657 |
| **Q9ESB3** | hrg | ECM Regulators | Matrisome-associated | 19 | 53 | 19 | -0.46904 | 0.003757 | 0.073633 | 26.06929 | 26.27775 | 25.80871 |
| **P25085** | il1rn | Secreted Factors | Matrisome-associated | 10 | 14 | 10 | 1.027009 | 0.003857 | 0.074108 | 23.00059 | 22.54414 | 23.57115 |
| **Q8K3F2** | mmp21 | ECM Regulators | Matrisome-associated | 17 | 36 | 17 | 1.351881 | 0.003943 | 0.074316 | 27.40056 | 26.79973 | 28.15161 |
| **P47931** | fst | Secreted Factors | Matrisome-associated | 16 | 50 | 16 | -0.86454 | 0.004107 | 0.075615 | 29.22601 | 29.61025 | 28.74571 |
| **Q9WUD6** | wnt8b | Secreted Factors | Matrisome-associated | 21 | 39 | 21 | -0.74858 | 0.004179 | 0.075615 | 29.78252 | 30.11523 | 29.36665 |
| **P70207** | plxna2 | ECM-affiliated Proteins | Matrisome-associated | 54 | 126 | 48 | -0.33432 | 0.004244 | 0.075615 | 27.75844 | 27.90703 | 27.57271 |
| **P08122** | col4a2 | Collagens | Core matrisome | 125 | 312 | 125 | -0.3265 | 0.004386 | 0.076759 | 30.41448 | 30.55959 | 30.23309 |
| **Q5MJS3** | fam20c | ECM Regulators | Matrisome-associated | 25 | 41 | 25 | -0.42752 | 0.004648 | 0.079121 | 27.58637 | 27.77638 | 27.34886 |
| **Q80X19** | col14a1 | Collagens | Core matrisome | 67 | 172 | 67 | -0.41851 | 0.004683 | 0.079121 | 28.7946 | 28.98061 | 28.5621 |
| **P39039** | mbl1 | ECM-affiliated Proteins | Matrisome-associated | 17 | 54 | 15 | -1.60298 | 0.004845 | 0.07929 | 29.01542 | 29.72786 | 28.12488 |
| **Q9JL99** | clec1b | ECM-affiliated Proteins | Matrisome-associated | 13 | 25 | 13 | 0.835225 | 0.004933 | 0.07929 | 25.38328 | 25.01207 | 25.8473 |
| **Q08189** | tgm3 | ECM Regulators | Matrisome-associated | 32 | 90 | 32 | 0.389058 | 0.004935 | 0.07929 | 27.76251 | 27.5896 | 27.97866 |
| **Q6GQT1** | a2m | ECM Regulators | Matrisome-associated | 46 | 83 | 46 | -1.1744 | 0.005236 | 0.079821 | 28.43964 | 28.96159 | 27.78719 |
| **Q80XD9** | clec2e | ECM-affiliated Proteins | Matrisome-associated | 7 | 15 | 7 | -0.68189 | 0.005241 | 0.079821 | 28.24683 | 28.54989 | 27.868 |
| **Q9QY40** | plxnb3 | ECM-affiliated Proteins | Matrisome-associated | 41 | 121 | 39 | -0.95892 | 0.005293 | 0.079821 | 27.85918 | 28.28536 | 27.32644 |
| **P14824** | anxa6 | ECM-affiliated Proteins | Matrisome-associated | 26 | 64 | 26 | 1.18962 | 0.005294 | 0.079821 | 28.64784 | 28.11912 | 29.30874 |
| **Q60736** | zp3r | ECM Glycoproteins | Core matrisome | 15 | 52 | 15 | 1.847777 | 0.005563 | 0.082609 | 29.01555 | 28.19431 | 30.04209 |
| **P97927** | lama4 | ECM Glycoproteins | Core matrisome | 67 | 142 | 67 | -0.40291 | 0.006319 | 0.08676 | 32.13501 | 32.31409 | 31.91117 |
| **O09164** | sod3 | Secreted Factors | Matrisome-associated | 10 | 19 | 10 | 1.708002 | 0.006368 | 0.08676 | 28.37537 | 27.61626 | 29.32426 |
| **Q80T03** | muc6 | ECM-affiliated Proteins | Matrisome-associated | 37 | 92 | 37 | 0.77783 | 0.006395 | 0.08676 | 28.09322 | 27.74752 | 28.52535 |
| **Q06186** | hbegf | Secreted Factors | Matrisome-associated | 9 | 11 | 9 | 1.848296 | 0.006414 | 0.08676 | 23.64046 | 22.81899 | 24.66729 |
| **P61148** | fgf1 | Secreted Factors | Matrisome-associated | 3 | 4 | 3 | -1.63909 | 0.006501 | 0.08676 | 24.2018 | 24.93029 | 23.2912 |
| **P97737** | gdf10 | Secreted Factors | Matrisome-associated | 22 | 63 | 21 | 0.5101 | 0.006546 | 0.08676 | 28.58405 | 28.35734 | 28.86744 |
| **O55225** | otog | ECM Glycoproteins | Core matrisome | 62 | 96 | 62 | -0.61108 | 0.006575 | 0.08676 | 29.24471 | 29.51631 | 28.90522 |
| **Q9QZR9** | col4a4 | Collagens | Core matrisome | 152 | 517 | 152 | -0.22913 | 0.006751 | 0.08676 | 31.33702 | 31.43886 | 31.20973 |
| **P47993** | xcl1 | Secreted Factors | Matrisome-associated | 3 | 4 | 3 | -1.23171 | 0.006832 | 0.08676 | 20.69139 | 21.23881 | 20.00711 |
| **Q91ZF2** | cts7 | ECM Regulators | Matrisome-associated | 17 | 42 | 17 | 1.4745 | 0.006854 | 0.08676 | 26.82541 | 26.17008 | 27.64458 |
| **Q8VHY0** | cspg4 | ECM-affiliated Proteins | Matrisome-associated | 61 | 109 | 61 | -0.38838 | 0.006875 | 0.08676 | 29.56634 | 29.73895 | 29.35057 |
| **Q5H8B9** | frem3 | ECM-affiliated Proteins | Matrisome-associated | 44 | 75 | 44 | 1.498728 | 0.006956 | 0.08676 | 28.70011 | 28.03401 | 29.53274 |
| **Q9ES30** | c1qtnf3 | ECM-affiliated Proteins | Matrisome-associated | 9 | 31 | 9 | -1.14226 | 0.007041 | 0.08676 | 28.35004 | 28.85772 | 27.71545 |
| **P50592** | tnfsf10 | Secreted Factors | Matrisome-associated | 7 | 32 | 7 | 1.087695 | 0.007111 | 0.08676 | 28.85868 | 28.37526 | 29.46295 |
| **Q8CJ70** | il19 | Secreted Factors | Matrisome-associated | 6 | 10 | 6 | 2.165364 | 0.007171 | 0.08676 | 21.74044 | 20.77806 | 22.94342 |
| **Q61361** | bcan | Proteoglycans | Core matrisome | 29 | 102 | 29 | -0.46461 | 0.007377 | 0.088161 | 31.68806 | 31.89456 | 31.42994 |
| **P20918** | plg | ECM Regulators | Matrisome-associated | 38 | 85 | 38 | 0.652718 | 0.007516 | 0.088744 | 31.25746 | 30.96737 | 31.62008 |
| **Q80V70** | megf6 | Secreted Factors | Matrisome-associated | 33 | 46 | 33 | -0.83171 | 0.007784 | 0.090672 | 26.88061 | 27.25026 | 26.41855 |
| **Q9R069** | bcam | ECM-affiliated Proteins | Matrisome-associated | 18 | 47 | 18 | -0.71189 | 0.007864 | 0.090672 | 26.15339 | 26.46979 | 25.7579 |
| **Q921X9** | pdia5 | ECM-affiliated Proteins | Matrisome-associated | 15 | 53 | 15 | 2.541808 | 0.008118 | 0.092504 | 28.41951 | 27.28982 | 29.83162 |
| **Q61191** | hcfc1 | Secreted Factors | Matrisome-associated | 46 | 90 | 46 | 0.388175 | 0.008243 | 0.092855 | 27.84263 | 27.6701 | 28.05828 |
| **Q8C119** | ndnf | ECM Glycoproteins | Core matrisome | 16 | 27 | 16 | 0.849362 | 0.008381 | 0.093339 | 26.31089 | 25.9334 | 26.78276 |
| **P28481** | col2a1 | Collagens | Core matrisome | 156 | 731 | 153 | -0.33207 | 0.008515 | 0.093764 | 31.35012 | 31.49771 | 31.16564 |
| **Q05895** | thbs3 | ECM Glycoproteins | Core matrisome | 23 | 41 | 23 | -0.59895 | 0.008917 | 0.095256 | 27.31449 | 27.58069 | 26.98174 |
| **P83503** | nyx | Proteoglycans | Core matrisome | 32 | 75 | 32 | 1.26812 | 0.008963 | 0.095256 | 29.73269 | 29.16908 | 30.4372 |
| **Q8JZM8** | muc4 | ECM-affiliated Proteins | Matrisome-associated | 54 | 116 | 54 | -0.30524 | 0.008996 | 0.095256 | 28.60969 | 28.74535 | 28.44011 |
| **P13597** | icam1 | ECM-affiliated Proteins | Matrisome-associated | 18 | 43 | 18 | -1.03088 | 0.00904 | 0.095256 | 27.07687 | 27.53504 | 26.50416 |
| **Q80T14** | fras1 | ECM Glycoproteins | Core matrisome | 79 | 155 | 79 | -0.67614 | 0.010022 | 0.103601 | 29.27986 | 29.58037 | 28.90422 |
| **O35565** | fgf10 | Secreted Factors | Matrisome-associated | 7 | 12 | 7 | 0.588022 | 0.010043 | 0.103601 | 28.56309 | 28.30174 | 28.88977 |
| **Q60841** | reln | ECM Glycoproteins | Core matrisome | 71 | 125 | 71 | -0.46322 | 0.010394 | 0.104272 | 29.45421 | 29.66009 | 29.19687 |
| **O35701** | matn3 | ECM Glycoproteins | Core matrisome | 16 | 26 | 16 | -1.10972 | 0.010442 | 0.104272 | 25.16274 | 25.65595 | 24.54623 |
| **Q80ZN5** | cst13 | ECM Regulators | Matrisome-associated | 7 | 11 | 7 | 1.108811 | 0.01047 | 0.104272 | 22.46554 | 21.97273 | 23.08154 |
| **P16110** | lgals3 | ECM-affiliated Proteins | Matrisome-associated | 9 | 14 | 9 | 1.099305 | 0.010534 | 0.104272 | 23.02896 | 22.54038 | 23.63968 |
| **Q7TQ62** | podn | Proteoglycans | Core matrisome | 35 | 92 | 35 | -1.45612 | 0.010865 | 0.105771 | 30.76152 | 31.40868 | 29.95256 |
| **Q8VCC9** | spon1 | ECM Glycoproteins | Core matrisome | 17 | 27 | 17 | 1.147728 | 0.010901 | 0.105771 | 25.21209 | 24.70199 | 25.84972 |
| **P49003** | bmp5 | Secreted Factors | Matrisome-associated | 20 | 38 | 19 | -1.35698 | 0.011333 | 0.108885 | 28.83032 | 29.43342 | 28.07644 |
| **Q3SXB8** | colec11 | ECM-affiliated Proteins | Matrisome-associated | 11 | 22 | 11 | -0.79976 | 0.012389 | 0.116419 | 26.23818 | 26.59363 | 25.79387 |
| **P70458** | serpina5 | ECM Regulators | Matrisome-associated | 15 | 30 | 15 | 0.592041 | 0.012412 | 0.116419 | 25.14264 | 24.87951 | 25.47156 |
| **Q9R014** | ctsj | ECM Regulators | Matrisome-associated | 13 | 20 | 13 | 0.612277 | 0.012554 | 0.116419 | 27.63055 | 27.35843 | 27.9707 |
| **P01575** | ifnb1 | Secreted Factors | Matrisome-associated | 7 | 32 | 7 | 0.794163 | 0.012797 | 0.116419 | 29.20993 | 28.85697 | 29.65113 |
| **Q9EPC2** | fgf23 | Secreted Factors | Matrisome-associated | 14 | 30 | 14 | -0.542 | 0.012828 | 0.116419 | 26.89065 | 27.13154 | 26.58954 |
| **Q8K4Z0** | lgi2 | ECM Glycoproteins | Core matrisome | 11 | 23 | 11 | -0.62084 | 0.012939 | 0.116419 | 27.4899 | 27.76583 | 27.14499 |
| **P28654** | dcn | Proteoglycans | Core matrisome | 20 | 67 | 20 | 0.504632 | 0.012949 | 0.116419 | 26.78402 | 26.55973 | 27.06437 |
| **Q9Z1W4** | gdf11 | Secreted Factors | Matrisome-associated | 16 | 22 | 14 | 2.485212 | 0.01312 | 0.116712 | 28.46051 | 27.35597 | 29.84119 |
| **Q8BLX7** | col16a1 | Collagens | Core matrisome | 122 | 346 | 122 | -0.36744 | 0.013305 | 0.116712 | 31.28655 | 31.44986 | 31.08241 |
| **Q61171** | prdx2 | ECM-affiliated Proteins | Matrisome-associated | 9 | 32 | 9 | 2.003085 | 0.013339 | 0.116712 | 26.72591 | 25.83565 | 27.83873 |
| **O54824** | il16 | Secreted Factors | Matrisome-associated | 60 | 96 | 60 | 0.748366 | 0.013801 | 0.119694 | 28.98688 | 28.65428 | 29.40264 |
| **P56974** | nrg2 | Secreted Factors | Matrisome-associated | 38 | 75 | 38 | -1.21609 | 0.014031 | 0.119751 | 30.16727 | 30.70776 | 29.49167 |
| **Q5SV42** | serpinb1c | ECM Regulators | Matrisome-associated | 12 | 45 | 9 | 0.83461 | 0.014052 | 0.119751 | 24.97945 | 24.60852 | 25.44313 |
| **P02463** | col4a1 | Collagens | Core matrisome | 129 | 430 | 129 | -0.23718 | 0.014265 | 0.120147 | 30.73707 | 30.84248 | 30.6053 |
| **Q9JLL0** | crim1 | ECM Glycoproteins | Core matrisome | 33 | 61 | 33 | -0.36541 | 0.014411 | 0.120147 | 25.97635 | 26.13876 | 25.77335 |
| **Q8BH34** | sema3d | ECM-affiliated Proteins | Matrisome-associated | 33 | 70 | 32 | -0.71818 | 0.01464 | 0.120147 | 27.31763 | 27.63682 | 26.91864 |
| **O08538** | angpt1 | Secreted Factors | Matrisome-associated | 27 | 50 | 26 | -0.50037 | 0.014649 | 0.120147 | 27.71177 | 27.93416 | 27.43379 |
| **Q8BGZ8** | zpld1 | ECM Glycoproteins | Core matrisome | 16 | 56 | 16 | 0.458625 | 0.014743 | 0.120147 | 25.44583 | 25.242 | 25.70063 |
| **Q9R0E1** | plod3 | ECM Regulators | Matrisome-associated | 25 | 33 | 24 | 0.427738 | 0.014834 | 0.120147 | 27.46629 | 27.27618 | 27.70392 |
| **P20033** | pdgfa | Secreted Factors | Matrisome-associated | 8 | 18 | 8 | 2.352236 | 0.015239 | 0.120961 | 25.93182 | 24.88638 | 27.23861 |
| **Q9CQW5** | lgals2 | ECM-affiliated Proteins | Matrisome-associated | 8 | 17 | 8 | 1.243228 | 0.015243 | 0.120961 | 26.70434 | 26.15179 | 27.39502 |
| **Q03366** | ccl7 | Secreted Factors | Matrisome-associated | 1 | 2 | 1 | 2.28938 | 0.015305 | 0.120961 | 19.7595 | 18.742 | 21.03138 |
| **P01582** | il1a | Secreted Factors | Matrisome-associated | 7 | 8 | 7 | -2.12518 | 0.015523 | 0.121697 | 24.88874 | 25.83326 | 23.70809 |
| **Q8BUE7** | muc20 | ECM-affiliated Proteins | Matrisome-associated | 5 | 11 | 5 | 0.835542 | 0.016025 | 0.124637 | 24.21535 | 23.844 | 24.67954 |
| **Q8K4C5** | il17c | Secreted Factors | Matrisome-associated | 9 | 16 | 9 | 1.562325 | 0.016196 | 0.124887 | 21.69052 | 20.99615 | 22.55847 |
| **Q8BNX1** | clec4g | ECM-affiliated Proteins | Matrisome-associated | 10 | 10 | 10 | 2.214681 | 0.016476 | 0.124887 | 21.69865 | 20.71435 | 22.92903 |
| **P55105** | bmp8b | Secreted Factors | Matrisome-associated | 21 | 56 | 16 | 0.51511 | 0.016546 | 0.124887 | 27.62601 | 27.39708 | 27.91219 |
| **Q05722** | col9a1 | Collagens | Core matrisome | 70 | 180 | 70 | -0.38474 | 0.016597 | 0.124887 | 28.49023 | 28.66122 | 28.27649 |
| **P10889** | cxcl2 | Secreted Factors | Matrisome-associated | 4 | 8 | 3 | 1.784537 | 0.016694 | 0.124887 | 18.93467 | 18.14154 | 19.92608 |
| **P56565** | s100a1 | Secreted Factors | Matrisome-associated | 2 | 2 | 2 | 1.038366 | 0.01749 | 0.12985 | 21.76469 | 21.30319 | 22.34156 |
| **Q0Q236** | bsph2 | ECM Glycoproteins | Core matrisome | 3 | 3 | 3 | -1.40851 | 0.018259 | 0.133666 | 23.67876 | 24.30477 | 22.89626 |
| **P28825** | mep1a | ECM Regulators | Matrisome-associated | 27 | 51 | 27 | 0.6589 | 0.018431 | 0.133666 | 25.94507 | 25.65223 | 26.31113 |
| **P37237** | fgf8 | Secreted Factors | Matrisome-associated | 13 | 29 | 12 | -1.38662 | 0.018454 | 0.133666 | 24.73521 | 25.35148 | 23.96487 |
| **P51655** | gpc4 | ECM-affiliated Proteins | Matrisome-associated | 22 | 45 | 20 | -0.68024 | 0.01855 | 0.133666 | 25.87866 | 26.18099 | 25.50075 |
| **Q02105** | c1qc | ECM-affiliated Proteins | Matrisome-associated | 22 | 89 | 22 | 0.368604 | 0.019266 | 0.137415 | 28.45648 | 28.29265 | 28.66126 |
| **A2ARA8** | itga8 | ECM-affiliated Proteins | Matrisome-associated | 38 | 117 | 38 | -1.21059 | 0.01935 | 0.137415 | 28.62522 | 29.16326 | 27.95267 |
| **P43021** | nodal | Secreted Factors | Matrisome-associated | 11 | 27 | 11 | 0.652578 | 0.019811 | 0.138403 | 27.39249 | 27.10245 | 27.75503 |
| **Q80XH2** | impg2 | Proteoglycans | Core matrisome | 35 | 56 | 35 | -0.67045 | 0.019818 | 0.138403 | 27.62482 | 27.92279 | 27.25234 |
| **Q3V0T4** | itgad | ECM-affiliated Proteins | Matrisome-associated | 31 | 51 | 25 | 0.67513 | 0.019913 | 0.138403 | 27.74796 | 27.4479 | 28.12303 |
| **Q6HA09** | astl | ECM Regulators | Matrisome-associated | 14 | 27 | 14 | 0.515118 | 0.020146 | 0.139038 | 25.38799 | 25.15905 | 25.67417 |
| **Q9R0X2** | adamdec1 | ECM Regulators | Matrisome-associated | 22 | 32 | 22 | 0.541957 | 0.02117 | 0.145082 | 26.27197 | 26.0311 | 26.57305 |
| **P32972** | tnfsf8 | Secreted Factors | Matrisome-associated | 9 | 18 | 9 | 0.513861 | 0.02184 | 0.14863 | 25.59252 | 25.36414 | 25.878 |
| **P97401** | frzb | Secreted Factors | Matrisome-associated | 6 | 17 | 6 | 1.987631 | 0.022239 | 0.14863 | 21.53615 | 20.65276 | 22.64039 |
| **P0C7M9** | clec2l | ECM-affiliated Proteins | Matrisome-associated | 11 | 22 | 11 | -0.8204 | 0.022389 | 0.14863 | 25.10631 | 25.47094 | 24.65054 |
| **Q7TNI7** | il17f | Secreted Factors | Matrisome-associated | 12 | 48 | 12 | 1.255653 | 0.022679 | 0.14863 | 28.08177 | 27.52371 | 28.77936 |
| **Q60571** | crhbp | Secreted Factors | Matrisome-associated | 9 | 12 | 9 | -0.56357 | 0.022792 | 0.14863 | 24.81197 | 25.06245 | 24.49888 |
| **Q9Z2L7** | crlf3 | Secreted Factors | Matrisome-associated | 13 | 17 | 13 | -0.75218 | 0.022815 | 0.14863 | 27.54669 | 27.88099 | 27.12881 |
| **Q9Z1T2** | thbs4 | ECM Glycoproteins | Core matrisome | 33 | 58 | 33 | 1.007467 | 0.022816 | 0.14863 | 29.07293 | 28.62516 | 29.63263 |
| **Q9DCQ7** | serpina1f | ECM Regulators | Matrisome-associated | 17 | 23 | 17 | -0.39714 | 0.022946 | 0.14863 | 30.64109 | 30.8176 | 30.42046 |
| **Q8BHE5** | bmp3 | Secreted Factors | Matrisome-associated | 15 | 16 | 15 | 0.951105 | 0.023095 | 0.14863 | 24.58924 | 24.16653 | 25.11764 |
| **Q91VD1** | lgals12 | ECM-affiliated Proteins | Matrisome-associated | 16 | 26 | 16 | -0.41751 | 0.023321 | 0.14863 | 25.54189 | 25.72745 | 25.30994 |
| **P34960** | mmp12 | ECM Regulators | Matrisome-associated | 9 | 74 | 9 | 3.032008 | 0.023356 | 0.14863 | 29.88606 | 28.5385 | 31.57051 |
| **Q60994** | adipoq | ECM Glycoproteins | Core matrisome | 11 | 31 | 11 | -0.74323 | 0.023988 | 0.14928 | 26.90547 | 27.2358 | 26.49256 |
| **P35230** | reg3b | ECM-affiliated Proteins | Matrisome-associated | 4 | 15 | 4 | -1.60628 | 0.024056 | 0.14928 | 25.11332 | 25.82722 | 24.22095 |
| **Q80Z71** | tnn | ECM Glycoproteins | Core matrisome | 48 | 102 | 48 | -0.40191 | 0.024114 | 0.14928 | 28.49929 | 28.67792 | 28.27601 |
| **O88322** | nid2 | ECM Glycoproteins | Core matrisome | 25 | 90 | 25 | -0.28232 | 0.024175 | 0.14928 | 30.83891 | 30.96439 | 30.68207 |
| **Q8K4Q8** | colec12 | ECM-affiliated Proteins | Matrisome-associated | 44 | 133 | 44 | -0.32252 | 0.02422 | 0.14928 | 28.37613 | 28.51947 | 28.19695 |
| **P21460** | cst3 | ECM Regulators | Matrisome-associated | 11 | 16 | 11 | 1.470382 | 0.024982 | 0.153018 | 22.92886 | 22.27536 | 23.74574 |
| **P02469** | lamb1 | ECM Glycoproteins | Core matrisome | 48 | 88 | 47 | -0.56845 | 0.025224 | 0.153189 | 28.54217 | 28.79481 | 28.22637 |
| **O35598** | adam10 | ECM Regulators | Matrisome-associated | 31 | 45 | 31 | 0.605293 | 0.025323 | 0.153189 | 28.50435 | 28.23534 | 28.84063 |
| **Q6NZL8** | scube1 | Secreted Factors | Matrisome-associated | 23 | 43 | 20 | 2.342114 | 0.025676 | 0.154369 | 30.41896 | 29.37802 | 31.72013 |
| **Q9JKC0** | ccl24 | Secreted Factors | Matrisome-associated | 4 | 7 | 4 | -1.42892 | 0.026888 | 0.160674 | 23.81304 | 24.44812 | 23.0192 |
| **O89029** | matn4 | ECM Glycoproteins | Core matrisome | 24 | 45 | 24 | -0.68135 | 0.028011 | 0.164555 | 28.50677 | 28.80959 | 28.12824 |
| **P43137** | reg1 | ECM-affiliated Proteins | Matrisome-associated | 11 | 38 | 11 | 0.488615 | 0.028738 | 0.164555 | 26.02215 | 25.80499 | 26.2936 |
| **A2A863** | itgb4 | ECM-affiliated Proteins | Matrisome-associated | 69 | 150 | 69 | 0.594997 | 0.029065 | 0.164555 | 29.61062 | 29.34617 | 29.94117 |
| **Q6PZE0** | muc19 | ECM-affiliated Proteins | Matrisome-associated | 45 | 92 | 45 | -0.35891 | 0.029327 | 0.164555 | 28.58573 | 28.74525 | 28.38633 |
| **Q8VHD8** | hrnr | Secreted Factors | Matrisome-associated | 22 | 47 | 22 | -0.67401 | 0.029449 | 0.164555 | 29.17012 | 29.46968 | 28.79567 |
| **Q3U435** | mmp25 | ECM Regulators | Matrisome-associated | 26 | 53 | 26 | -0.57884 | 0.029455 | 0.164555 | 27.1052 | 27.36246 | 26.78362 |
| **Q9ER58** | spock2 | Proteoglycans | Core matrisome | 11 | 69 | 11 | 2.089858 | 0.029478 | 0.164555 | 30.05966 | 29.13083 | 31.22069 |
| **P40224** | cxcl12 | Secreted Factors | Matrisome-associated | 6 | 13 | 6 | -1.20612 | 0.02954 | 0.164555 | 24.95011 | 25.48616 | 24.28004 |
| **Q61508** | ecm1 | ECM Glycoproteins | Core matrisome | 34 | 71 | 34 | -0.43421 | 0.029634 | 0.164555 | 27.71501 | 27.90799 | 27.47378 |
| **Q61581** | igfbp7 | ECM Glycoproteins | Core matrisome | 12 | 21 | 12 | 0.665729 | 0.029774 | 0.164555 | 26.94777 | 26.65189 | 27.31762 |
| **P29699** | ahsg | Secreted Factors | Matrisome-associated | 12 | 20 | 12 | -1.08155 | 0.029791 | 0.164555 | 26.1292 | 26.60989 | 25.52834 |
| **O35206** | col15a1 | Collagens | Core matrisome | 41 | 137 | 41 | -0.28111 | 0.029822 | 0.164555 | 29.79098 | 29.91591 | 29.63481 |
| **Q684R7** | frem1 | ECM-affiliated Proteins | Matrisome-associated | 62 | 127 | 62 | -0.47642 | 0.029827 | 0.164555 | 28.99978 | 29.21152 | 28.7351 |
| **P97873** | loxl1 | ECM Regulators | Matrisome-associated | 28 | 51 | 28 | -0.29308 | 0.029889 | 0.164555 | 28.34845 | 28.47871 | 28.18563 |
| **Q9CXI5** | manf | Secreted Factors | Matrisome-associated | 10 | 14 | 10 | -1.03651 | 0.030092 | 0.164749 | 27.22861 | 27.68928 | 26.65277 |
| **Q62181** | sema3c | ECM-affiliated Proteins | Matrisome-associated | 34 | 63 | 34 | -0.38194 | 0.030455 | 0.165432 | 27.90415 | 28.0739 | 27.69196 |
| **Q62179** | sema4b | ECM-affiliated Proteins | Matrisome-associated | 27 | 62 | 27 | -0.30466 | 0.030576 | 0.165432 | 27.99212 | 28.12752 | 27.82286 |
| **Q9R001** | adamts5 | ECM Regulators | Matrisome-associated | 36 | 57 | 36 | 0.46181 | 0.030851 | 0.165432 | 25.25325 | 25.048 | 25.50981 |
| **O08800** | serpinb8 | ECM Regulators | Matrisome-associated | 11 | 13 | 11 | 0.653319 | 0.030892 | 0.165432 | 27.07071 | 26.78035 | 27.43367 |
| **P55104** | inhbc | Secreted Factors | Matrisome-associated | 12 | 14 | 12 | 1.641675 | 0.03118 | 0.166068 | 25.91404 | 25.18441 | 26.82608 |
| **Q8CEK3** | spinkl | ECM Regulators | Matrisome-associated | 4 | 8 | 4 | -0.83023 | 0.031526 | 0.166137 | 21.43316 | 21.80215 | 20.97192 |
| **O55123** | mmp10 | ECM Regulators | Matrisome-associated | 11 | 32 | 4 | 0.781189 | 0.031532 | 0.166137 | 28.13765 | 27.79046 | 28.57165 |
| **P04351** | il2 | Secreted Factors | Matrisome-associated | 3 | 8 | 1 | 0.65449 | 0.032157 | 0.168002 | 21.51017 | 21.21928 | 21.87377 |
| **Q811B3** | adamts12 | ECM Regulators | Matrisome-associated | 63 | 112 | 63 | -0.22954 | 0.032377 | 0.168002 | 29.99464 | 30.09666 | 29.86712 |
| **Q8CJH3** | plxnb1 | ECM-affiliated Proteins | Matrisome-associated | 45 | 72 | 43 | -0.31307 | 0.032697 | 0.168002 | 28.38576 | 28.5249 | 28.21184 |
| **Q00780** | col8a1 | Collagens | Core matrisome | 30 | 87 | 30 | 0.350151 | 0.032822 | 0.168002 | 27.65503 | 27.49941 | 27.84956 |
| **P12850** | cxcl1 | Secreted Factors | Matrisome-associated | 7 | 9 | 7 | -0.7635 | 0.032909 | 0.168002 | 25.59898 | 25.93831 | 25.17481 |
| **Q9D154** | serpinb1a | ECM Regulators | Matrisome-associated | 13 | 24 | 9 | -0.86149 | 0.032915 | 0.168002 | 25.79746 | 26.18034 | 25.31885 |
| **Q9CQ58** | prl8a9 | Secreted Factors | Matrisome-associated | 7 | 11 | 7 | 0.883433 | 0.033461 | 0.169119 | 25.58592 | 25.19329 | 26.07672 |
| **Q6DFY8** | brinp2 | Secreted Factors | Matrisome-associated | 41 | 71 | 37 | 0.388084 | 0.033479 | 0.169119 | 28.00927 | 27.83679 | 28.22487 |
| **Q8R0S6** | wfikkn1 | Secreted Factors | Matrisome-associated | 11 | 21 | 10 | -0.96092 | 0.033929 | 0.170514 | 23.91047 | 24.33754 | 23.37663 |
| **P98063** | bmp1 | ECM Regulators | Matrisome-associated | 37 | 90 | 32 | -0.27009 | 0.035031 | 0.173817 | 28.05099 | 28.17102 | 27.90094 |
| **Q9JL15** | lgals8 | ECM-affiliated Proteins | Matrisome-associated | 16 | 55 | 16 | -0.45546 | 0.035067 | 0.173817 | 27.12721 | 27.32964 | 26.87418 |
| **Q9D708** | s100a16 | Secreted Factors | Matrisome-associated | 8 | 13 | 8 | 1.029752 | 0.035213 | 0.173817 | 26.63691 | 26.17924 | 27.20899 |
| **P43027** | gdf5 | Secreted Factors | Matrisome-associated | 30 | 51 | 30 | 0.49908 | 0.035465 | 0.173817 | 27.62627 | 27.40445 | 27.90353 |
| **Q8CF98** | colec10 | ECM-affiliated Proteins | Matrisome-associated | 13 | 35 | 13 | 0.565151 | 0.035473 | 0.173817 | 27.16075 | 26.90957 | 27.47472 |
| **Q99K41** | emilin1 | ECM Glycoproteins | Core matrisome | 54 | 179 | 54 | 0.735848 | 0.03578 | 0.174154 | 30.37757 | 30.05053 | 30.78638 |
| **P29621** | serpina3c | ECM Regulators | Matrisome-associated | 18 | 25 | 16 | 0.963826 | 0.035897 | 0.174154 | 28.43301 | 28.00464 | 28.96847 |
| **O70300** | pspn | Secreted Factors | Matrisome-associated | 5 | 14 | 5 | 1.964221 | 0.036477 | 0.176094 | 21.53078 | 20.6578 | 22.62202 |
| **P34821** | bmp8a | Secreted Factors | Matrisome-associated | 23 | 47 | 18 | 0.550182 | 0.037147 | 0.178399 | 26.56202 | 26.31749 | 26.86767 |
| **P97347** | rptn | Secreted Factors | Matrisome-associated | 25 | 43 | 25 | -0.61922 | 0.037318 | 0.178399 | 27.72736 | 28.00257 | 27.38335 |
| **Q60847** | col12a1 | Collagens | Core matrisome | 118 | 242 | 118 | -0.26782 | 0.03791 | 0.180347 | 31.18688 | 31.30591 | 31.03809 |
| **P53347** | osm | Secreted Factors | Matrisome-associated | 12 | 35 | 12 | -1.21041 | 0.038485 | 0.182201 | 26.41553 | 26.95349 | 25.74308 |
| **O35227** | adam7 | ECM Regulators | Matrisome-associated | 33 | 69 | 33 | -0.38029 | 0.038675 | 0.182219 | 26.9317 | 27.10072 | 26.72042 |
| **Q9WU72** | tnfsf13b | Secreted Factors | Matrisome-associated | 12 | 21 | 12 | -0.62982 | 0.038958 | 0.182672 | 23.76534 | 24.04526 | 23.41544 |
| **Q01339** | apoh | ECM-affiliated Proteins | Matrisome-associated | 13 | 21 | 13 | -0.91403 | 0.039928 | 0.185469 | 25.53719 | 25.94343 | 25.0294 |
| **Q9Z1P8** | angptl4 | Secreted Factors | Matrisome-associated | 12 | 23 | 12 | 0.692955 | 0.039933 | 0.185469 | 25.98837 | 25.68039 | 26.37334 |
| **Q811Q4** | adam29 | ECM Regulators | Matrisome-associated | 26 | 56 | 26 | -0.47012 | 0.040536 | 0.187131 | 27.47886 | 27.6878 | 27.21769 |
| **Q8BMS2** | spon2 | ECM Glycoproteins | Core matrisome | 9 | 13 | 9 | 0.80869 | 0.040672 | 0.187131 | 24.41943 | 24.06001 | 24.8687 |
| **P22726** | wnt5b | Secreted Factors | Matrisome-associated | 14 | 17 | 11 | 1.959899 | 0.041068 | 0.187751 | 29.07093 | 28.19986 | 30.15976 |
| **O70326** | grem1 | ECM-affiliated Proteins | Matrisome-associated | 13 | 27 | 13 | 0.533062 | 0.04119 | 0.187751 | 26.09363 | 25.85672 | 26.38978 |
| **P01580** | ifng | Secreted Factors | Matrisome-associated | 3 | 3 | 3 | 1.276014 | 0.041659 | 0.188379 | 20.86503 | 20.29791 | 21.57392 |
| **Q6JHY2** | muc19 | ECM-affiliated Proteins | Matrisome-associated | 35 | 122 | 35 | 0.981951 | 0.041892 | 0.188379 | 30.09758 | 29.66116 | 30.64311 |
| **P16675** | ctsa | ECM Regulators | Matrisome-associated | 23 | 57 | 23 | 0.73387 | 0.041917 | 0.188379 | 28.5766 | 28.25044 | 28.98431 |
| **P22727** | wnt6 | Secreted Factors | Matrisome-associated | 17 | 38 | 17 | 0.354556 | 0.042097 | 0.188379 | 25.9911 | 25.83352 | 26.18808 |
| **P29788** | vtn | ECM Glycoproteins | Core matrisome | 12 | 25 | 12 | 1.256572 | 0.042464 | 0.189158 | 26.69624 | 26.13776 | 27.39433 |
| **Q62010** | ovgp1 | ECM-affiliated Proteins | Matrisome-associated | 25 | 54 | 25 | 0.840146 | 0.042667 | 0.189202 | 25.91737 | 25.54398 | 26.38412 |
| **O35464** | sema6a | ECM-affiliated Proteins | Matrisome-associated | 52 | 123 | 51 | -0.22511 | 0.04335 | 0.190581 | 28.84723 | 28.94728 | 28.72217 |
| **Q925I7** | pdgfd | Secreted Factors | Matrisome-associated | 16 | 33 | 16 | 0.621111 | 0.043367 | 0.190581 | 26.15178 | 25.87573 | 26.49684 |
| **Q68FM6** | elfn2 | ECM-affiliated Proteins | Matrisome-associated | 28 | 57 | 27 | -0.56362 | 0.043943 | 0.19225 | 28.15008 | 28.40058 | 27.83696 |
| **Q9EPL6** | mmp1b | ECM Regulators | Matrisome-associated | 12 | 18 | 5 | -0.61616 | 0.044464 | 0.192347 | 25.03444 | 25.30828 | 24.69213 |
| **P70378** | fgf11 | Secreted Factors | Matrisome-associated | 14 | 37 | 14 | 0.411882 | 0.044586 | 0.192347 | 26.48763 | 26.30458 | 26.71646 |
| **O70370** | ctss | ECM Regulators | Matrisome-associated | 15 | 52 | 15 | 0.336441 | 0.044625 | 0.192347 | 27.94557 | 27.79604 | 28.13248 |
| **Q640N1** | aebp1 | ECM Glycoproteins | Core matrisome | 36 | 66 | 36 | -0.6178 | 0.04475 | 0.192347 | 29.19489 | 29.46946 | 28.85166 |
| **P51942** | matn1 | ECM Glycoproteins | Core matrisome | 21 | 45 | 21 | 0.480822 | 0.044956 | 0.19239 | 27.68798 | 27.47428 | 27.9551 |
| **Q3UQ22** | ntn5 | ECM Glycoproteins | Core matrisome | 23 | 39 | 23 | 0.373525 | 0.04539 | 0.1934 | 26.38736 | 26.22135 | 26.59488 |
| **P10761** | zp3 | ECM Glycoproteins | Core matrisome | 10 | 24 | 10 | -0.40539 | 0.046485 | 0.197208 | 26.8252 | 27.00538 | 26.59999 |
| **P05555** | itgam | ECM-affiliated Proteins | Matrisome-associated | 49 | 111 | 48 | -0.88296 | 0.047133 | 0.199096 | 30.07413 | 30.46655 | 29.58359 |
| **O88207** | col5a1 | Collagens | Core matrisome | 139 | 535 | 127 | -0.36851 | 0.047481 | 0.199703 | 30.38025 | 30.54403 | 30.17553 |
| **Q69Z28** | adamts16 | ECM Regulators | Matrisome-associated | 51 | 103 | 50 | -0.5698 | 0.04773 | 0.199894 | 29.79996 | 30.05321 | 29.48341 |
| **B2RPV6** | mmrn1 | ECM Glycoproteins | Core matrisome | 45 | 68 | 45 | 0.818631 | 0.04854 | 0.202421 | 29.96164 | 29.5978 | 30.41643 |
| **P59384** | adamts15 | ECM Regulators | Matrisome-associated | 47 | 142 | 47 | -0.69102 | 0.049296 | 0.204703 | 29.74552 | 30.05264 | 29.36162 |
| **P11088** | flg | Secreted Factors | Matrisome-associated | 15 | 33 | 15 | -0.39424 | 0.049606 | 0.205122 | 25.99649 | 26.1717 | 25.77747 |
| **Q02788** | col6a2 | Collagens | Core matrisome | 77 | 310 | 77 | -0.15221 | 0.050846 | 0.208975 | 30.034 | 30.10165 | 29.94944 |
| **P13609** | srgn | Proteoglycans | Core matrisome | 3 | 5 | 3 | 1.126409 | 0.051022 | 0.208975 | 22.32829 | 21.82767 | 22.95408 |
| **Q8BZH1** | tgm4 | ECM Regulators | Matrisome-associated | 26 | 43 | 26 | -0.66493 | 0.051178 | 0.208975 | 27.19245 | 27.48798 | 26.82305 |
| **Q91YE3** | egln1 | ECM Regulators | Matrisome-associated | 23 | 63 | 23 | 0.31871 | 0.05254 | 0.213648 | 27.39704 | 27.2554 | 27.57411 |
| **Q60715** | p4ha1 | ECM Regulators | Matrisome-associated | 35 | 61 | 35 | -0.27422 | 0.052879 | 0.214053 | 27.75437 | 27.87624 | 27.60203 |
| **Q9QYS1** | wnt16 | Secreted Factors | Matrisome-associated | 14 | 31 | 14 | -1.43376 | 0.053076 | 0.214053 | 27.15756 | 27.79479 | 26.36103 |
| **P21981** | tgm2 | ECM Regulators | Matrisome-associated | 30 | 47 | 30 | -0.35169 | 0.053453 | 0.214587 | 28.84583 | 29.00214 | 28.65045 |
| **P70206** | plxna1 | ECM-affiliated Proteins | Matrisome-associated | 71 | 176 | 63 | -0.53602 | 0.05385 | 0.214587 | 30.59939 | 30.83762 | 30.3016 |
| **Q9R0S3** | mmp17 | ECM Regulators | Matrisome-associated | 13 | 37 | 13 | 0.531429 | 0.054309 | 0.214587 | 25.68909 | 25.4529 | 25.98433 |
| **P28301** | lox | ECM Regulators | Matrisome-associated | 18 | 51 | 18 | 0.564396 | 0.054392 | 0.214587 | 27.59611 | 27.34526 | 27.90966 |
| **Q91X72** | hpx | ECM-affiliated Proteins | Matrisome-associated | 14 | 18 | 14 | -1.05044 | 0.054567 | 0.214587 | 24.53143 | 24.99829 | 23.94785 |
| **P09055** | itgb1 | ECM-affiliated Proteins | Matrisome-associated | 32 | 52 | 32 | -0.54769 | 0.054705 | 0.214587 | 25.92803 | 26.17144 | 25.62375 |
| **P97400** | clc | ECM-affiliated Proteins | Matrisome-associated | 2 | 5 | 2 | 0.768458 | 0.054848 | 0.214587 | 22.5896 | 22.24806 | 23.01652 |
| **P59511** | adamts20 | ECM Regulators | Matrisome-associated | 58 | 145 | 58 | 0.175047 | 0.055483 | 0.214587 | 29.15809 | 29.08029 | 29.25534 |
| **Q9WUQ5** | cxcl14 | Secreted Factors | Matrisome-associated | 7 | 11 | 7 | -1.96572 | 0.055803 | 0.214587 | 26.62795 | 27.5016 | 25.53588 |
| **P28293** | ctsg | ECM Regulators | Matrisome-associated | 9 | 15 | 9 | -0.64441 | 0.055805 | 0.214587 | 23.69925 | 23.98566 | 23.34124 |
| **P97953** | vegfc | Secreted Factors | Matrisome-associated | 18 | 36 | 18 | -0.51253 | 0.055811 | 0.214587 | 27.42524 | 27.65303 | 27.14049 |
| **Q9D8U4** | c1qtnf2 | ECM-affiliated Proteins | Matrisome-associated | 15 | 30 | 15 | 0.50992 | 0.055837 | 0.214587 | 27.51541 | 27.28878 | 27.7987 |
| **Q60677** | itgae | ECM-affiliated Proteins | Matrisome-associated | 29 | 51 | 29 | -0.55094 | 0.056287 | 0.215301 | 27.00533 | 27.2502 | 26.69926 |
| **O54974** | lgals7 | ECM-affiliated Proteins | Matrisome-associated | 8 | 11 | 8 | 1.017353 | 0.056462 | 0.215301 | 28.31501 | 27.86285 | 28.88021 |
| **O89098** | cst7 | ECM Regulators | Matrisome-associated | 5 | 10 | 5 | -0.94607 | 0.056973 | 0.216411 | 25.9105 | 26.33097 | 25.38491 |
| **Q9R157** | adam18 | ECM Regulators | Matrisome-associated | 21 | 45 | 21 | 0.916852 | 0.057556 | 0.217771 | 26.10486 | 25.69737 | 26.61422 |
| **Q9WUA1** | wif1 | Secreted Factors | Matrisome-associated | 16 | 28 | 16 | 0.606538 | 0.057776 | 0.217771 | 23.86257 | 23.593 | 24.19954 |
| **P51670** | ccl9 | Secreted Factors | Matrisome-associated | 3 | 4 | 3 | -0.68749 | 0.058673 | 0.219444 | 24.18723 | 24.49278 | 23.80529 |
| **P24063** | itgal | ECM-affiliated Proteins | Matrisome-associated | 44 | 92 | 44 | 0.860578 | 0.058729 | 0.219444 | 29.40822 | 29.02574 | 29.88632 |
| **O88632** | sema3f | ECM-affiliated Proteins | Matrisome-associated | 46 | 75 | 45 | -0.66287 | 0.058962 | 0.219444 | 26.49408 | 26.78869 | 26.12582 |
| **Q04997** | inha | Secreted Factors | Matrisome-associated | 11 | 15 | 11 | -1.13644 | 0.059115 | 0.219444 | 22.19276 | 22.69784 | 21.5614 |
| **Q8C8H8** | ky | ECM Regulators | Matrisome-associated | 33 | 66 | 33 | -0.2538 | 0.059532 | 0.220156 | 29.43973 | 29.55253 | 29.29873 |
| **F7A4A7** | otogl | ECM Glycoproteins | Core matrisome | 60 | 109 | 60 | 0.206837 | 0.060225 | 0.221883 | 30.34286 | 30.25093 | 30.45777 |
| **P20722** | bmp6 | Secreted Factors | Matrisome-associated | 13 | 26 | 13 | 1.008861 | 0.061052 | 0.223461 | 28.8907 | 28.44232 | 29.45118 |
| **Q9R0E2** | plod1 | ECM Regulators | Matrisome-associated | 31 | 64 | 30 | -0.30415 | 0.06111 | 0.223461 | 29.57544 | 29.71061 | 29.40647 |
| **P09535** | igf2 | Secreted Factors | Matrisome-associated | 6 | 13 | 6 | -1.80621 | 0.061356 | 0.223528 | 27.49934 | 28.3021 | 26.49589 |
| **P06804** | tnf | Secreted Factors | Matrisome-associated | 11 | 26 | 11 | 0.593853 | 0.062417 | 0.225069 | 26.41891 | 26.15498 | 26.74883 |
| **Q05793** | hspg2 | Proteoglycans | Core matrisome | 105 | 187 | 105 | 0.443788 | 0.062662 | 0.225069 | 28.89409 | 28.69685 | 29.14064 |
| **Q9ESL9** | fgf20 | Secreted Factors | Matrisome-associated | 8 | 14 | 8 | 0.579101 | 0.062834 | 0.225069 | 26.35465 | 26.09727 | 26.67638 |
| **Q4LFA9** | sema3g | ECM-affiliated Proteins | Matrisome-associated | 31 | 161 | 31 | -0.29469 | 0.062993 | 0.225069 | 32.25246 | 32.38343 | 32.08874 |
| **Q9Z2H6** | clec4d | ECM-affiliated Proteins | Matrisome-associated | 3 | 7 | 3 | 0.430854 | 0.0632 | 0.225069 | 24.24434 | 24.05285 | 24.4837 |
| **Q9EPW4** | clec3a | ECM-affiliated Proteins | Matrisome-associated | 5 | 5 | 5 | -1.89112 | 0.063266 | 0.225069 | 22.67517 | 23.51566 | 21.62455 |
| **P07351** | ifna4 | Secreted Factors | Matrisome-associated | 7 | 10 | 7 | -1.81107 | 0.063387 | 0.225069 | 26.89568 | 27.7006 | 25.88952 |
| **Q66K08** | cilp | ECM Glycoproteins | Core matrisome | 41 | 85 | 41 | -0.86104 | 0.063665 | 0.225241 | 28.59446 | 28.97715 | 28.1161 |
| **Q66PY1** | scube3 | Secreted Factors | Matrisome-associated | 27 | 45 | 26 | -0.96624 | 0.06411 | 0.225575 | 29.06284 | 29.49228 | 28.52604 |
| **P14106** | c1qb | ECM-affiliated Proteins | Matrisome-associated | 20 | 41 | 19 | -0.44824 | 0.06422 | 0.225575 | 27.20499 | 27.40421 | 26.95597 |
| **Q9D7I9** | tgm5 | ECM Regulators | Matrisome-associated | 16 | 34 | 16 | -0.65211 | 0.064647 | 0.225937 | 26.19065 | 26.48048 | 25.82837 |
| **Q9JIL2** | ccl28 | Secreted Factors | Matrisome-associated | 3 | 3 | 3 | -1.26636 | 0.064939 | 0.225937 | 24.37692 | 24.93974 | 23.67338 |
| **Q9JK88** | serpini2 | ECM Regulators | Matrisome-associated | 13 | 13 | 13 | -0.66207 | 0.065161 | 0.225937 | 25.17065 | 25.46491 | 24.80283 |
| **Q8CJ91** | cd209b | ECM-affiliated Proteins | Matrisome-associated | 10 | 20 | 10 | -3.05043 | 0.065245 | 0.225937 | 24.8291 | 26.18485 | 23.13442 |
| **O09126** | sema4d | ECM-affiliated Proteins | Matrisome-associated | 26 | 42 | 26 | -1.46772 | 0.065574 | 0.226275 | 27.79608 | 28.4484 | 26.98068 |
| **E9PV24** | fga | ECM Glycoproteins | Core matrisome | 32 | 48 | 32 | -0.40254 | 0.065911 | 0.22664 | 28.14435 | 28.32326 | 27.92072 |
| **P13020** | gsn | Secreted Factors | Matrisome-associated | 24 | 45 | 24 | -0.37197 | 0.066258 | 0.226773 | 27.17822 | 27.34354 | 26.97157 |
| **P48615** | wnt11 | Secreted Factors | Matrisome-associated | 17 | 35 | 17 | 0.740762 | 0.066412 | 0.226773 | 27.50143 | 27.17221 | 27.91297 |
| **P49766** | vegfb | Secreted Factors | Matrisome-associated | 10 | 16 | 10 | -1.38582 | 0.067107 | 0.228352 | 26.18886 | 26.80478 | 25.41896 |
| **Q8C8N3** | vwc2 | Secreted Factors | Matrisome-associated | 6 | 8 | 6 | -0.52847 | 0.067502 | 0.228901 | 23.8173 | 24.05217 | 23.5237 |
| **Q9R1A3** | ntn3 | ECM Glycoproteins | Core matrisome | 25 | 44 | 25 | -0.23049 | 0.067993 | 0.229117 | 27.09385 | 27.19629 | 26.9658 |
| **Q62426** | cstb | ECM Regulators | Matrisome-associated | 7 | 8 | 7 | 1.092468 | 0.068041 | 0.229117 | 21.67706 | 21.19152 | 22.28399 |
| **Q04592** | pcsk5 | ECM Regulators | Matrisome-associated | 53 | 71 | 53 | -0.40559 | 0.068268 | 0.229117 | 26.12599 | 26.30625 | 25.90066 |
| **Q9R0B6** | lamc3 | ECM Glycoproteins | Core matrisome | 42 | 60 | 42 | -0.27269 | 0.068941 | 0.229866 | 27.86677 | 27.98796 | 27.71527 |
| **Q07104** | gdf3 | Secreted Factors | Matrisome-associated | 17 | 29 | 17 | -0.57641 | 0.068972 | 0.229866 | 25.47405 | 25.73023 | 25.15383 |
| **Q8K4G1** | ltbp4 | ECM Glycoproteins | Core matrisome | 65 | 122 | 64 | 0.45572 | 0.069405 | 0.229866 | 27.78668 | 27.58414 | 28.03986 |
| **Q9QYH9** | tnfsf14 | Secreted Factors | Matrisome-associated | 11 | 29 | 11 | -0.42059 | 0.069429 | 0.229866 | 25.14977 | 25.33671 | 24.91611 |
| **P21658** | fgf6 | Secreted Factors | Matrisome-associated | 14 | 28 | 14 | -0.45978 | 0.069763 | 0.230195 | 27.57189 | 27.77624 | 27.31646 |
| **Q9WVH9** | fbln5 | ECM Glycoproteins | Core matrisome | 12 | 33 | 12 | -0.65876 | 0.070989 | 0.233455 | 25.26908 | 25.56186 | 24.9031 |
| **P19788** | mgp | ECM Glycoproteins | Core matrisome | 5 | 10 | 5 | 0.575812 | 0.07186 | 0.23472 | 23.90922 | 23.6533 | 24.22911 |
| **Q9D7D2** | serpina9 | ECM Regulators | Matrisome-associated | 25 | 46 | 25 | 0.674455 | 0.072155 | 0.23472 | 27.23655 | 26.93679 | 27.61125 |
| **Q60716** | p4ha2 | ECM Regulators | Matrisome-associated | 25 | 31 | 25 | -0.49164 | 0.072248 | 0.23472 | 26.70378 | 26.92228 | 26.43065 |
| **Q80X76** | serpina3f | ECM Regulators | Matrisome-associated | 18 | 48 | 14 | -0.39012 | 0.072332 | 0.23472 | 29.93925 | 30.11264 | 29.72252 |
| **Q9R013** | ctsf | ECM Regulators | Matrisome-associated | 16 | 34 | 16 | 0.408615 | 0.073383 | 0.236348 | 28.28726 | 28.10566 | 28.51427 |
| **Q149M0** | clec12b | ECM-affiliated Proteins | Matrisome-associated | 9 | 26 | 9 | 0.628017 | 0.073479 | 0.236348 | 28.73758 | 28.45846 | 29.08648 |
| **Q04841** | mpg | Secreted Factors | Matrisome-associated | 15 | 36 | 15 | -0.53954 | 0.073557 | 0.236348 | 28.39127 | 28.63106 | 28.09153 |
| **P39061** | col18a1 | Collagens | Core matrisome | 76 | 174 | 76 | -0.28226 | 0.074155 | 0.237491 | 28.91436 | 29.03981 | 28.75755 |
| **Q9R158** | adam26a | ECM Regulators | Matrisome-associated | 16 | 48 | 16 | -0.57433 | 0.074702 | 0.238462 | 26.39166 | 26.64692 | 26.07259 |
| **Q8VCS3** | fam20b | ECM Regulators | Matrisome-associated | 21 | 36 | 21 | 0.578582 | 0.075321 | 0.239103 | 25.99864 | 25.7415 | 26.32008 |
| **P08121** | col3a1 | Collagens | Core matrisome | 150 | 766 | 149 | -0.14551 | 0.075391 | 0.239103 | 31.2904 | 31.35507 | 31.20956 |
| **P20181** | ntf3 | Secreted Factors | Matrisome-associated | 12 | 28 | 12 | 0.481681 | 0.076032 | 0.239746 | 26.53561 | 26.32153 | 26.80321 |
| **P61939** | serpina7 | ECM Regulators | Matrisome-associated | 9 | 13 | 9 | 0.454413 | 0.076083 | 0.239746 | 25.88076 | 25.6788 | 26.13321 |
| **Q8C088** | egfem1 | ECM Glycoproteins | Core matrisome | 11 | 21 | 11 | 0.55436 | 0.078449 | 0.245877 | 25.99455 | 25.74816 | 26.30252 |
| **A2AED3** | fndc7 | ECM Glycoproteins | Core matrisome | 17 | 27 | 17 | -0.55525 | 0.078551 | 0.245877 | 24.38461 | 24.63139 | 24.07614 |
| **O88947** | f10 | ECM Regulators | Matrisome-associated | 22 | 45 | 22 | 0.278274 | 0.078781 | 0.245877 | 27.66845 | 27.54477 | 27.82304 |
| **O54890** | itgb3 | ECM-affiliated Proteins | Matrisome-associated | 29 | 43 | 29 | -1.27758 | 0.079555 | 0.247504 | 26.32213 | 26.88994 | 25.61236 |
| **Q9QZF2** | gpc1 | ECM-affiliated Proteins | Matrisome-associated | 21 | 39 | 21 | 0.463592 | 0.080405 | 0.248327 | 26.78427 | 26.57823 | 27.04182 |
| **P01587** | csf2 | Secreted Factors | Matrisome-associated | 3 | 3 | 3 | -0.29534 | 0.08052 | 0.248327 | 25.35519 | 25.48645 | 25.19111 |
| **Q9WTM3** | sema6c | ECM-affiliated Proteins | Matrisome-associated | 36 | 72 | 36 | 0.288125 | 0.08058 | 0.248327 | 26.78092 | 26.65287 | 26.94099 |
| **D3YXG0** | hmcn1 | ECM Glycoproteins | Core matrisome | 176 | 349 | 176 | 0.344851 | 0.081857 | 0.251473 | 31.28791 | 31.13465 | 31.4795 |
| **Q8BLI0** | adamtsl1 | ECM Regulators | Matrisome-associated | 65 | 156 | 65 | 0.195101 | 0.082381 | 0.252293 | 29.34625 | 29.25954 | 29.45464 |
| **Q5FW85** | ecm2 | ECM Glycoproteins | Core matrisome | 22 | 44 | 22 | -0.57758 | 0.083079 | 0.253637 | 26.29831 | 26.55501 | 25.97744 |
| **O08859** | tnfaip6 | ECM Glycoproteins | Core matrisome | 10 | 18 | 10 | -0.49163 | 0.083805 | 0.254043 | 26.07718 | 26.29568 | 25.80405 |
| **Q19LI2** | a1bg | Secreted Factors | Matrisome-associated | 23 | 33 | 23 | -0.62081 | 0.083856 | 0.254043 | 26.44748 | 26.72339 | 26.10258 |
| **P12804** | fgl2 | ECM Glycoproteins | Core matrisome | 22 | 41 | 22 | -0.57486 | 0.08399 | 0.254043 | 27.04458 | 27.30007 | 26.72521 |
| **Q99KC8** | vwa5a | ECM Glycoproteins | Core matrisome | 26 | 67 | 26 | -0.98326 | 0.086097 | 0.258985 | 29.26458 | 29.70158 | 28.71833 |
| **P32766** | cst8 | ECM Regulators | Matrisome-associated | 11 | 22 | 11 | 0.434252 | 0.086224 | 0.258985 | 24.78032 | 24.58732 | 25.02157 |
| **Q61207** | psap | Secreted Factors | Matrisome-associated | 23 | 49 | 23 | 0.872646 | 0.086416 | 0.258985 | 27.91829 | 27.53045 | 28.4031 |
| **Q9CQV3** | serpinb11 | ECM Regulators | Matrisome-associated | 16 | 41 | 16 | -1.29946 | 0.088315 | 0.263869 | 29.58976 | 30.16729 | 28.86783 |
| **Q00623** | apoa1 | ECM-affiliated Proteins | Matrisome-associated | 8 | 13 | 6 | -0.71606 | 0.091862 | 0.273503 | 24.22433 | 24.54258 | 23.82652 |
| **Q9QXH4** | itgax | ECM-affiliated Proteins | Matrisome-associated | 33 | 50 | 29 | -0.30534 | 0.092098 | 0.273503 | 29.67732 | 29.81303 | 29.50769 |
| **Q7TS55** | tnfsf18 | Secreted Factors | Matrisome-associated | 5 | 6 | 5 | -0.87435 | 0.09254 | 0.273986 | 21.91415 | 22.30276 | 21.4284 |
| **Q9R229** | bmp10 | Secreted Factors | Matrisome-associated | 10 | 28 | 10 | -0.74715 | 0.09303 | 0.274158 | 23.0999 | 23.43197 | 22.68481 |
| **P10107** | anxa1 | ECM-affiliated Proteins | Matrisome-associated | 17 | 35 | 17 | 0.333314 | 0.093158 | 0.274158 | 25.79473 | 25.64659 | 25.9799 |
| **Q10738** | mmp7 | ECM Regulators | Matrisome-associated | 10 | 15 | 10 | -1.26229 | 0.093452 | 0.274202 | 27.17586 | 27.73688 | 26.47459 |
| **P49764** | pgf | Secreted Factors | Matrisome-associated | 6 | 16 | 6 | -0.88961 | 0.095389 | 0.278229 | 23.92844 | 24.32382 | 23.43422 |
| **O55189** | ambn | ECM Glycoproteins | Core matrisome | 13 | 54 | 13 | 0.48354 | 0.095393 | 0.278229 | 28.90182 | 28.68691 | 29.17045 |
| **P11688** | itga5 | ECM-affiliated Proteins | Matrisome-associated | 25 | 47 | 24 | 0.338496 | 0.095985 | 0.279126 | 27.31651 | 27.16607 | 27.50457 |
| **Q5QNQ9** | col27a1 | Collagens | Core matrisome | 140 | 460 | 140 | -0.16424 | 0.096511 | 0.279826 | 30.9961 | 31.0691 | 30.90486 |
| **Q3V1M1** | igsf10 | ECM Glycoproteins | Core matrisome | 114 | 292 | 113 | -0.37409 | 0.09803 | 0.28256 | 30.85703 | 31.02329 | 30.6492 |
| **Q61282** | acan | Proteoglycans | Core matrisome | 33 | 101 | 33 | -0.26064 | 0.098377 | 0.28256 | 28.15223 | 28.26807 | 28.00743 |
| **Q05A56** | hyal4 | ECM Regulators | Matrisome-associated | 11 | 19 | 11 | 1.126973 | 0.098633 | 0.28256 | 23.03315 | 22.53227 | 23.65924 |
| **P49935** | ctsh | ECM Regulators | Matrisome-associated | 15 | 27 | 15 | 0.80104 | 0.098882 | 0.28256 | 27.76945 | 27.41344 | 28.21448 |
| **Q00897** | serpina1d | ECM Regulators | Matrisome-associated | 11 | 21 | 5 | 0.338878 | 0.099008 | 0.28256 | 28.4419 | 28.29128 | 28.63016 |
| **O09114** | ptgds | ECM-affiliated Proteins | Matrisome-associated | 8 | 22 | 8 | -0.48959 | 0.099313 | 0.28256 | 24.54202 | 24.75962 | 24.27003 |
| **Q8R459** | il1f10 | Secreted Factors | Matrisome-associated | 7 | 18 | 7 | 0.75853 | 0.099473 | 0.28256 | 24.86 | 24.52287 | 25.2814 |
| **P07759** | serpina3k | ECM Regulators | Matrisome-associated | 14 | 27 | 10 | 0.500642 | 0.100004 | 0.283249 | 26.87484 | 26.65234 | 27.15298 |
| **Q3UPR9** | sbspon | ECM Glycoproteins | Core matrisome | 8 | 21 | 8 | 0.638512 | 0.100917 | 0.28501 | 23.37347 | 23.08969 | 23.7282 |
| **P17125** | tgfb3 | Secreted Factors | Matrisome-associated | 21 | 26 | 21 | -0.30904 | 0.101295 | 0.285256 | 25.77607 | 25.91342 | 25.60439 |
| **Q61072** | adam9 | ECM Regulators | Matrisome-associated | 32 | 59 | 32 | -0.39442 | 0.10169 | 0.285547 | 26.22297 | 26.39826 | 26.00385 |
| **Q61824** | adam12 | ECM Regulators | Matrisome-associated | 41 | 72 | 41 | -0.27521 | 0.102606 | 0.286524 | 28.89991 | 29.02223 | 28.74702 |
| **Q8K406** | lgi3 | ECM Glycoproteins | Core matrisome | 17 | 29 | 17 | 0.472747 | 0.102622 | 0.286524 | 26.2015 | 25.99139 | 26.46414 |
| **Q8C6Z1** | muc15 | ECM-affiliated Proteins | Matrisome-associated | 12 | 74 | 12 | 0.25371 | 0.103142 | 0.287156 | 28.62976 | 28.517 | 28.77071 |
| **Q9Z0F8** | adam17 | ECM Regulators | Matrisome-associated | 33 | 47 | 33 | 0.252124 | 0.104059 | 0.28889 | 26.64954 | 26.53749 | 26.78961 |
| **Q91V88** | npnt | ECM Glycoproteins | Core matrisome | 23 | 61 | 23 | -0.74215 | 0.104728 | 0.289451 | 28.8743 | 29.20415 | 28.462 |
| **Q61087** | lamb3 | ECM Glycoproteins | Core matrisome | 42 | 82 | 42 | -0.2059 | 0.104852 | 0.289451 | 28.8505 | 28.94201 | 28.7361 |
| **Q9ESS2** | fgf22 | Secreted Factors | Matrisome-associated | 11 | 21 | 11 | 0.760898 | 0.10558 | 0.290641 | 24.29378 | 23.95561 | 24.7165 |
| **C0HKD9** | mfap1b | ECM Glycoproteins | Core matrisome | 19 | 37 | 19 | -0.3884 | 0.107071 | 0.292844 | 26.47632 | 26.64894 | 26.26055 |
| **A6H6E2** | mmrn2 | ECM Glycoproteins | Core matrisome | 35 | 54 | 35 | 0.263883 | 0.107235 | 0.292844 | 26.97762 | 26.86034 | 27.12423 |
| **D3Z7H8** | cilp2 | ECM Glycoproteins | Core matrisome | 48 | 82 | 48 | -0.45583 | 0.107277 | 0.292844 | 29.50304 | 29.70563 | 29.2498 |
| **Q03734** | serpina3m | ECM Regulators | Matrisome-associated | 11 | 29 | 7 | -0.53336 | 0.107958 | 0.292898 | 24.06092 | 24.29797 | 23.76461 |
| **Q6YGZ1** | hpse | ECM Regulators | Matrisome-associated | 21 | 41 | 21 | -0.2606 | 0.10813 | 0.292898 | 27.45392 | 27.56975 | 27.30914 |
| **P07356** | anxa2 | ECM-affiliated Proteins | Matrisome-associated | 16 | 42 | 16 | 0.262177 | 0.108428 | 0.292898 | 29.79757 | 29.68105 | 29.94322 |
| **P02468** | lamc1 | ECM Glycoproteins | Core matrisome | 88 | 210 | 88 | -0.18372 | 0.108492 | 0.292898 | 30.1077 | 30.18935 | 30.00563 |
| **Q61554** | fbn1 | ECM Glycoproteins | Core matrisome | 97 | 172 | 94 | -0.16837 | 0.108865 | 0.293097 | 28.49273 | 28.56756 | 28.39919 |
| **Q61810** | ltbp3 | ECM Glycoproteins | Core matrisome | 42 | 66 | 42 | -0.25937 | 0.109866 | 0.293923 | 27.37097 | 27.48624 | 27.22688 |
| **Q9Z0L2** | artn | Secreted Factors | Matrisome-associated | 11 | 15 | 11 | -0.9457 | 0.109944 | 0.293923 | 25.73779 | 26.1581 | 25.2124 |
| **O08762** | prss12 | ECM Regulators | Matrisome-associated | 38 | 56 | 38 | 0.484001 | 0.110071 | 0.293923 | 29.39236 | 29.17725 | 29.66125 |
| **P05524** | fgf3 | Secreted Factors | Matrisome-associated | 13 | 30 | 13 | -0.46469 | 0.111318 | 0.296444 | 27.56758 | 27.77411 | 27.30942 |
| **P48794** | spam1 | ECM Regulators | Matrisome-associated | 14 | 29 | 14 | 0.419651 | 0.111874 | 0.296663 | 27.88471 | 27.6982 | 28.11785 |
| **O54891** | lgals6 | ECM-affiliated Proteins | Matrisome-associated | 10 | 17 | 8 | 0.770932 | 0.112144 | 0.296663 | 26.16143 | 25.8188 | 26.58973 |
| **Q99JR5** | tinagl1 | ECM Glycoproteins | Core matrisome | 15 | 35 | 15 | 0.766611 | 0.112448 | 0.296663 | 24.38578 | 24.04507 | 24.81168 |
| **Q925S4** | il24 | Secreted Factors | Matrisome-associated | 3 | 5 | 3 | 0.40329 | 0.112611 | 0.296663 | 25.31982 | 25.14058 | 25.54387 |
| **Q8BVD7** | c1qtnf7 | ECM-affiliated Proteins | Matrisome-associated | 18 | 56 | 18 | 0.64147 | 0.113899 | 0.298735 | 27.51668 | 27.23159 | 27.87306 |
| **P25318** | col8a2 | Collagens | Core matrisome | 33 | 109 | 33 | -0.23413 | 0.114007 | 0.298735 | 27.98695 | 28.09101 | 27.85688 |
| **Q62470** | itga3 | ECM-affiliated Proteins | Matrisome-associated | 36 | 74 | 36 | -0.42075 | 0.114331 | 0.298785 | 27.38276 | 27.56976 | 27.14901 |
| **P08226** | apoe | ECM-affiliated Proteins | Matrisome-associated | 21 | 57 | 21 | -0.49005 | 0.114675 | 0.298886 | 26.90388 | 27.12168 | 26.63163 |
| **Q9D7P9** | serpinb12 | ECM Regulators | Matrisome-associated | 17 | 21 | 17 | -0.922 | 0.116339 | 0.301046 | 27.12278 | 27.53256 | 26.61056 |
| **Q6IR41** | c1qtnf6 | ECM-affiliated Proteins | Matrisome-associated | 8 | 25 | 8 | -2.04026 | 0.116448 | 0.301046 | 25.01361 | 25.9204 | 23.88014 |
| **P28666** | mug2 | ECM Regulators | Matrisome-associated | 51 | 90 | 41 | -0.5792 | 0.116613 | 0.301046 | 29.73043 | 29.98785 | 29.40865 |
| **B2RXS4** | plxnb2 | ECM-affiliated Proteins | Matrisome-associated | 55 | 117 | 55 | -0.37816 | 0.116732 | 0.301046 | 28.79206 | 28.96013 | 28.58197 |
| **Q8CFG0** | sulf2 | ECM Regulators | Matrisome-associated | 22 | 75 | 22 | -0.18602 | 0.117393 | 0.301378 | 29.26362 | 29.34629 | 29.16027 |
| **P07214** | sparc | ECM Glycoproteins | Core matrisome | 6 | 10 | 6 | 0.5723 | 0.117476 | 0.301378 | 25.62326 | 25.3689 | 25.9412 |
| **Q924X1** | epgn | Secreted Factors | Matrisome-associated | 4 | 10 | 4 | -0.36233 | 0.117943 | 0.301785 | 26.60974 | 26.77077 | 26.40845 |
| **P04769** | prl7d1 | Secreted Factors | Matrisome-associated | 15 | 32 | 15 | 0.540212 | 0.119129 | 0.304027 | 25.56968 | 25.32959 | 25.8698 |
| **P21841** | sftpc | ECM-affiliated Proteins | Matrisome-associated | 11 | 23 | 11 | -0.37158 | 0.120104 | 0.305719 | 26.51106 | 26.67621 | 26.30463 |
| **Q8BU25** | pamr1 | ECM Regulators | Matrisome-associated | 34 | 42 | 34 | -0.45493 | 0.120781 | 0.306572 | 27.37931 | 27.5815 | 27.12657 |
| **Q9D269** | cst11 | ECM Regulators | Matrisome-associated | 6 | 8 | 6 | -0.75562 | 0.121222 | 0.306572 | 25.4947 | 25.83053 | 25.07491 |
| **Q9DAN8** | cst12 | ECM Regulators | Matrisome-associated | 3 | 3 | 2 | 0.663372 | 0.121378 | 0.306572 | 26.32537 | 26.03054 | 26.69391 |
| **P25785** | timp2 | ECM Regulators | Matrisome-associated | 7 | 13 | 7 | 0.702331 | 0.12331 | 0.310596 | 24.79874 | 24.48659 | 25.18893 |
| **P35242** | sftpa1 | ECM-affiliated Proteins | Matrisome-associated | 12 | 33 | 12 | 0.269856 | 0.123839 | 0.310596 | 29.38996 | 29.27002 | 29.53988 |
| **P33434** | mmp2 | ECM Regulators | Matrisome-associated | 28 | 61 | 28 | 0.455607 | 0.123922 | 0.310596 | 27.27654 | 27.07404 | 27.52965 |
| **Q8K479** | c1qtnf5 | ECM-affiliated Proteins | Matrisome-associated | 11 | 52 | 11 | -0.26312 | 0.125152 | 0.312879 | 25.91539 | 26.03234 | 25.76922 |
| **Q80Z19** | muc2 | ECM-affiliated Proteins | Matrisome-associated | 63 | 106 | 63 | -0.3476 | 0.126129 | 0.313781 | 29.54402 | 29.69851 | 29.35091 |
| **Q61220** | nell2 | ECM Glycoproteins | Core matrisome | 14 | 18 | 14 | 0.425549 | 0.126153 | 0.313781 | 24.88992 | 24.70079 | 25.12634 |
| **Q501P1** | fbln7 | ECM Glycoproteins | Core matrisome | 21 | 35 | 21 | -0.20534 | 0.126618 | 0.314141 | 27.4528 | 27.54406 | 27.33872 |
| **Q91VF6** | col26a1 | Collagens | Core matrisome | 17 | 66 | 17 | -0.70307 | 0.127428 | 0.314656 | 27.50478 | 27.81726 | 27.11418 |
| **Q9WUG6** | insl5 | Secreted Factors | Matrisome-associated | 14 | 20 | 14 | -0.39182 | 0.127468 | 0.314656 | 27.39342 | 27.56756 | 27.17574 |
| **Q8BH27** | megf9 | Secreted Factors | Matrisome-associated | 9 | 16 | 9 | 0.4691 | 0.127921 | 0.31498 | 24.40443 | 24.19594 | 24.66504 |
| **Q9QXP7** | c1qtnf1 | ECM-affiliated Proteins | Matrisome-associated | 11 | 16 | 11 | -0.69906 | 0.129203 | 0.315968 | 25.33258 | 25.64327 | 24.94421 |
| **Q61878** | prg2 | Proteoglycans | Core matrisome | 3 | 5 | 3 | -0.39095 | 0.129343 | 0.315968 | 23.26636 | 23.44012 | 23.04917 |
| **P22777** | serpine1 | ECM Regulators | Matrisome-associated | 15 | 24 | 15 | 0.337524 | 0.129797 | 0.315968 | 25.63152 | 25.48151 | 25.81903 |
| **Q3UQ28** | pxdn | ECM Glycoproteins | Core matrisome | 61 | 110 | 61 | -0.71055 | 0.13028 | 0.315968 | 29.94084 | 30.25663 | 29.54609 |
| **Q6GUQ1** | egfl8 | Secreted Factors | Matrisome-associated | 8 | 18 | 8 | 0.775829 | 0.130399 | 0.315968 | 24.02457 | 23.67975 | 24.45558 |
| **P22599** | serpina1b | ECM Regulators | Matrisome-associated | 9 | 19 | 5 | -0.64348 | 0.130574 | 0.315968 | 21.04359 | 21.32958 | 20.68611 |
| **Q8C9W3** | adamts2 | ECM Regulators | Matrisome-associated | 62 | 140 | 62 | 0.256678 | 0.13086 | 0.315968 | 28.99173 | 28.87765 | 29.13433 |
| **O88310** | itln1 | ECM-affiliated Proteins | Matrisome-associated | 6 | 9 | 6 | -0.62287 | 0.131357 | 0.315968 | 23.13943 | 23.41626 | 22.79339 |
| **Q9ESM3** | hapln2 | Proteoglycans | Core matrisome | 14 | 22 | 14 | 0.639332 | 0.131404 | 0.315968 | 24.92714 | 24.643 | 25.28233 |
| **Q9WVL7** | cxcl15 | Secreted Factors | Matrisome-associated | 5 | 5 | 5 | -0.58976 | 0.131546 | 0.315968 | 22.64229 | 22.90441 | 22.31465 |
| **Q9JJN1** | fgf21 | Secreted Factors | Matrisome-associated | 7 | 18 | 7 | 0.730763 | 0.132264 | 0.316915 | 28.83168 | 28.5069 | 29.23766 |
| **O35181** | nrg3 | Secreted Factors | Matrisome-associated | 25 | 46 | 25 | -0.67062 | 0.133356 | 0.318387 | 29.67291 | 29.97096 | 29.30034 |
| **P10146** | ccl1 | Secreted Factors | Matrisome-associated | 7 | 11 | 7 | -0.39852 | 0.133753 | 0.318387 | 24.98142 | 25.15854 | 24.76001 |
| **Q7TMJ8** | pik3ip1 | Secreted Factors | Matrisome-associated | 10 | 25 | 10 | 0.735241 | 0.133853 | 0.318387 | 24.11983 | 23.79305 | 24.52829 |
| **A2AJ76** | hmcn2 | ECM Glycoproteins | Core matrisome | 179 | 366 | 179 | -0.21483 | 0.134716 | 0.319664 | 31.73507 | 31.83055 | 31.61572 |
| **Q80T91** | megf11 | Secreted Factors | Matrisome-associated | 21 | 37 | 21 | 0.777855 | 0.136961 | 0.324206 | 27.8981 | 27.55239 | 28.33024 |
| **Q8CG19** | ltbp1 | ECM Glycoproteins | Core matrisome | 48 | 90 | 48 | -0.32755 | 0.138518 | 0.327102 | 29.98175 | 30.12733 | 29.79978 |
| **P19324** | serpinh1 | ECM Regulators | Matrisome-associated | 28 | 46 | 28 | -0.40131 | 0.139605 | 0.328878 | 26.99905 | 27.17741 | 26.7761 |
| **Q8BG58** | p4htm | ECM Regulators | Matrisome-associated | 13 | 39 | 13 | -0.33793 | 0.141585 | 0.332301 | 26.90007 | 27.05026 | 26.71233 |
| **P04426** | wnt1 | Secreted Factors | Matrisome-associated | 18 | 28 | 18 | -0.38419 | 0.141736 | 0.332301 | 27.21253 | 27.38328 | 26.9991 |
| **Q9DAY2** | prl8a6 | Secreted Factors | Matrisome-associated | 6 | 19 | 5 | 0.602524 | 0.143282 | 0.334468 | 24.16412 | 23.89634 | 24.49886 |
| **Q80YC5** | f12 | ECM Regulators | Matrisome-associated | 11 | 13 | 11 | 0.33944 | 0.143601 | 0.334468 | 24.29699 | 24.14612 | 24.48556 |
| **Q71M36** | cspg5 | ECM-affiliated Proteins | Matrisome-associated | 23 | 38 | 23 | 0.276167 | 0.143892 | 0.334468 | 26.75299 | 26.63025 | 26.90641 |
| **Q8K419** | lgals4 | ECM-affiliated Proteins | Matrisome-associated | 10 | 31 | 8 | 0.733895 | 0.144026 | 0.334468 | 26.5973 | 26.27113 | 27.00502 |
| **Q8BQH6** | cdcp2 | ECM Glycoproteins | Core matrisome | 5 | 9 | 5 | 0.935172 | 0.144763 | 0.334864 | 26.02086 | 25.60522 | 26.5404 |
| **Q91YE2** | egln2 | ECM Regulators | Matrisome-associated | 18 | 25 | 18 | -0.25646 | 0.14488 | 0.334864 | 26.93525 | 27.04923 | 26.79278 |
| **P10148** | ccl2 | Secreted Factors | Matrisome-associated | 8 | 12 | 8 | -2.1466 | 0.145229 | 0.334882 | 26.06086 | 27.0149 | 24.86831 |
| **Q4ZJM7** | otol1 | ECM Glycoproteins | Core matrisome | 32 | 66 | 32 | -0.17176 | 0.147007 | 0.338185 | 29.13676 | 29.2131 | 29.04134 |
| **Q61847** | mep1b | ECM Regulators | Matrisome-associated | 20 | 40 | 20 | -0.33163 | 0.14795 | 0.339558 | 26.32268 | 26.47007 | 26.13844 |
| **Q60813** | adam1a | ECM Regulators | Matrisome-associated | 26 | 45 | 17 | 0.511699 | 0.151202 | 0.34621 | 27.69238 | 27.46496 | 27.97665 |
| **P51865** | tdgf1 | Secreted Factors | Matrisome-associated | 11 | 16 | 11 | -1.13973 | 0.15195 | 0.347112 | 21.54628 | 22.05283 | 20.9131 |
| **Q8CID3** | fam20a | ECM Regulators | Matrisome-associated | 23 | 49 | 23 | -0.22944 | 0.152896 | 0.347982 | 28.00367 | 28.10564 | 27.87621 |
| **P18340** | cxcl9 | Secreted Factors | Matrisome-associated | 7 | 38 | 7 | -0.28884 | 0.153041 | 0.347982 | 28.76741 | 28.89578 | 28.60694 |
| **Q9QZM3** | clcf1 | Secreted Factors | Matrisome-associated | 10 | 19 | 10 | 0.631956 | 0.15346 | 0.348126 | 28.24467 | 27.9638 | 28.59575 |
| **O54907** | tnfsf12 | Secreted Factors | Matrisome-associated | 4 | 12 | 4 | -0.45966 | 0.156546 | 0.354308 | 27.35617 | 27.56046 | 27.1008 |
| **Q920C1** | chrdl1 | Secreted Factors | Matrisome-associated | 19 | 27 | 19 | 0.4405 | 0.157812 | 0.356114 | 28.98825 | 28.79247 | 29.23297 |
| **P70208** | plxna3 | ECM-affiliated Proteins | Matrisome-associated | 57 | 97 | 49 | 0.148597 | 0.158269 | 0.356114 | 28.47229 | 28.40624 | 28.55484 |
| **Q8R4F1** | ntng2 | ECM Glycoproteins | Core matrisome | 19 | 38 | 19 | -0.36356 | 0.158434 | 0.356114 | 26.11255 | 26.27414 | 25.91058 |
| **Q61838** | pzp | ECM Regulators | Matrisome-associated | 43 | 87 | 43 | 0.23812 | 0.15969 | 0.356638 | 29.88656 | 29.78073 | 30.01885 |
| **Q62386** | il17a | Secreted Factors | Matrisome-associated | 10 | 11 | 10 | -0.3967 | 0.159718 | 0.356638 | 26.99352 | 27.16983 | 26.77313 |
| **Q7TN16** | hhip | Secreted Factors | Matrisome-associated | 38 | 74 | 38 | -0.17059 | 0.16057 | 0.356638 | 28.63361 | 28.70943 | 28.53884 |
| **Q91ZJ9** | hyal1 | ECM Regulators | Matrisome-associated | 6 | 7 | 6 | -1.40792 | 0.160628 | 0.356638 | 26.53573 | 27.16148 | 25.75356 |
| **Q3V3R4** | itga1 | ECM-affiliated Proteins | Matrisome-associated | 46 | 73 | 46 | -0.28902 | 0.160807 | 0.356638 | 26.83986 | 26.96832 | 26.67929 |
| **Q9D236** | htra3 | ECM Regulators | Matrisome-associated | 13 | 58 | 13 | -0.25857 | 0.160871 | 0.356638 | 27.30786 | 27.42278 | 27.16421 |
| **Q3UH93** | plxnd1 | ECM-affiliated Proteins | Matrisome-associated | 76 | 132 | 76 | -0.15821 | 0.161348 | 0.356638 | 28.90503 | 28.97535 | 28.81714 |
| **Q9R098** | hgfac | Secreted Factors | Matrisome-associated | 10 | 27 | 10 | -0.46018 | 0.161579 | 0.356638 | 28.7786 | 28.98313 | 28.52294 |
| **Q04857** | col6a1 | Collagens | Core matrisome | 55 | 178 | 55 | -0.14908 | 0.162274 | 0.357368 | 29.73843 | 29.80469 | 29.65561 |
| **Q71KU9** | fgl1 | ECM Glycoproteins | Core matrisome | 9 | 13 | 9 | 1.497715 | 0.163039 | 0.358247 | 25.10501 | 24.43936 | 25.93707 |
| **Q9R0H2** | emcn | ECM-affiliated Proteins | Matrisome-associated | 14 | 47 | 14 | -0.45634 | 0.163573 | 0.358617 | 26.19977 | 26.40258 | 25.94625 |
| **Q7TSQ1** | clec18a | ECM-affiliated Proteins | Matrisome-associated | 14 | 34 | 14 | -0.7623 | 0.165346 | 0.360065 | 26.65569 | 26.99449 | 26.23219 |
| **Q8BH61** | f13a1 | ECM Regulators | Matrisome-associated | 26 | 50 | 26 | 0.389205 | 0.165358 | 0.360065 | 26.2424 | 26.06942 | 26.45863 |
| **P36363** | fgf7 | Secreted Factors | Matrisome-associated | 6 | 12 | 6 | -0.33479 | 0.165935 | 0.360065 | 23.96516 | 24.11395 | 23.77917 |
| **Q923P0** | col20a1 | Collagens | Core matrisome | 49 | 130 | 49 | 0.409375 | 0.166095 | 0.360065 | 29.35963 | 29.17769 | 29.58706 |
| **O54951** | sema6b | ECM-affiliated Proteins | Matrisome-associated | 30 | 48 | 30 | -0.81725 | 0.166107 | 0.360065 | 28.55472 | 28.91794 | 28.10069 |
| **Q9JI33** | ntn4 | ECM Glycoproteins | Core matrisome | 26 | 54 | 26 | -0.22572 | 0.166438 | 0.360065 | 29.50352 | 29.60384 | 29.37812 |
| **Q9JJS0** | scube2 | Secreted Factors | Matrisome-associated | 36 | 59 | 32 | -0.69255 | 0.166899 | 0.360266 | 27.00415 | 27.31195 | 26.6194 |
| **P70663** | sparcl1 | ECM Glycoproteins | Core matrisome | 26 | 71 | 26 | 0.340756 | 0.167629 | 0.361047 | 28.46284 | 28.3114 | 28.65215 |
| **Q8BKV0** | spock3 | Proteoglycans | Core matrisome | 18 | 27 | 18 | -0.23657 | 0.168632 | 0.362412 | 25.71486 | 25.82 | 25.58343 |
| **P07750** | il4 | Secreted Factors | Matrisome-associated | 6 | 6 | 6 | 0.482098 | 0.169383 | 0.363228 | 21.43299 | 21.21872 | 21.70082 |
| **Q80Y72** | cstl1 | ECM Regulators | Matrisome-associated | 6 | 15 | 6 | -1.3674 | 0.170491 | 0.364271 | 17.29953 | 17.90726 | 16.53986 |
| **P56203** | ctsw | ECM Regulators | Matrisome-associated | 15 | 36 | 15 | 0.870727 | 0.170993 | 0.364271 | 27.043 | 26.65601 | 27.52674 |
| **Q61092** | lamc2 | ECM Glycoproteins | Core matrisome | 48 | 123 | 48 | -0.18172 | 0.171102 | 0.364271 | 27.61765 | 27.69841 | 27.5167 |
| **P19221** | f2 | ECM Regulators | Matrisome-associated | 18 | 35 | 18 | -0.40613 | 0.17139 | 0.364271 | 28.32254 | 28.50304 | 28.09691 |
| **P12023** | app | ECM-affiliated Proteins | Matrisome-associated | 27 | 43 | 27 | 0.258055 | 0.171891 | 0.364271 | 27.05515 | 26.94046 | 27.19851 |
| **P97821** | ctsc | ECM Regulators | Matrisome-associated | 9 | 18 | 9 | 0.308317 | 0.1721 | 0.364271 | 24.67347 | 24.53644 | 24.84476 |
| **P58459** | adamts10 | ECM Regulators | Matrisome-associated | 40 | 57 | 40 | 0.839178 | 0.173684 | 0.366834 | 28.28564 | 27.91267 | 28.75185 |
| **Q8R121** | serpina10 | ECM Regulators | Matrisome-associated | 16 | 29 | 16 | -0.32595 | 0.174235 | 0.367128 | 25.14999 | 25.29486 | 24.96891 |
| **Q91WP0** | masp2 | ECM Regulators | Matrisome-associated | 18 | 34 | 18 | -0.46035 | 0.174592 | 0.367128 | 26.46551 | 26.67011 | 26.20976 |
| **O08523** | tecta | ECM Glycoproteins | Core matrisome | 70 | 124 | 70 | 0.153297 | 0.174948 | 0.367128 | 29.87165 | 29.80352 | 29.95681 |
| **Q07563** | col17a1 | Collagens | Core matrisome | 81 | 259 | 80 | 0.132959 | 0.180662 | 0.378308 | 29.29393 | 29.23484 | 29.3678 |
| **P33435** | mmp13 | ECM Regulators | Matrisome-associated | 11 | 19 | 11 | -0.20714 | 0.183422 | 0.383269 | 28.91732 | 29.00938 | 28.80225 |
| **O35988** | sdc4 | ECM-affiliated Proteins | Matrisome-associated | 8 | 13 | 8 | -0.42785 | 0.184285 | 0.38377 | 24.75736 | 24.94751 | 24.51966 |
| **Q00651** | itga4 | ECM-affiliated Proteins | Matrisome-associated | 30 | 60 | 30 | 0.230346 | 0.184445 | 0.38377 | 26.78579 | 26.68341 | 26.91376 |
| **Q9JHQ0** | anxa9 | ECM-affiliated Proteins | Matrisome-associated | 12 | 21 | 12 | 0.690907 | 0.18526 | 0.38465 | 25.3254 | 25.01833 | 25.70924 |
| **P54130** | fgf9 | Secreted Factors | Matrisome-associated | 7 | 11 | 7 | 0.408746 | 0.187427 | 0.387445 | 24.51466 | 24.333 | 24.74174 |
| **Q4VC17** | adamts18 | ECM Regulators | Matrisome-associated | 33 | 59 | 32 | -0.49492 | 0.187485 | 0.387445 | 29.64264 | 29.86261 | 29.36769 |
| **Q8R054** | srpx2 | ECM Glycoproteins | Core matrisome | 23 | 42 | 23 | 0.253791 | 0.187792 | 0.387445 | 28.23556 | 28.12276 | 28.37655 |
| **Q91V08** | clec2d | ECM-affiliated Proteins | Matrisome-associated | 11 | 14 | 10 | -0.65194 | 0.188461 | 0.388007 | 22.60289 | 22.89265 | 22.24071 |
| **Q9Z0T9** | itgb6 | ECM-affiliated Proteins | Matrisome-associated | 32 | 69 | 32 | -0.37143 | 0.190131 | 0.390626 | 27.45295 | 27.61803 | 27.2466 |
| **Q9D676** | clec2g | ECM-affiliated Proteins | Matrisome-associated | 13 | 26 | 9 | -0.20795 | 0.19102 | 0.39163 | 26.95041 | 27.04283 | 26.83488 |
| **P11835** | itgb2 | ECM-affiliated Proteins | Matrisome-associated | 46 | 95 | 46 | 0.282301 | 0.19203 | 0.392881 | 28.1506 | 28.02513 | 28.30743 |
| **Q05306** | col10a1 | Collagens | Core matrisome | 59 | 186 | 59 | 0.21359 | 0.192816 | 0.393665 | 29.57001 | 29.47508 | 29.68867 |
| **P50228** | cxcl5 | Secreted Factors | Matrisome-associated | 6 | 20 | 6 | 0.513475 | 0.19357 | 0.393947 | 27.21535 | 26.98713 | 27.50061 |
| **P21275** | bmp4 | Secreted Factors | Matrisome-associated | 10 | 10 | 10 | 0.332741 | 0.193758 | 0.393947 | 22.42074 | 22.27285 | 22.60559 |
| **P01586** | il3 | Secreted Factors | Matrisome-associated | 9 | 24 | 9 | -0.33909 | 0.195038 | 0.395729 | 23.94348 | 24.09419 | 23.7551 |
| **Q3U492** | kcp | ECM Glycoproteins | Core matrisome | 34 | 62 | 34 | -0.18738 | 0.195968 | 0.396795 | 27.363 | 27.44628 | 27.2589 |
| **O35632** | hyal2 | ECM Regulators | Matrisome-associated | 11 | 38 | 11 | -0.58249 | 0.197504 | 0.399079 | 26.59843 | 26.85732 | 26.27483 |
| **Q8K1S1** | lgi4 | ECM Glycoproteins | Core matrisome | 17 | 26 | 17 | -0.38272 | 0.198204 | 0.39967 | 24.39771 | 24.56781 | 24.18509 |
| **O88531** | ppt1 | Secreted Factors | Matrisome-associated | 9 | 11 | 9 | -0.49989 | 0.201184 | 0.404847 | 25.59745 | 25.81963 | 25.31974 |
| **O54830** | prl7a1 | Secreted Factors | Matrisome-associated | 7 | 21 | 7 | -0.26955 | 0.203004 | 0.407673 | 25.82939 | 25.9492 | 25.67964 |
| **P19467** | muc13 | ECM-affiliated Proteins | Matrisome-associated | 15 | 52 | 15 | -0.38832 | 0.20459 | 0.410017 | 28.42647 | 28.59905 | 28.21074 |
| **Q8BLY1** | smoc1 | ECM Glycoproteins | Core matrisome | 29 | 71 | 29 | -0.18415 | 0.206464 | 0.412762 | 28.27299 | 28.35483 | 28.17069 |
| **Q62177** | sema3b | ECM-affiliated Proteins | Matrisome-associated | 32 | 75 | 32 | -0.59592 | 0.207126 | 0.412762 | 28.48019 | 28.74504 | 28.14912 |
| **O35468** | wnt9b | Secreted Factors | Matrisome-associated | 11 | 18 | 11 | -0.2921 | 0.207353 | 0.412762 | 25.39526 | 25.52508 | 25.23299 |
| **Q8CGD2** | crispld1 | ECM Glycoproteins | Core matrisome | 23 | 53 | 23 | -0.17767 | 0.207644 | 0.412762 | 28.31401 | 28.39298 | 28.21531 |
| **O35474** | edil3 | ECM Glycoproteins | Core matrisome | 22 | 47 | 22 | -0.30915 | 0.2085 | 0.413624 | 29.90031 | 30.03771 | 29.72856 |
| **E9Q7T7** | chadl | Proteoglycans | Core matrisome | 47 | 109 | 47 | -0.17342 | 0.209308 | 0.414387 | 29.45884 | 29.53592 | 29.3625 |
| **Q640P2** | angptl1 | Secreted Factors | Matrisome-associated | 28 | 59 | 27 | -0.32467 | 0.210187 | 0.415288 | 26.96889 | 27.11318 | 26.78852 |
| **Q8VHI5** | vit | ECM Glycoproteins | Core matrisome | 24 | 48 | 24 | -0.23472 | 0.212531 | 0.417687 | 26.2577 | 26.36202 | 26.12729 |
| **P49182** | serpind1 | ECM Regulators | Matrisome-associated | 17 | 24 | 17 | -0.36357 | 0.212633 | 0.417687 | 27.08591 | 27.2475 | 26.88393 |
| **Q8K1K6** | serpinb10 | ECM Regulators | Matrisome-associated | 10 | 30 | 10 | -1.06 | 0.212679 | 0.417687 | 28.20337 | 28.67448 | 27.61448 |
| **O70283** | wnt2b | Secreted Factors | Matrisome-associated | 19 | 42 | 17 | -0.31467 | 0.215265 | 0.421919 | 26.94503 | 27.08488 | 26.77021 |
| **Q9WU66** | sfrp5 | Secreted Factors | Matrisome-associated | 9 | 13 | 9 | 0.296761 | 0.218053 | 0.42653 | 25.13244 | 25.00055 | 25.29731 |
| **Q8BM88** | ctso | ECM Regulators | Matrisome-associated | 11 | 12 | 11 | -0.94554 | 0.218843 | 0.427223 | 27.32124 | 27.74147 | 26.79594 |
| **Q9WVH6** | angpt4 | Secreted Factors | Matrisome-associated | 20 | 36 | 20 | -0.40877 | 0.221749 | 0.430253 | 26.9548 | 27.13648 | 26.72771 |
| **P47877** | igfbp2 | ECM Glycoproteins | Core matrisome | 19 | 38 | 19 | 0.224735 | 0.222033 | 0.430253 | 28.13079 | 28.0309 | 28.25564 |
| **P10493** | nid1 | ECM Glycoproteins | Core matrisome | 37 | 139 | 37 | 0.258692 | 0.222069 | 0.430253 | 29.05527 | 28.9403 | 29.19899 |
| **Q08879** | fbln1 | ECM Glycoproteins | Core matrisome | 20 | 68 | 20 | -0.54187 | 0.222151 | 0.430253 | 26.25541 | 26.49625 | 25.95437 |
| **P51859** | hdgf | Secreted Factors | Matrisome-associated | 9 | 13 | 9 | -0.24241 | 0.222821 | 0.4307 | 23.56766 | 23.6754 | 23.43299 |
| **Q9D8G5** | reg4 | ECM-affiliated Proteins | Matrisome-associated | 9 | 10 | 9 | -0.51825 | 0.225896 | 0.435119 | 24.29794 | 24.52828 | 24.01003 |
| **Q60997** | dmbt1 | ECM Glycoproteins | Core matrisome | 22 | 50 | 22 | -0.1796 | 0.226206 | 0.435119 | 27.4084 | 27.48822 | 27.30862 |
| **Q9JLN6** | adam28 | ECM Regulators | Matrisome-associated | 27 | 64 | 27 | -0.55615 | 0.22644 | 0.435119 | 27.81441 | 28.06159 | 27.50544 |
| **P97298** | serpinf1 | ECM Regulators | Matrisome-associated | 17 | 38 | 17 | -0.6008 | 0.227135 | 0.435602 | 26.59736 | 26.86438 | 26.26358 |
| **A9Z1V5** | vwa5b1 | ECM Glycoproteins | Core matrisome | 43 | 71 | 43 | 0.18422 | 0.228311 | 0.435702 | 27.80222 | 27.72035 | 27.90457 |
| **Q9D2Q8** | s100a14 | Secreted Factors | Matrisome-associated | 5 | 13 | 5 | 0.880604 | 0.228404 | 0.435702 | 23.7693 | 23.37792 | 24.25853 |
| **Q8VHP7** | serpinb1b | ECM Regulators | Matrisome-associated | 8 | 13 | 7 | 0.604382 | 0.228521 | 0.435702 | 25.06584 | 24.79723 | 25.40161 |
| **P09235** | ifna9 | Secreted Factors | Matrisome-associated | 7 | 16 | 2 | -0.30012 | 0.231625 | 0.440761 | 24.54751 | 24.6809 | 24.38078 |
| **Q61702** | itih1 | ECM Regulators | Matrisome-associated | 31 | 56 | 31 | -0.4414 | 0.232328 | 0.441243 | 29.42099 | 29.61717 | 29.17577 |
| **P01132** | egf | Secreted Factors | Matrisome-associated | 42 | 74 | 42 | -0.39832 | 0.233227 | 0.442094 | 29.48518 | 29.66221 | 29.26389 |
| **Q9R1V7** | adam23 | ECM Regulators | Matrisome-associated | 39 | 78 | 39 | 0.218951 | 0.237983 | 0.450092 | 28.44962 | 28.35231 | 28.57126 |
| **P43406** | itgav | ECM-affiliated Proteins | Matrisome-associated | 41 | 74 | 41 | -0.17972 | 0.238388 | 0.450092 | 28.05386 | 28.13374 | 27.95402 |
| **P97816** | s100g | Secreted Factors | Matrisome-associated | 2 | 2 | 2 | -0.3159 | 0.239174 | 0.450092 | 22.34592 | 22.48632 | 22.17042 |
| **Q03350** | thbs2 | ECM Glycoproteins | Core matrisome | 46 | 95 | 46 | -0.17207 | 0.239284 | 0.450092 | 27.33464 | 27.41112 | 27.23905 |
| **Q9DAZ2** | prl2b1 | Secreted Factors | Matrisome-associated | 7 | 9 | 7 | 0.388662 | 0.242552 | 0.455366 | 26.34644 | 26.1737 | 26.56236 |
| **P41155** | ltb | Secreted Factors | Matrisome-associated | 6 | 7 | 6 | 0.722843 | 0.245187 | 0.458886 | 24.34931 | 24.02804 | 24.75089 |
| **O08999** | ltbp2 | ECM Glycoproteins | Core matrisome | 46 | 135 | 45 | 0.363078 | 0.245364 | 0.458886 | 29.85674 | 29.69537 | 30.05845 |
| **P48540** | gdnf | Secreted Factors | Matrisome-associated | 11 | 35 | 11 | 0.216764 | 0.246323 | 0.459803 | 27.18507 | 27.08873 | 27.30549 |
| **Q8CG65** | sspo | ECM Glycoproteins | Core matrisome | 86 | 175 | 86 | 0.322883 | 0.247954 | 0.461967 | 29.17336 | 29.02985 | 29.35274 |
| **P06869** | plau | ECM Regulators | Matrisome-associated | 20 | 22 | 20 | -0.21244 | 0.249113 | 0.463245 | 25.36384 | 25.45825 | 25.24581 |
| **P97435** | tmprss15 | ECM Regulators | Matrisome-associated | 28 | 57 | 28 | 0.204546 | 0.249743 | 0.463538 | 26.95076 | 26.85985 | 27.0644 |
| **Q9WUU7** | ctsz | ECM Regulators | Matrisome-associated | 6 | 7 | 6 | -0.66958 | 0.252395 | 0.467575 | 23.49006 | 23.78765 | 23.11807 |
| **Q99MQ5** | col25a1 | Collagens | Core matrisome | 75 | 322 | 75 | 0.156889 | 0.255348 | 0.472153 | 30.01677 | 29.94704 | 30.10393 |
| **O35684** | serpini1 | ECM Regulators | Matrisome-associated | 12 | 18 | 12 | -0.24632 | 0.260292 | 0.480389 | 26.15929 | 26.26877 | 26.02245 |
| **P70186** | epyc | Proteoglycans | Core matrisome | 18 | 33 | 18 | 0.36908 | 0.261389 | 0.480673 | 26.40899 | 26.24495 | 26.61403 |
| **Q80YX1** | tnc | ECM Glycoproteins | Core matrisome | 57 | 92 | 57 | -0.13314 | 0.261646 | 0.480673 | 27.04471 | 27.10388 | 26.97074 |
| **P05367** | saa2 | Secreted Factors | Matrisome-associated | 5 | 11 | 3 | 0.50442 | 0.262428 | 0.480673 | 24.35391 | 24.12972 | 24.63414 |
| **Q76KF0** | sema6d | ECM-affiliated Proteins | Matrisome-associated | 47 | 102 | 46 | -0.12386 | 0.262821 | 0.480673 | 28.37337 | 28.42842 | 28.30455 |
| **P21570** | ang | Secreted Factors | Matrisome-associated | 6 | 14 | 6 | -0.40632 | 0.262899 | 0.480673 | 24.35667 | 24.53725 | 24.13094 |
| **Q9R0M3** | srpx | ECM Glycoproteins | Core matrisome | 21 | 56 | 21 | -0.12868 | 0.264224 | 0.482196 | 30.15702 | 30.21421 | 30.08553 |
| **P21274** | bmp2 | Secreted Factors | Matrisome-associated | 13 | 14 | 13 | -0.6178 | 0.265723 | 0.484031 | 23.09624 | 23.37082 | 22.75302 |
| **P08505** | il6 | Secreted Factors | Matrisome-associated | 9 | 15 | 9 | 0.364757 | 0.266845 | 0.485172 | 26.12268 | 25.96056 | 26.32532 |
| **P48614** | wnt10b | Secreted Factors | Matrisome-associated | 28 | 65 | 28 | 0.447175 | 0.267416 | 0.485302 | 29.98896 | 29.79021 | 30.23739 |
| **Q9CRB5** | prl7c1 | Secreted Factors | Matrisome-associated | 6 | 7 | 6 | 0.644485 | 0.267907 | 0.485302 | 24.20449 | 23.91805 | 24.56254 |
| **Q9JJZ5** | egfl6 | Secreted Factors | Matrisome-associated | 31 | 57 | 31 | -0.2197 | 0.268693 | 0.48583 | 25.71152 | 25.80916 | 25.58946 |
| **P70269** | ctse | ECM Regulators | Matrisome-associated | 6 | 21 | 6 | 0.397656 | 0.269577 | 0.48653 | 27.92079 | 27.74406 | 28.14171 |
| **Q9JII2** | prl5a1 | Secreted Factors | Matrisome-associated | 16 | 22 | 16 | -0.19901 | 0.272267 | 0.490482 | 25.57684 | 25.66529 | 25.46628 |
| **P35419** | tpo | Secreted Factors | Matrisome-associated | 36 | 64 | 36 | -0.13786 | 0.273161 | 0.49091 | 28.01773 | 28.079 | 27.94114 |
| **Q9JKV9** | il20 | Secreted Factors | Matrisome-associated | 5 | 5 | 5 | -0.66132 | 0.273507 | 0.49091 | 23.72847 | 24.02239 | 23.36107 |
| **Q9CQ76** | nepn | Proteoglycans | Core matrisome | 27 | 54 | 27 | -0.19903 | 0.274232 | 0.491311 | 28.52562 | 28.61408 | 28.41505 |
| **P27784** | ccl6 | Secreted Factors | Matrisome-associated | 4 | 9 | 4 | 0.505831 | 0.274818 | 0.491463 | 26.43988 | 26.21506 | 26.72089 |
| **Q9EQC7** | fstl3 | Secreted Factors | Matrisome-associated | 4 | 9 | 4 | -0.3177 | 0.276075 | 0.492143 | 23.91097 | 24.05217 | 23.73447 |
| **Q8K0D2** | habp2 | ECM Regulators | Matrisome-associated | 18 | 51 | 18 | -0.17591 | 0.276499 | 0.492143 | 29.74747 | 29.82565 | 29.64974 |
| **P15247** | il9 | Secreted Factors | Matrisome-associated | 5 | 9 | 5 | 0.263773 | 0.276705 | 0.492143 | 25.99042 | 25.87319 | 26.13696 |
| **Q3UW26** | bsph1 | ECM Glycoproteins | Core matrisome | 4 | 5 | 4 | -0.54586 | 0.278714 | 0.494819 | 23.60385 | 23.84646 | 23.3006 |
| **P41274** | tnfsf9 | Secreted Factors | Matrisome-associated | 10 | 20 | 10 | 0.330026 | 0.27938 | 0.495104 | 25.58552 | 25.43884 | 25.76886 |
| **P70379** | fgf14 | Secreted Factors | Matrisome-associated | 19 | 28 | 18 | 0.227815 | 0.280806 | 0.496733 | 26.16679 | 26.06554 | 26.29336 |
| **Q99JG3** | anxa13 | ECM-affiliated Proteins | Matrisome-associated | 20 | 32 | 20 | 0.173607 | 0.281997 | 0.497941 | 26.47077 | 26.39361 | 26.56722 |
| **Q61592** | gas6 | ECM Glycoproteins | Core matrisome | 24 | 35 | 24 | 0.177065 | 0.283231 | 0.499221 | 25.89035 | 25.81166 | 25.98872 |
| **Q8C8T7** | elfn1 | ECM-affiliated Proteins | Matrisome-associated | 22 | 55 | 21 | 0.262626 | 0.285228 | 0.50133 | 27.03155 | 26.91483 | 27.17745 |
| **P56677** | st14 | ECM Regulators | Matrisome-associated | 29 | 73 | 29 | 0.25347 | 0.285451 | 0.50133 | 27.15754 | 27.04489 | 27.29836 |
| **Q8VEA6** | chrdl2 | Secreted Factors | Matrisome-associated | 11 | 19 | 11 | -0.49465 | 0.286974 | 0.503103 | 26.08321 | 26.30306 | 25.80841 |
| **P55066** | ncan | Proteoglycans | Core matrisome | 27 | 50 | 27 | 0.117403 | 0.287991 | 0.503985 | 26.87296 | 26.82078 | 26.93818 |
| **Q8BME9** | cbln4 | Secreted Factors | Matrisome-associated | 7 | 17 | 7 | -0.58614 | 0.292916 | 0.511689 | 22.11131 | 22.37182 | 21.78568 |
| **P70377** | fgf13 | Secreted Factors | Matrisome-associated | 9 | 48 | 9 | 0.310099 | 0.293857 | 0.51242 | 24.61253 | 24.47471 | 24.78481 |
| **P34884** | mif | Secreted Factors | Matrisome-associated | 4 | 13 | 4 | -0.92134 | 0.294695 | 0.512969 | 20.97122 | 21.38071 | 20.45936 |
| **P43488** | tnfsf4 | Secreted Factors | Matrisome-associated | 9 | 19 | 9 | 0.243978 | 0.297231 | 0.516465 | 27.21775 | 27.10932 | 27.3533 |
| **Q80UG2** | plxna4 | ECM-affiliated Proteins | Matrisome-associated | 70 | 113 | 62 | 0.177997 | 0.298622 | 0.517964 | 28.82068 | 28.74157 | 28.91956 |
| **O35103** | omd | Proteoglycans | Core matrisome | 12 | 21 | 12 | -0.22087 | 0.304762 | 0.527212 | 25.23607 | 25.33423 | 25.11336 |
| **Q62059** | vcan | Proteoglycans | Core matrisome | 70 | 133 | 70 | 0.128369 | 0.30503 | 0.527212 | 28.2932 | 28.23614 | 28.36451 |
| **Q6W5C0** | cxcl3 | Secreted Factors | Matrisome-associated | 3 | 8 | 2 | 0.938683 | 0.306121 | 0.528167 | 17.3859 | 16.96871 | 17.90739 |
| **P37804** | tagln | ECM-affiliated Proteins | Matrisome-associated | 11 | 27 | 11 | -0.32858 | 0.306706 | 0.528247 | 25.37393 | 25.51997 | 25.19139 |
| **P22725** | wnt5a | Secreted Factors | Matrisome-associated | 16 | 22 | 12 | -0.53359 | 0.307526 | 0.528728 | 26.81714 | 27.05429 | 26.5207 |
| **P05366** | saa1 | Secreted Factors | Matrisome-associated | 6 | 24 | 3 | -0.20963 | 0.30971 | 0.530655 | 25.55814 | 25.65131 | 25.44167 |
| **Q8R1R4** | il34 | Secreted Factors | Matrisome-associated | 8 | 22 | 8 | 0.539927 | 0.309729 | 0.530655 | 26.66644 | 26.42648 | 26.9664 |
| **Q9D968** | hcfc2 | Secreted Factors | Matrisome-associated | 31 | 69 | 31 | -0.12551 | 0.314982 | 0.538713 | 28.20509 | 28.26087 | 28.13536 |
| **P04401** | il5 | Secreted Factors | Matrisome-associated | 6 | 10 | 6 | -0.37768 | 0.315938 | 0.539406 | 21.94341 | 22.11126 | 21.73358 |
| **P09056** | lif | Secreted Factors | Matrisome-associated | 15 | 28 | 15 | -0.5825 | 0.317426 | 0.541004 | 25.86498 | 26.12387 | 25.54137 |
| **P10749** | il1b | Secreted Factors | Matrisome-associated | 7 | 12 | 7 | -0.92574 | 0.319244 | 0.541348 | 24.7422 | 25.15364 | 24.2279 |
| **Q8VCM7** | fgg | ECM Glycoproteins | Core matrisome | 12 | 23 | 12 | 0.360137 | 0.31947 | 0.541348 | 24.53852 | 24.37846 | 24.7386 |
| **P34928** | apoc1 | ECM-affiliated Proteins | Matrisome-associated | 5 | 8 | 5 | -0.32215 | 0.320591 | 0.541348 | 20.98283 | 21.12601 | 20.80386 |
| **P51910** | apod | ECM-affiliated Proteins | Matrisome-associated | 9 | 14 | 9 | 0.322043 | 0.320842 | 0.541348 | 24.58972 | 24.44659 | 24.76863 |
| **P97463** | nrtn | Secreted Factors | Matrisome-associated | 13 | 27 | 13 | -0.3178 | 0.321383 | 0.541348 | 29.31162 | 29.45287 | 29.13507 |
| **Q9WTR5** | cdh13 | ECM-affiliated Proteins | Matrisome-associated | 22 | 46 | 22 | -0.34496 | 0.321424 | 0.541348 | 29.25309 | 29.4064 | 29.06144 |
| **Q62009** | postn | ECM Glycoproteins | Core matrisome | 42 | 93 | 42 | 0.170069 | 0.321494 | 0.541348 | 29.52517 | 29.44959 | 29.61965 |
| **Q8CD91** | smoc2 | ECM Glycoproteins | Core matrisome | 19 | 27 | 19 | -0.26776 | 0.323881 | 0.544431 | 25.93801 | 26.05702 | 25.78926 |
| **Q08048** | hgf | Secreted Factors | Matrisome-associated | 41 | 73 | 41 | 0.223675 | 0.324692 | 0.54486 | 28.48984 | 28.39043 | 28.61411 |
| **Q7TSK7** | adamtsl2 | ECM Regulators | Matrisome-associated | 36 | 75 | 36 | -0.12683 | 0.327403 | 0.54847 | 27.42912 | 27.48549 | 27.35866 |
| **Q00898** | serpina1e | ECM Regulators | Matrisome-associated | 8 | 33 | 6 | -0.24099 | 0.328533 | 0.549424 | 27.12133 | 27.22843 | 26.98745 |
| **Q07797** | lgals3bp | ECM-affiliated Proteins | Matrisome-associated | 18 | 27 | 18 | 0.35751 | 0.334293 | 0.558104 | 26.67506 | 26.51617 | 26.87368 |
| **O09118** | ntn1 | ECM Glycoproteins | Core matrisome | 20 | 34 | 20 | -0.20793 | 0.335861 | 0.559406 | 25.09411 | 25.18652 | 24.97859 |
| **P06879** | prl | Secreted Factors | Matrisome-associated | 5 | 6 | 5 | -0.50139 | 0.336716 | 0.559406 | 21.27286 | 21.4957 | 20.99431 |
| **Q8VEI3** | hyal3 | ECM Regulators | Matrisome-associated | 8 | 10 | 8 | 0.285049 | 0.336785 | 0.559406 | 25.82512 | 25.69843 | 25.98348 |
| **Q9R182** | angptl3 | Secreted Factors | Matrisome-associated | 30 | 72 | 30 | 0.161173 | 0.338884 | 0.56194 | 28.37591 | 28.30427 | 28.46545 |
| **P86792** | ccl21b | Secreted Factors | Matrisome-associated | 7 | 11 | 7 | -0.48229 | 0.34024 | 0.562543 | 24.61384 | 24.82819 | 24.3459 |
| **P97290** | serping1 | ECM Regulators | Matrisome-associated | 15 | 32 | 15 | 0.230899 | 0.340396 | 0.562543 | 23.9701 | 23.86748 | 24.09838 |
| **O35903** | ccl25 | Secreted Factors | Matrisome-associated | 12 | 25 | 12 | 0.24222 | 0.341451 | 0.563336 | 24.20193 | 24.09428 | 24.3365 |
| **O09107** | insl3 | Secreted Factors | Matrisome-associated | 11 | 24 | 11 | 0.376449 | 0.344142 | 0.565892 | 24.6794 | 24.51209 | 24.88854 |
| **P01573** | ifna2 | Secreted Factors | Matrisome-associated | 11 | 28 | 7 | -0.2873 | 0.344495 | 0.565892 | 26.91452 | 27.04221 | 26.75491 |
| **Q923W9** | adam33 | ECM Regulators | Matrisome-associated | 10 | 21 | 10 | 0.268317 | 0.344732 | 0.565892 | 25.98911 | 25.86985 | 26.13817 |
| **P35175** | stfa1 | ECM Regulators | Matrisome-associated | 3 | 3 | 3 | -0.28263 | 0.347576 | 0.569606 | 24.75194 | 24.87755 | 24.59492 |
| **Q9JL95** | prg3 | Proteoglycans | Core matrisome | 4 | 6 | 4 | 0.176153 | 0.349636 | 0.572026 | 26.94143 | 26.86313 | 27.03929 |
| **Q9D3P1** | tchhl1 | Secreted Factors | Matrisome-associated | 29 | 80 | 29 | -0.16018 | 0.350373 | 0.572276 | 28.80837 | 28.87956 | 28.71938 |
| **Q9WV56** | gdf2 | Secreted Factors | Matrisome-associated | 14 | 26 | 14 | 0.407844 | 0.351512 | 0.57318 | 27.32147 | 27.14021 | 27.54805 |
| **P17553** | wnt3 | Secreted Factors | Matrisome-associated | 7 | 8 | 6 | 0.368059 | 0.355039 | 0.577971 | 21.9027 | 21.73911 | 22.10717 |
| **P21552** | wnt2 | Secreted Factors | Matrisome-associated | 16 | 28 | 14 | -0.2383 | 0.355959 | 0.578507 | 26.20243 | 26.30834 | 26.07004 |
| **Q00896** | serpina1c | ECM Regulators | Matrisome-associated | 13 | 25 | 6 | 0.19752 | 0.357467 | 0.579996 | 26.061 | 25.97321 | 26.17073 |
| **P82198** | tgfbi | ECM Glycoproteins | Core matrisome | 41 | 81 | 41 | -0.18296 | 0.359031 | 0.581571 | 26.89858 | 26.9799 | 26.79694 |
| **Q8VED9** | lgalsl | ECM-affiliated Proteins | Matrisome-associated | 7 | 12 | 7 | 0.37914 | 0.361416 | 0.584467 | 24.28092 | 24.11241 | 24.49155 |
| **P09920** | csf3 | Secreted Factors | Matrisome-associated | 5 | 12 | 5 | 0.560971 | 0.364086 | 0.586907 | 25.43003 | 25.18071 | 25.74168 |
| **P60882** | megf8 | Secreted Factors | Matrisome-associated | 71 | 177 | 71 | -0.16434 | 0.364316 | 0.586907 | 29.24344 | 29.31648 | 29.15214 |
| **P16045** | lgals1 | ECM-affiliated Proteins | Matrisome-associated | 14 | 27 | 14 | -0.24679 | 0.364721 | 0.586907 | 26.77612 | 26.88581 | 26.63902 |
| **Q0VF58** | col19a1 | Collagens | Core matrisome | 93 | 304 | 93 | -0.08572 | 0.368902 | 0.592662 | 30.27183 | 30.30992 | 30.2242 |
| **Q80SS5** | ifna12 | Secreted Factors | Matrisome-associated | 6 | 6 | 3 | 0.228036 | 0.373272 | 0.598329 | 24.54393 | 24.44258 | 24.67062 |
| **Q9QY05** | insl6 | Secreted Factors | Matrisome-associated | 9 | 15 | 9 | -0.19256 | 0.37365 | 0.598329 | 23.52585 | 23.61144 | 23.41888 |
| **Q3U0K8** | ogfod1 | ECM Regulators | Matrisome-associated | 19 | 36 | 19 | 0.211868 | 0.374932 | 0.599402 | 28.65348 | 28.55931 | 28.77118 |
| **O08717** | inhbe | Secreted Factors | Matrisome-associated | 17 | 33 | 17 | 0.263263 | 0.375964 | 0.600073 | 30.12001 | 30.003 | 30.26627 |
| **P26928** | mst1 | Secreted Factors | Matrisome-associated | 31 | 76 | 31 | 0.297013 | 0.378169 | 0.602611 | 27.55945 | 27.42745 | 27.72446 |
| **Q8BYY9** | serpina3b | ECM Regulators | Matrisome-associated | 17 | 27 | 17 | 0.35401 | 0.380076 | 0.604666 | 27.42981 | 27.27247 | 27.62648 |
| **P21844** | cma1 | Secreted Factors | Matrisome-associated | 15 | 37 | 15 | 0.172796 | 0.381557 | 0.605136 | 28.53778 | 28.46098 | 28.63377 |
| **P27005** | s100a8 | Secreted Factors | Matrisome-associated | 3 | 4 | 3 | -0.30563 | 0.382873 | 0.605136 | 22.78828 | 22.92411 | 22.61848 |
| **Q91ZV7** | plxdc1 | ECM-affiliated Proteins | Matrisome-associated | 13 | 26 | 13 | -0.26584 | 0.383698 | 0.605136 | 26.26859 | 26.38674 | 26.1209 |
| **Q920A0** | optc | Proteoglycans | Core matrisome | 12 | 40 | 12 | -0.32591 | 0.384026 | 0.605136 | 26.08144 | 26.22629 | 25.90038 |
| **P23359** | bmp7 | Secreted Factors | Matrisome-associated | 6 | 11 | 5 | -0.23838 | 0.384068 | 0.605136 | 25.20721 | 25.31316 | 25.07477 |
| **P61622** | itga11 | ECM-affiliated Proteins | Matrisome-associated | 52 | 119 | 52 | 0.116608 | 0.38442 | 0.605136 | 29.12178 | 29.06995 | 29.18656 |
| **Q9R1N9** | col13a1 | Collagens | Core matrisome | 59 | 209 | 59 | -0.18772 | 0.384693 | 0.605136 | 29.11738 | 29.20081 | 29.01309 |
| **P21956** | mfge8 | ECM Glycoproteins | Core matrisome | 22 | 40 | 22 | -0.23787 | 0.387307 | 0.608271 | 25.93116 | 26.03688 | 25.79902 |
| **P62818** | s100a3 | Secreted Factors | Matrisome-associated | 5 | 7 | 5 | -0.55361 | 0.389069 | 0.6097 | 22.70289 | 22.94894 | 22.39533 |
| **P31240** | pdgfb | Secreted Factors | Matrisome-associated | 10 | 17 | 10 | 0.890502 | 0.391935 | 0.6097 | 29.087 | 28.69122 | 29.58173 |
| **O08746** | matn2 | ECM Glycoproteins | Core matrisome | 29 | 42 | 29 | -0.13861 | 0.392132 | 0.6097 | 26.87346 | 26.93506 | 26.79645 |
| **Q9ESN4** | c1ql3 | ECM-affiliated Proteins | Matrisome-associated | 17 | 54 | 14 | 0.133958 | 0.392615 | 0.6097 | 26.3262 | 26.26667 | 26.40063 |
| **P54615** | bglap3 | ECM Glycoproteins | Core matrisome | 8 | 12 | 5 | -0.17844 | 0.39268 | 0.6097 | 26.88917 | 26.96848 | 26.79004 |
| **P43432** | il12b | Secreted Factors | Matrisome-associated | 17 | 26 | 17 | 0.127148 | 0.39382 | 0.6097 | 26.73792 | 26.68141 | 26.80855 |
| **Q60519** | sema5b | ECM-affiliated Proteins | Matrisome-associated | 41 | 80 | 41 | 0.133959 | 0.394108 | 0.6097 | 28.31199 | 28.25245 | 28.38641 |
| **Q9JLF6** | tgm1 | ECM Regulators | Matrisome-associated | 35 | 59 | 35 | -0.175 | 0.394186 | 0.6097 | 28.65023 | 28.728 | 28.553 |
| **Q9EQ14** | il23a | Secreted Factors | Matrisome-associated | 6 | 10 | 6 | -0.19398 | 0.394281 | 0.6097 | 24.50365 | 24.58987 | 24.39588 |
| **Q0VBD0** | itgb8 | ECM-affiliated Proteins | Matrisome-associated | 27 | 55 | 27 | 0.177061 | 0.394569 | 0.6097 | 28.75466 | 28.67597 | 28.85303 |
| **Q9R160** | adam24 | ECM Regulators | Matrisome-associated | 21 | 39 | 21 | -0.22149 | 0.395725 | 0.6097 | 28.01667 | 28.11511 | 27.89362 |
| **Q9R0G6** | comp | ECM Glycoproteins | Core matrisome | 35 | 72 | 35 | 0.137754 | 0.396153 | 0.6097 | 26.74882 | 26.6876 | 26.82535 |
| **P48298** | ccl11 | Secreted Factors | Matrisome-associated | 2 | 3 | 2 | 0.296276 | 0.396305 | 0.6097 | 26.48278 | 26.3511 | 26.64738 |
| **Q8VCP9** | clec14a | ECM-affiliated Proteins | Matrisome-associated | 7 | 8 | 7 | 0.418968 | 0.399709 | 0.613973 | 22.87418 | 22.68797 | 23.10694 |
| **Q61789** | lama3 | ECM Glycoproteins | Core matrisome | 124 | 226 | 124 | 0.066828 | 0.400408 | 0.614084 | 30.15194 | 30.12224 | 30.18907 |
| **Q9JHI0** | mmp19 | ECM Regulators | Matrisome-associated | 16 | 32 | 16 | -0.20542 | 0.404482 | 0.619362 | 27.60879 | 27.70009 | 27.49467 |
| **Q80W15** | igfbpl1 | ECM Glycoproteins | Core matrisome | 16 | 39 | 16 | 0.365517 | 0.407125 | 0.622438 | 24.21736 | 24.05491 | 24.42042 |
| **Q9JM84** | cst10 | ECM Regulators | Matrisome-associated | 11 | 24 | 11 | -0.2299 | 0.408349 | 0.622644 | 27.03848 | 27.14065 | 26.91076 |
| **P97299** | sfrp2 | Secreted Factors | Matrisome-associated | 13 | 21 | 13 | -0.19482 | 0.408531 | 0.622644 | 26.18961 | 26.2762 | 26.08138 |
| **P97429** | anxa4 | ECM-affiliated Proteins | Matrisome-associated | 9 | 17 | 9 | 0.2304 | 0.409919 | 0.62379 | 24.8909 | 24.7885 | 25.0189 |
| **Q91X79** | cela1 | ECM Regulators | Matrisome-associated | 6 | 21 | 6 | -0.49436 | 0.41134 | 0.624981 | 24.93668 | 25.15639 | 24.66203 |
| **Q8CGZ9** | prl7b1 | Secreted Factors | Matrisome-associated | 10 | 34 | 10 | -0.4125 | 0.412294 | 0.625 | 28.57182 | 28.75516 | 28.34265 |
| **Q769J6** | adamts13 | ECM Regulators | Matrisome-associated | 39 | 67 | 39 | -0.16313 | 0.412628 | 0.625 | 26.75089 | 26.82339 | 26.66026 |
| **Q9QZ10** | anxa10 | ECM-affiliated Proteins | Matrisome-associated | 8 | 16 | 8 | 0.265369 | 0.414846 | 0.626462 | 24.91598 | 24.79804 | 25.06341 |
| **P70701** | wnt10a | Secreted Factors | Matrisome-associated | 17 | 35 | 17 | -0.13315 | 0.414871 | 0.626462 | 28.40995 | 28.46913 | 28.33598 |
| **O55226** | chad | Proteoglycans | Core matrisome | 26 | 107 | 26 | 0.17756 | 0.416936 | 0.628611 | 27.90525 | 27.82633 | 28.00389 |
| **B8JK39** | itga9 | ECM-affiliated Proteins | Matrisome-associated | 40 | 77 | 40 | 0.155878 | 0.419306 | 0.630674 | 27.85556 | 27.78628 | 27.94216 |
| **Q61739** | itga6 | ECM-affiliated Proteins | Matrisome-associated | 59 | 94 | 59 | 0.21652 | 0.419591 | 0.630674 | 27.03863 | 26.9424 | 27.15892 |
| **O54831** | prl7a2 | Secreted Factors | Matrisome-associated | 6 | 10 | 6 | 0.461469 | 0.421066 | 0.631804 | 24.80226 | 24.59716 | 25.05863 |
| **P97352** | s100a13 | Secreted Factors | Matrisome-associated | 9 | 18 | 8 | -0.18893 | 0.421633 | 0.631804 | 26.90958 | 26.99355 | 26.80461 |
| **P51642** | cntf | Secreted Factors | Matrisome-associated | 6 | 6 | 6 | 0.6251 | 0.425655 | 0.636858 | 25.39769 | 25.11986 | 25.74496 |
| **P48346** | il15 | Secreted Factors | Matrisome-associated | 4 | 9 | 4 | -0.42713 | 0.426843 | 0.637548 | 22.51841 | 22.70825 | 22.28112 |
| **Q61711** | ibsp | ECM Glycoproteins | Core matrisome | 5 | 7 | 5 | -0.32892 | 0.427755 | 0.637548 | 22.72341 | 22.86959 | 22.54067 |
| **Q9JIA1** | lgi1 | ECM Glycoproteins | Core matrisome | 18 | 41 | 18 | 0.166248 | 0.429637 | 0.637548 | 28.27632 | 28.20244 | 28.36868 |
| **O89093** | ccl20 | Secreted Factors | Matrisome-associated | 3 | 11 | 3 | -0.23574 | 0.429643 | 0.637548 | 24.39202 | 24.49679 | 24.26105 |
| **Q9JK53** | prelp | Proteoglycans | Core matrisome | 39 | 90 | 39 | 0.169373 | 0.429907 | 0.637548 | 27.63205 | 27.55678 | 27.72615 |
| **O08677** | kng1 | ECM Regulators | Matrisome-associated | 13 | 23 | 13 | -0.18182 | 0.43002 | 0.637548 | 26.24063 | 26.32144 | 26.13962 |
| **P43028** | gdf6 | Secreted Factors | Matrisome-associated | 12 | 16 | 12 | -0.90412 | 0.432911 | 0.638999 | 20.07018 | 20.47201 | 19.56789 |
| **P15656** | fgf5 | Secreted Factors | Matrisome-associated | 11 | 39 | 11 | 0.10407 | 0.432926 | 0.638999 | 27.22248 | 27.17622 | 27.28029 |
| **Q8R066** | c1qtnf4 | ECM-affiliated Proteins | Matrisome-associated | 21 | 26 | 21 | 0.167642 | 0.432955 | 0.638999 | 26.07369 | 25.99918 | 26.16683 |
| **Q8BFU0** | rspo2 | ECM Glycoproteins | Core matrisome | 8 | 9 | 8 | 0.227823 | 0.435785 | 0.641904 | 22.42664 | 22.32538 | 22.5532 |
| **P98064** | masp1 | ECM Regulators | Matrisome-associated | 22 | 35 | 22 | -0.14095 | 0.43716 | 0.641904 | 27.34765 | 27.4103 | 27.26935 |
| **Q3UP87** | elane | ECM Regulators | Matrisome-associated | 11 | 15 | 11 | -0.1545 | 0.437316 | 0.641904 | 25.86392 | 25.93258 | 25.77809 |
| **P15655** | fgf2 | Secreted Factors | Matrisome-associated | 8 | 12 | 8 | 0.230008 | 0.437543 | 0.641904 | 23.44059 | 23.33836 | 23.56837 |
| **P18893** | il10 | Secreted Factors | Matrisome-associated | 9 | 12 | 9 | -0.30652 | 0.438556 | 0.642429 | 26.1866 | 26.32284 | 26.01631 |
| **P47879** | igfbp4 | ECM Glycoproteins | Core matrisome | 12 | 22 | 12 | 0.306522 | 0.442477 | 0.646868 | 24.77774 | 24.6415 | 24.94802 |
| **Q8BJ73** | rspo4 | ECM Glycoproteins | Core matrisome | 11 | 22 | 11 | -0.15731 | 0.442907 | 0.646868 | 31.79269 | 31.86261 | 31.7053 |
| **O08689** | mstn | Secreted Factors | Matrisome-associated | 20 | 33 | 18 | 0.144945 | 0.446385 | 0.650677 | 26.62072 | 26.5563 | 26.70124 |
| **P30882** | ccl5 | Secreted Factors | Matrisome-associated | 2 | 2 | 2 | 0.293841 | 0.446843 | 0.650677 | 22.39075 | 22.26016 | 22.554 |
| **P70375** | f7 | ECM Regulators | Matrisome-associated | 13 | 27 | 13 | -0.13203 | 0.452312 | 0.655939 | 26.7339 | 26.79258 | 26.66054 |
| **P12388** | serpinb2 | ECM Regulators | Matrisome-associated | 13 | 40 | 13 | 0.216906 | 0.452419 | 0.655939 | 24.598 | 24.50159 | 24.7185 |
| **P47876** | igfbp1 | ECM Glycoproteins | Core matrisome | 9 | 18 | 9 | -0.22007 | 0.452464 | 0.655939 | 25.05395 | 25.15176 | 24.93169 |
| **Q9JHH5** | cxcl11 | Secreted Factors | Matrisome-associated | 6 | 6 | 6 | 0.347228 | 0.454423 | 0.656057 | 23.36803 | 23.21371 | 23.56094 |
| **Q2TJ95** | rspo3 | ECM Glycoproteins | Core matrisome | 12 | 39 | 12 | 0.164704 | 0.454538 | 0.656057 | 26.94404 | 26.87084 | 27.03555 |
| **Q8BRU4** | clec9a | ECM-affiliated Proteins | Matrisome-associated | 4 | 7 | 4 | 0.173057 | 0.455135 | 0.656057 | 25.84561 | 25.7687 | 25.94175 |
| **P37889** | fbln2 | ECM Glycoproteins | Core matrisome | 27 | 39 | 27 | -0.12915 | 0.456368 | 0.656057 | 26.94656 | 27.00396 | 26.87481 |
| **Q504P2** | clec12a | ECM-affiliated Proteins | Matrisome-associated | 8 | 13 | 8 | 0.262328 | 0.456718 | 0.656057 | 25.41134 | 25.29475 | 25.55708 |
| **P59509** | adamts19 | ECM Regulators | Matrisome-associated | 37 | 73 | 37 | -0.10464 | 0.457048 | 0.656057 | 27.22801 | 27.27452 | 27.16988 |
| **Q9Z0L4** | bmp15 | Secreted Factors | Matrisome-associated | 11 | 22 | 11 | -0.13545 | 0.457232 | 0.656057 | 25.58631 | 25.64651 | 25.51106 |
| **Q06770** | serpina6 | ECM Regulators | Matrisome-associated | 14 | 25 | 14 | 0.18587 | 0.460232 | 0.659397 | 25.44214 | 25.35954 | 25.5454 |
| **P62960** | ybx1 | Secreted Factors | Matrisome-associated | 14 | 37 | 12 | 0.223779 | 0.463227 | 0.662718 | 25.07303 | 24.97357 | 25.19735 |
| **P27090** | tgfb2 | Secreted Factors | Matrisome-associated | 18 | 26 | 17 | -0.16562 | 0.464946 | 0.664209 | 27.01582 | 27.08943 | 26.92381 |
| **O35188** | cx3cl1 | Secreted Factors | Matrisome-associated | 9 | 13 | 9 | 0.140208 | 0.46589 | 0.664588 | 26.48345 | 26.42114 | 26.56134 |
| **Q60718** | adam2 | ECM Regulators | Matrisome-associated | 21 | 25 | 21 | -0.15716 | 0.466831 | 0.664963 | 25.01861 | 25.08846 | 24.9313 |
| **P31955** | areg | Secreted Factors | Matrisome-associated | 5 | 7 | 5 | 0.160401 | 0.470081 | 0.665757 | 26.3938 | 26.32251 | 26.48291 |
| **Q3MI99** | ccbe1 | Secreted Factors | Matrisome-associated | 20 | 46 | 20 | 0.186978 | 0.470095 | 0.665757 | 28.67031 | 28.5872 | 28.77418 |
| **Q9QXT6** | il17b | Secreted Factors | Matrisome-associated | 17 | 39 | 17 | -0.2718 | 0.470125 | 0.665757 | 26.47643 | 26.59724 | 26.32543 |
| **P06797** | ctsl | ECM Regulators | Matrisome-associated | 14 | 30 | 14 | -0.17477 | 0.470464 | 0.665757 | 25.14411 | 25.22179 | 25.04702 |
| **Q05910** | adam8 | ECM Regulators | Matrisome-associated | 31 | 50 | 31 | -0.17276 | 0.470785 | 0.665757 | 27.7156 | 27.79238 | 27.61962 |
| **Q8CFR0** | c1ql2 | ECM-affiliated Proteins | Matrisome-associated | 13 | 44 | 10 | 0.243447 | 0.472294 | 0.666928 | 26.90295 | 26.79476 | 27.0382 |
| **Q9R045** | angptl2 | Secreted Factors | Matrisome-associated | 23 | 34 | 22 | 0.15859 | 0.473156 | 0.667184 | 26.90946 | 26.83898 | 26.99757 |
| **Q62401** | ccl12 | Secreted Factors | Matrisome-associated | 4 | 9 | 4 | -0.35675 | 0.475072 | 0.668924 | 24.95275 | 25.1113 | 24.75455 |
| **P35174** | stfa2 | ECM Regulators | Matrisome-associated | 4 | 4 | 4 | -0.33085 | 0.476806 | 0.670402 | 20.29666 | 20.4437 | 20.11285 |
| **Q924C6** | loxl4 | ECM Regulators | Matrisome-associated | 31 | 65 | 30 | -0.10125 | 0.478972 | 0.671329 | 27.97828 | 28.02328 | 27.92202 |
| **P40226** | thpo | Secreted Factors | Matrisome-associated | 15 | 25 | 15 | -0.20557 | 0.479058 | 0.671329 | 24.64999 | 24.74136 | 24.53579 |
| **P07350** | ifna6 | Secreted Factors | Matrisome-associated | 10 | 18 | 4 | -0.14982 | 0.479712 | 0.671329 | 27.33287 | 27.39946 | 27.24964 |
| **P83714** | ctf2 | Secreted Factors | Matrisome-associated | 1 | 4 | 1 | -0.15342 | 0.480206 | 0.671329 | 22.49222 | 22.56041 | 22.40699 |
| **O88676** | mmp23 | ECM Regulators | Matrisome-associated | 10 | 14 | 10 | 0.320468 | 0.481527 | 0.672217 | 23.41226 | 23.26983 | 23.59029 |
| **P50404** | sftpd | ECM-affiliated Proteins | Matrisome-associated | 41 | 110 | 41 | -0.11263 | 0.482718 | 0.672921 | 31.06088 | 31.11094 | 30.99831 |
| **Q9WUH7** | sema4g | ECM-affiliated Proteins | Matrisome-associated | 37 | 104 | 37 | 0.110466 | 0.483556 | 0.673132 | 28.75861 | 28.70952 | 28.81998 |
| **Q8BK62** | olfml3 | Secreted Factors | Matrisome-associated | 18 | 19 | 18 | -0.13091 | 0.484298 | 0.673209 | 26.03267 | 26.09085 | 25.95994 |
| **O35674** | adam19 | ECM Regulators | Matrisome-associated | 36 | 96 | 36 | 0.107069 | 0.488915 | 0.678663 | 27.84758 | 27.8 | 27.90706 |
| **Q70UZ7** | vwa2 | ECM Glycoproteins | Core matrisome | 33 | 58 | 33 | 0.346614 | 0.490425 | 0.679797 | 28.02809 | 27.87404 | 28.22065 |
| **Q62288** | spock1 | Proteoglycans | Core matrisome | 15 | 29 | 15 | -0.17522 | 0.49179 | 0.680726 | 25.87727 | 25.95515 | 25.77993 |
| **P97766** | cfc1 | Secreted Factors | Matrisome-associated | 5 | 7 | 5 | -0.56332 | 0.493128 | 0.681197 | 19.57111 | 19.82147 | 19.25816 |
| **Q3TTE0** | adam5 | ECM Regulators | Matrisome-associated | 32 | 61 | 32 | 0.177297 | 0.49352 | 0.681197 | 26.89698 | 26.81818 | 26.99548 |
| **Q8R2Z5** | vwa1 | ECM Glycoproteins | Core matrisome | 8 | 13 | 8 | 0.261101 | 0.496111 | 0.68381 | 25.3229 | 25.20686 | 25.46796 |
| **Q8CAL5** | gpc5 | ECM-affiliated Proteins | Matrisome-associated | 24 | 33 | 24 | 0.121458 | 0.504121 | 0.693549 | 27.60848 | 27.55449 | 27.67595 |
| **O70460** | ccl19 | Secreted Factors | Matrisome-associated | 4 | 5 | 4 | -0.33363 | 0.505087 | 0.693549 | 23.29747 | 23.44576 | 23.11212 |
| **Q4ZJN1** | c1qtnf9 | ECM-affiliated Proteins | Matrisome-associated | 31 | 106 | 31 | -0.11739 | 0.505998 | 0.693549 | 29.6274 | 29.67957 | 29.56218 |
| **Q9EPX2** | papln | ECM Glycoproteins | Core matrisome | 57 | 108 | 57 | 0.138581 | 0.506008 | 0.693549 | 29.95402 | 29.89242 | 30.031 |
| **P63089** | ptn | Secreted Factors | Matrisome-associated | 7 | 10 | 7 | -0.39559 | 0.507082 | 0.694051 | 23.58422 | 23.76003 | 23.36445 |
| **Q80WM4** | hapln4 | Proteoglycans | Core matrisome | 25 | 48 | 25 | -0.16379 | 0.508473 | 0.694984 | 26.62454 | 26.69734 | 26.53355 |
| **O35256** | prl4a1 | Secreted Factors | Matrisome-associated | 9 | 15 | 9 | 0.357964 | 0.514994 | 0.702248 | 23.23218 | 23.07309 | 23.43105 |
| **O88430** | ccl22 | Secreted Factors | Matrisome-associated | 1 | 1 | 1 | -0.382 | 0.51522 | 0.702248 | 24.34923 | 24.51901 | 24.13701 |
| **P07141** | csf1 | Secreted Factors | Matrisome-associated | 21 | 51 | 21 | -0.2025 | 0.51927 | 0.706204 | 27.63409 | 27.72409 | 27.52159 |
| **Q60675** | lama2 | ECM Glycoproteins | Core matrisome | 131 | 262 | 131 | 0.082882 | 0.519564 | 0.706204 | 30.86592 | 30.82908 | 30.91196 |
| **Q9DBB9** | cpn2 | ECM Regulators | Matrisome-associated | 18 | 30 | 18 | -0.0966 | 0.522823 | 0.709185 | 25.86818 | 25.91111 | 25.81451 |
| **Q00731** | vegfa | Secreted Factors | Matrisome-associated | 6 | 12 | 6 | -0.18611 | 0.523205 | 0.709185 | 24.67663 | 24.75935 | 24.57324 |
| **P06799** | ifna7 | Secreted Factors | Matrisome-associated | 4 | 10 | 2 | 0.126304 | 0.526517 | 0.712689 | 28.54756 | 28.49142 | 28.61773 |
| **Q07456** | ambp | ECM Regulators | Matrisome-associated | 12 | 20 | 12 | -0.41211 | 0.527866 | 0.71353 | 24.657 | 24.84016 | 24.42805 |
| **P18121** | prl3d1 | Secreted Factors | Matrisome-associated | 3 | 7 | 3 | -0.27875 | 0.530721 | 0.7164 | 24.32384 | 24.44773 | 24.16898 |
| **P12246** | apcs | Secreted Factors | Matrisome-associated | 2 | 5 | 2 | -0.24253 | 0.5319 | 0.717004 | 22.12649 | 22.23428 | 21.99175 |
| **O09037** | reg3a | ECM-affiliated Proteins | Matrisome-associated | 2 | 3 | 2 | 0.202368 | 0.534415 | 0.719157 | 23.2323 | 23.14236 | 23.34473 |
| **Q9QXT5** | egfl7 | Secreted Factors | Matrisome-associated | 13 | 20 | 13 | 0.110706 | 0.535192 | 0.719157 | 26.44906 | 26.39986 | 26.51057 |
| **O35257** | prl6a1 | Secreted Factors | Matrisome-associated | 7 | 14 | 7 | -0.10863 | 0.536748 | 0.719157 | 25.1986 | 25.24688 | 25.13825 |
| **Q9Z1N6** | sfrp4 | Secreted Factors | Matrisome-associated | 16 | 23 | 16 | 0.112628 | 0.536874 | 0.719157 | 26.30491 | 26.25485 | 26.36748 |
| **Q30D77** | col24a1 | Collagens | Core matrisome | 140 | 442 | 140 | -0.0578 | 0.537167 | 0.719157 | 31.13721 | 31.16289 | 31.1051 |
| **P57748** | mmp20 | ECM Regulators | Matrisome-associated | 24 | 40 | 24 | 0.209976 | 0.54065 | 0.722834 | 26.25223 | 26.15891 | 26.36888 |
| **P01572** | ifna1 | Secreted Factors | Matrisome-associated | 13 | 18 | 3 | -0.25804 | 0.544248 | 0.726652 | 24.25476 | 24.36945 | 24.1114 |
| **Q9CQ04** | ogfod2 | ECM Regulators | Matrisome-associated | 6 | 11 | 6 | 0.243434 | 0.54535 | 0.727134 | 25.20969 | 25.10149 | 25.34493 |
| **Q9R1B9** | slit2 | ECM Glycoproteins | Core matrisome | 97 | 213 | 95 | 0.059279 | 0.548704 | 0.729664 | 28.15662 | 28.13028 | 28.18956 |
| **P28047** | wnt7b | Secreted Factors | Matrisome-associated | 20 | 41 | 17 | 0.152095 | 0.550906 | 0.729664 | 27.82233 | 27.75474 | 27.90683 |
| **O35608** | angpt2 | Secreted Factors | Matrisome-associated | 18 | 99 | 18 | -0.19891 | 0.552025 | 0.729664 | 27.72433 | 27.81274 | 27.61383 |
| **P50543** | s100a11 | Secreted Factors | Matrisome-associated | 7 | 8 | 7 | 0.266584 | 0.552731 | 0.729664 | 24.96698 | 24.8485 | 25.11508 |
| **Q8BKV1** | gpc2 | ECM-affiliated Proteins | Matrisome-associated | 32 | 59 | 32 | 0.103776 | 0.552985 | 0.729664 | 28.18934 | 28.14321 | 28.24699 |
| **P26011** | itgb7 | ECM-affiliated Proteins | Matrisome-associated | 31 | 58 | 31 | 0.05805 | 0.553562 | 0.729664 | 27.21114 | 27.18534 | 27.24339 |
| **Q05928** | btc | Secreted Factors | Matrisome-associated | 5 | 14 | 5 | -0.2576 | 0.553677 | 0.729664 | 24.14065 | 24.25514 | 23.99754 |
| **Q9QZS0** | col4a3 | Collagens | Core matrisome | 135 | 430 | 135 | 0.114748 | 0.553795 | 0.729664 | 31.38555 | 31.33455 | 31.44929 |
| **Q9R1V4** | adam11 | ECM Regulators | Matrisome-associated | 27 | 63 | 27 | -0.09782 | 0.553949 | 0.729664 | 27.1098 | 27.15327 | 27.05545 |
| **O35348** | colq | ECM Glycoproteins | Core matrisome | 25 | 56 | 25 | 0.130663 | 0.555179 | 0.730303 | 27.0673 | 27.00923 | 27.13989 |
| **P97399** | dspp | ECM Glycoproteins | Core matrisome | 17 | 34 | 17 | 0.102537 | 0.557852 | 0.732835 | 27.19953 | 27.15396 | 27.2565 |
| **P05017** | igf1 | Secreted Factors | Matrisome-associated | 4 | 5 | 4 | 0.168226 | 0.560405 | 0.735203 | 22.43464 | 22.35988 | 22.5281 |
| **P18828** | sdc1 | ECM-affiliated Proteins | Matrisome-associated | 7 | 31 | 7 | 0.129926 | 0.566419 | 0.7421 | 28.24983 | 28.19208 | 28.32201 |
| **Q9JHG0** | cbln3 | Secreted Factors | Matrisome-associated | 8 | 18 | 8 | -0.32757 | 0.56863 | 0.744002 | 26.55117 | 26.69676 | 26.36919 |
| **Q61738** | itga7 | ECM-affiliated Proteins | Matrisome-associated | 37 | 94 | 35 | -0.07061 | 0.570888 | 0.745653 | 27.42545 | 27.45684 | 27.38623 |
| **Q61247** | serpinf2 | ECM Regulators | Matrisome-associated | 19 | 43 | 19 | 0.111941 | 0.571413 | 0.745653 | 26.27797 | 26.22822 | 26.34016 |
| **Q80ZF2** | ifne | Secreted Factors | Matrisome-associated | 6 | 15 | 6 | -0.13242 | 0.572813 | 0.746142 | 26.21401 | 26.27286 | 26.14044 |
| **P17515** | cxcl10 | Secreted Factors | Matrisome-associated | 4 | 6 | 4 | 0.21526 | 0.573311 | 0.746142 | 24.37474 | 24.27907 | 24.49433 |
| **A2AVA0** | svep1 | ECM Glycoproteins | Core matrisome | 82 | 164 | 82 | 0.065565 | 0.574382 | 0.746544 | 28.9681 | 28.93896 | 29.00453 |
| **P97384** | anxa11 | ECM-affiliated Proteins | Matrisome-associated | 9 | 10 | 9 | 0.454136 | 0.575565 | 0.746925 | 25.11029 | 24.90845 | 25.36258 |
| **Q9WVB4** | slit3 | ECM Glycoproteins | Core matrisome | 62 | 124 | 62 | -0.35302 | 0.576199 | 0.746925 | 29.67904 | 29.83594 | 29.48292 |
| **Q80WM5** | hapln3 | Proteoglycans | Core matrisome | 14 | 22 | 14 | 0.286739 | 0.577398 | 0.74749 | 24.3472 | 24.21976 | 24.5065 |
| **Q64519** | sdc3 | ECM-affiliated Proteins | Matrisome-associated | 14 | 20 | 14 | -0.55954 | 0.583152 | 0.753943 | 24.2282 | 24.47688 | 23.91735 |
| **Q9QZC2** | plxnc1 | ECM-affiliated Proteins | Matrisome-associated | 60 | 83 | 60 | -0.08871 | 0.58638 | 0.755533 | 29.93298 | 29.97241 | 29.8837 |
| **Q8BWY2** | clec1a | ECM-affiliated Proteins | Matrisome-associated | 4 | 7 | 4 | -0.16781 | 0.586412 | 0.755533 | 23.65387 | 23.72846 | 23.56064 |
| **Q9R159** | adam25 | ECM Regulators | Matrisome-associated | 21 | 48 | 21 | 0.151246 | 0.586694 | 0.755533 | 25.7592 | 25.69198 | 25.84322 |
| **P54320** | eln | ECM Glycoproteins | Core matrisome | 17 | 34 | 17 | 0.099083 | 0.588906 | 0.757069 | 26.69961 | 26.65558 | 26.75466 |
| **O89101** | fgf18 | Secreted Factors | Matrisome-associated | 9 | 18 | 9 | -0.22436 | 0.589433 | 0.757069 | 26.93752 | 27.03723 | 26.81287 |
| **Q9Z0E2** | chrd | Secreted Factors | Matrisome-associated | 39 | 79 | 39 | 0.091492 | 0.590544 | 0.757504 | 27.27454 | 27.23388 | 27.32537 |
| **P51885** | lum | Proteoglycans | Core matrisome | 20 | 41 | 20 | 0.155831 | 0.592454 | 0.758435 | 25.4238 | 25.35454 | 25.51037 |
| **Q9JI78** | ngly1 | ECM Regulators | Matrisome-associated | 32 | 49 | 32 | -0.09105 | 0.592818 | 0.758435 | 28.85042 | 28.89088 | 28.79983 |
| **Q7TQN3** | wfikkn2 | Secreted Factors | Matrisome-associated | 11 | 20 | 10 | -0.26637 | 0.598062 | 0.763623 | 25.67664 | 25.79503 | 25.52866 |
| **Q02853** | mmp11 | ECM Regulators | Matrisome-associated | 13 | 35 | 13 | -0.16486 | 0.598431 | 0.763623 | 25.58899 | 25.66226 | 25.4974 |
| **Q9JHB3** | timp4 | ECM Regulators | Matrisome-associated | 3 | 14 | 3 | -0.09563 | 0.59926 | 0.763686 | 25.906 | 25.9485 | 25.85287 |
| **P41317** | mbl2 | ECM-affiliated Proteins | Matrisome-associated | 11 | 38 | 9 | 0.133997 | 0.606179 | 0.7715 | 27.55824 | 27.49869 | 27.63269 |
| **O35622** | fgf15 | Secreted Factors | Matrisome-associated | 7 | 9 | 7 | 0.096223 | 0.607487 | 0.772162 | 26.9407 | 26.89793 | 26.99415 |
| **Q80TR4** | slit1 | ECM Glycoproteins | Core matrisome | 49 | 93 | 47 | -0.07237 | 0.610188 | 0.773664 | 26.63953 | 26.6717 | 26.59932 |
| **O70309** | itgb5 | ECM-affiliated Proteins | Matrisome-associated | 30 | 54 | 30 | -0.09814 | 0.611749 | 0.773664 | 27.06448 | 27.1081 | 27.00996 |
| **Q8R1W8** | impg1 | Proteoglycans | Core matrisome | 29 | 46 | 29 | -0.07183 | 0.612431 | 0.773664 | 27.71705 | 27.74897 | 27.67714 |
| **P20109** | il13 | Secreted Factors | Matrisome-associated | 5 | 10 | 5 | -0.14737 | 0.612566 | 0.773664 | 24.07425 | 24.13975 | 23.99238 |
| **Q8R1Q3** | angptl7 | Secreted Factors | Matrisome-associated | 16 | 38 | 16 | -0.11002 | 0.61293 | 0.773664 | 29.52006 | 29.56896 | 29.45894 |
| **P33622** | apoc3 | ECM-affiliated Proteins | Matrisome-associated | 2 | 2 | 2 | -0.1526 | 0.613405 | 0.773664 | 21.61506 | 21.68289 | 21.53029 |
| **Q8CDC0** | serpinb13 | ECM Regulators | Matrisome-associated | 10 | 11 | 10 | -0.16215 | 0.622522 | 0.784154 | 23.60171 | 23.67377 | 23.51162 |
| **Q9QZZ6** | dpt | ECM Glycoproteins | Core matrisome | 5 | 13 | 5 | 0.267381 | 0.624331 | 0.784561 | 23.15157 | 23.03274 | 23.30012 |
| **Q8BPB5** | efemp1 | ECM Glycoproteins | Core matrisome | 20 | 79 | 20 | -0.10136 | 0.624447 | 0.784561 | 26.50949 | 26.55455 | 26.45318 |
| **Q9EPL5** | mmp1a | ECM Regulators | Matrisome-associated | 15 | 24 | 9 | 0.157062 | 0.63393 | 0.792759 | 25.26281 | 25.193 | 25.35006 |
| **Q9Z175** | loxl3 | ECM Regulators | Matrisome-associated | 23 | 38 | 23 | 0.084097 | 0.634188 | 0.792759 | 27.75735 | 27.71997 | 27.80407 |
| **Q70E20** | sned1 | ECM Glycoproteins | Core matrisome | 36 | 63 | 36 | 0.082115 | 0.634999 | 0.792759 | 26.65974 | 26.62324 | 26.70536 |
| **Q8K410** | adam32 | ECM Regulators | Matrisome-associated | 27 | 43 | 27 | 0.191861 | 0.635513 | 0.792759 | 25.0506 | 24.96532 | 25.15719 |
| **O35367** | kera | Proteoglycans | Core matrisome | 23 | 78 | 23 | -0.07765 | 0.635571 | 0.792759 | 28.43777 | 28.47228 | 28.39464 |
| **Q8BZQ2** | crispld2 | ECM Glycoproteins | Core matrisome | 12 | 32 | 12 | 0.111835 | 0.635825 | 0.792759 | 26.02028 | 25.97058 | 26.08241 |
| **O88200** | clec11a | ECM-affiliated Proteins | Matrisome-associated | 12 | 28 | 12 | -0.11389 | 0.637673 | 0.793619 | 25.54913 | 25.59975 | 25.48586 |
| **Q6W3F0** | p4ha3 | ECM Regulators | Matrisome-associated | 17 | 28 | 17 | 0.19398 | 0.638134 | 0.793619 | 27.04103 | 26.95481 | 27.14879 |
| **Q922T2** | mfap3 | ECM Glycoproteins | Core matrisome | 4 | 18 | 4 | -0.19387 | 0.646293 | 0.800188 | 26.17334 | 26.2595 | 26.06563 |
| **P53690** | mmp14 | ECM Regulators | Matrisome-associated | 17 | 48 | 17 | -0.05986 | 0.64658 | 0.800188 | 27.97745 | 28.00406 | 27.9442 |
| **Q2VIS4** | flg2 | Secreted Factors | Matrisome-associated | 62 | 113 | 62 | -0.04929 | 0.646593 | 0.800188 | 28.00268 | 28.02459 | 27.9753 |
| **Q8R534** | adam1b | ECM Regulators | Matrisome-associated | 25 | 39 | 16 | -0.21784 | 0.646682 | 0.800188 | 23.99199 | 24.0888 | 23.87096 |
| **Q9WVM6** | tll2 | ECM Regulators | Matrisome-associated | 38 | 116 | 34 | 0.062736 | 0.649654 | 0.802851 | 28.68025 | 28.65236 | 28.7151 |
| **Q07079** | igfbp5 | ECM Glycoproteins | Core matrisome | 7 | 12 | 7 | 0.104829 | 0.65522 | 0.808709 | 24.29795 | 24.25136 | 24.35619 |
| **Q9D1D6** | cthrc1 | ECM Glycoproteins | Core matrisome | 13 | 30 | 13 | 0.065242 | 0.656989 | 0.809873 | 27.2724 | 27.24341 | 27.30865 |
| **O08524** | tectb | ECM Glycoproteins | Core matrisome | 10 | 27 | 10 | -0.24433 | 0.660001 | 0.810106 | 23.65916 | 23.76775 | 23.52343 |
| **Q8CFZ4** | gpc3 | ECM-affiliated Proteins | Matrisome-associated | 19 | 28 | 19 | -0.09781 | 0.660141 | 0.810106 | 27.19642 | 27.2399 | 27.14208 |
| **P50114** | s100b | Secreted Factors | Matrisome-associated | 5 | 11 | 5 | -0.23327 | 0.660287 | 0.810106 | 27.0005 | 27.10418 | 26.8709 |
| **P22724** | wnt4 | Secreted Factors | Matrisome-associated | 15 | 19 | 15 | -0.12856 | 0.661057 | 0.810106 | 26.47501 | 26.53215 | 26.40359 |
| **P55002** | mfap2 | ECM Glycoproteins | Core matrisome | 5 | 7 | 5 | -0.08604 | 0.662017 | 0.810106 | 22.8649 | 22.90315 | 22.8171 |
| **Q91ZX1** | cd209a | ECM-affiliated Proteins | Matrisome-associated | 8 | 17 | 8 | 0.22573 | 0.662137 | 0.810106 | 23.43488 | 23.33455 | 23.56028 |
| **Q9D695** | serpinb7 | ECM Regulators | Matrisome-associated | 22 | 37 | 22 | 0.094183 | 0.666845 | 0.813452 | 27.08107 | 27.03921 | 27.1334 |
| **Q8BYI9** | tnr | ECM Glycoproteins | Core matrisome | 30 | 52 | 30 | -0.06555 | 0.667063 | 0.813452 | 27.36393 | 27.39306 | 27.32751 |
| **Q8R422** | cd109 | ECM Regulators | Matrisome-associated | 40 | 61 | 40 | -0.07237 | 0.667363 | 0.813452 | 27.83503 | 27.86719 | 27.79482 |
| **Q61292** | lamb2 | ECM Glycoproteins | Core matrisome | 82 | 159 | 81 | -0.08356 | 0.67039 | 0.816126 | 29.10543 | 29.14257 | 29.05901 |
| **Q8K007** | sulf1 | ECM Regulators | Matrisome-associated | 28 | 116 | 28 | -0.13632 | 0.672189 | 0.817301 | 25.98086 | 26.04145 | 25.90513 |
| **P16294** | f9 | ECM Regulators | Matrisome-associated | 11 | 20 | 11 | -0.09442 | 0.676438 | 0.821448 | 26.15314 | 26.1951 | 26.10068 |
| **P43029** | gdf7 | Secreted Factors | Matrisome-associated | 33 | 129 | 33 | 0.07564 | 0.679191 | 0.823768 | 29.29818 | 29.26457 | 29.34021 |
| **F8VQ03** | adam3 | ECM Regulators | Matrisome-associated | 16 | 21 | 16 | -0.15328 | 0.680787 | 0.823768 | 24.8282 | 24.89632 | 24.74304 |
| **Q68SA9** | adamts7 | ECM Regulators | Matrisome-associated | 42 | 91 | 42 | -0.05378 | 0.682234 | 0.823768 | 28.18897 | 28.21287 | 28.1591 |
| **Q4VBE4** | egflam | ECM Glycoproteins | Core matrisome | 34 | 53 | 34 | -0.06653 | 0.682858 | 0.823768 | 25.70408 | 25.73365 | 25.66712 |
| **Q9ESL8** | fgf16 | Secreted Factors | Matrisome-associated | 3 | 6 | 3 | 0.082739 | 0.684135 | 0.823768 | 25.52555 | 25.48878 | 25.57152 |
| **P57785** | lefty2 | Secreted Factors | Matrisome-associated | 13 | 16 | 7 | -0.32777 | 0.684348 | 0.823768 | 24.68434 | 24.83001 | 24.50224 |
| **P20863** | gdf1 | Secreted Factors | Matrisome-associated | 13 | 40 | 13 | -0.05377 | 0.684589 | 0.823768 | 26.93794 | 26.96184 | 26.90807 |
| **P01325** | ins1 | Secreted Factors | Matrisome-associated | 2 | 3 | 2 | 0.066492 | 0.685072 | 0.823768 | 23.67917 | 23.64962 | 23.71611 |
| **Q80SU4** | ifna13 | Secreted Factors | Matrisome-associated | 9 | 15 | 5 | 0.094886 | 0.686251 | 0.824174 | 27.29082 | 27.24865 | 27.34353 |
| **P04768** | prl2c3 | Secreted Factors | Matrisome-associated | 19 | 49 | 2 | -0.06099 | 0.690099 | 0.827781 | 27.63582 | 27.66293 | 27.60194 |
| **Q9WTR0** | mmp16 | ECM Regulators | Matrisome-associated | 12 | 41 | 12 | 0.111751 | 0.695397 | 0.833117 | 26.79564 | 26.74597 | 26.85772 |
| **Q9R171** | cbln1 | Secreted Factors | Matrisome-associated | 12 | 20 | 9 | -0.40241 | 0.697173 | 0.834224 | 26.47761 | 26.65646 | 26.25405 |
| **Q08731** | reg2 | ECM-affiliated Proteins | Matrisome-associated | 8 | 27 | 8 | -0.08971 | 0.705208 | 0.842809 | 25.47588 | 25.51575 | 25.42604 |
| **Q61704** | itih3 | ECM Regulators | Matrisome-associated | 23 | 60 | 23 | -0.10964 | 0.708745 | 0.846004 | 28.60704 | 28.65577 | 28.54613 |
| **Q9Z123** | sema4f | ECM-affiliated Proteins | Matrisome-associated | 21 | 55 | 21 | -0.06505 | 0.71023 | 0.84617 | 27.5355 | 27.56441 | 27.49937 |
| **Q6QLQ4** | clec7a | ECM-affiliated Proteins | Matrisome-associated | 11 | 15 | 11 | 0.079265 | 0.71061 | 0.84617 | 25.52905 | 25.49382 | 25.57309 |
| **Q62005** | zp1 | ECM Glycoproteins | Core matrisome | 15 | 18 | 15 | 0.124497 | 0.713718 | 0.846663 | 25.5583 | 25.50296 | 25.62746 |
| **Q8BMF8** | gldn | ECM Glycoproteins | Core matrisome | 40 | 120 | 40 | 0.036921 | 0.713941 | 0.846663 | 28.40718 | 28.39077 | 28.42769 |
| **Q8CIE0** | serpina11 | ECM Regulators | Matrisome-associated | 5 | 5 | 5 | 0.114564 | 0.71433 | 0.846663 | 23.11888 | 23.06796 | 23.18253 |
| **Q9JM99** | prg4 | Proteoglycans | Core matrisome | 55 | 103 | 55 | -0.10873 | 0.71448 | 0.846663 | 28.45502 | 28.50335 | 28.39462 |
| **Q62356** | fstl1 | Secreted Factors | Matrisome-associated | 17 | 30 | 17 | -0.0628 | 0.720788 | 0.852259 | 26.90997 | 26.93788 | 26.87508 |
| **P12032** | timp1 | ECM Regulators | Matrisome-associated | 13 | 19 | 13 | 0.071698 | 0.721041 | 0.852259 | 30.15929 | 30.12743 | 30.19913 |
| **Q8R0Z6** | angptl6 | Secreted Factors | Matrisome-associated | 23 | 46 | 23 | 0.055968 | 0.721811 | 0.852259 | 27.65795 | 27.63308 | 27.68904 |
| **Q61716** | ifna11 | Secreted Factors | Matrisome-associated | 7 | 14 | 2 | -0.13345 | 0.723873 | 0.852385 | 21.3196 | 21.37891 | 21.24546 |
| **Q9D0F3** | lman1 | ECM-affiliated Proteins | Matrisome-associated | 19 | 29 | 19 | -0.08208 | 0.725035 | 0.852385 | 25.6334 | 25.66988 | 25.5878 |
| **Q2VWQ2** | nell1 | ECM Glycoproteins | Core matrisome | 21 | 44 | 21 | 0.331983 | 0.72527 | 0.852385 | 28.52176 | 28.37421 | 28.70619 |
| **Q9QYY7** | esm1 | Proteoglycans | Core matrisome | 7 | 11 | 7 | -0.11353 | 0.726003 | 0.852385 | 23.85796 | 23.90842 | 23.79489 |
| **Q64151** | sema4c | ECM-affiliated Proteins | Matrisome-associated | 30 | 70 | 30 | 0.091606 | 0.726267 | 0.852385 | 26.86064 | 26.81992 | 26.91153 |
| **P97857** | adamts1 | ECM Regulators | Matrisome-associated | 34 | 64 | 34 | -0.03979 | 0.730603 | 0.854439 | 28.66496 | 28.68265 | 28.64286 |
| **Q8C6K9** | col6a6 | Collagens | Core matrisome | 109 | 240 | 98 | 0.032791 | 0.73089 | 0.854439 | 30.88915 | 30.87458 | 30.90737 |
| **P49300** | clec10a | ECM-affiliated Proteins | Matrisome-associated | 14 | 28 | 14 | 0.066974 | 0.731184 | 0.854439 | 27.22599 | 27.19622 | 27.26319 |
| **P55097** | ctsk | ECM Regulators | Matrisome-associated | 12 | 41 | 12 | 0.056448 | 0.731505 | 0.854439 | 25.84086 | 25.81578 | 25.87222 |
| **Q9R0Q8** | clec4e | ECM-affiliated Proteins | Matrisome-associated | 5 | 6 | 5 | 0.322037 | 0.733613 | 0.855882 | 19.86937 | 19.72624 | 20.04827 |
| **Q8R4W6** | pcolce2 | ECM Glycoproteins | Core matrisome | 22 | 34 | 22 | 0.064762 | 0.736423 | 0.858139 | 26.36458 | 26.3358 | 26.40056 |
| **Q91WP6** | serpina3n | ECM Regulators | Matrisome-associated | 12 | 21 | 7 | -0.14018 | 0.740986 | 0.860632 | 21.57326 | 21.63557 | 21.49539 |
| **Q60753** | ctf1 | Secreted Factors | Matrisome-associated | 13 | 26 | 13 | 0.132711 | 0.741666 | 0.860632 | 26.89884 | 26.83986 | 26.97257 |
| **P48036** | anxa5 | ECM-affiliated Proteins | Matrisome-associated | 17 | 71 | 17 | 0.061477 | 0.74172 | 0.860632 | 27.73101 | 27.70368 | 27.76516 |
| **O55188** | dmp1 | ECM Glycoproteins | Core matrisome | 9 | 17 | 9 | -0.22617 | 0.742075 | 0.860632 | 23.43546 | 23.53598 | 23.3098 |
| **Q04998** | inhba | Secreted Factors | Matrisome-associated | 20 | 45 | 20 | -0.11325 | 0.749698 | 0.868444 | 28.76097 | 28.8113 | 28.69805 |
| **Q9JHK0** | prl2a1 | Secreted Factors | Matrisome-associated | 7 | 12 | 7 | -0.1104 | 0.75132 | 0.869296 | 22.18206 | 22.23113 | 22.12073 |
| **Q6NVD0** | frem2 | ECM-affiliated Proteins | Matrisome-associated | 105 | 199 | 105 | 0.038419 | 0.753122 | 0.870353 | 29.91329 | 29.89622 | 29.93463 |
| **Q61703** | itih2 | ECM Regulators | Matrisome-associated | 38 | 94 | 38 | -0.04494 | 0.754045 | 0.870394 | 28.52821 | 28.54818 | 28.50325 |
| **P61329** | fgf12 | Secreted Factors | Matrisome-associated | 8 | 23 | 7 | -0.10109 | 0.756633 | 0.872353 | 24.75521 | 24.80014 | 24.69905 |
| **P98086** | c1qa | ECM-affiliated Proteins | Matrisome-associated | 16 | 55 | 16 | -0.05185 | 0.760741 | 0.876059 | 26.31746 | 26.34051 | 26.28865 |
| **O70514** | fgfbp1 | Secreted Factors | Matrisome-associated | 21 | 57 | 21 | 0.079959 | 0.761729 | 0.876167 | 28.21603 | 28.1805 | 28.26045 |
| **P43025** | clec3b | ECM-affiliated Proteins | Matrisome-associated | 7 | 10 | 7 | 0.103503 | 0.763145 | 0.876639 | 23.74829 | 23.70229 | 23.80579 |
| **Q62000** | ogn | Proteoglycans | Core matrisome | 19 | 49 | 19 | 0.057093 | 0.763929 | 0.876639 | 26.22663 | 26.20125 | 26.25835 |
| **P47880** | igfbp6 | ECM Glycoproteins | Core matrisome | 17 | 24 | 17 | 0.050315 | 0.766014 | 0.877772 | 28.0686 | 28.04624 | 28.09656 |
| **P97812** | ihh | Secreted Factors | Matrisome-associated | 23 | 42 | 21 | -0.05247 | 0.767049 | 0.877772 | 26.77144 | 26.79476 | 26.74229 |
| **P21237** | bdnf | Secreted Factors | Matrisome-associated | 14 | 21 | 14 | -0.06605 | 0.767603 | 0.877772 | 25.85764 | 25.887 | 25.82095 |
| **Q62178** | sema4a | ECM-affiliated Proteins | Matrisome-associated | 33 | 65 | 33 | -0.0446 | 0.769806 | 0.878272 | 27.90617 | 27.92599 | 27.88139 |
| **Q9JI76** | adam21 | ECM Regulators | Matrisome-associated | 19 | 25 | 19 | 0.047593 | 0.770485 | 0.878272 | 23.90374 | 23.88259 | 23.93018 |
| **Q5UBV8** | tnfsf15 | Secreted Factors | Matrisome-associated | 12 | 21 | 12 | 0.097725 | 0.770729 | 0.878272 | 27.24681 | 27.20337 | 27.3011 |
| **P50405** | sftpb | ECM-affiliated Proteins | Matrisome-associated | 4 | 30 | 4 | -0.07032 | 0.775325 | 0.881915 | 26.23925 | 26.27051 | 26.20018 |
| **P04202** | tgfb1 | Secreted Factors | Matrisome-associated | 20 | 31 | 19 | -0.14152 | 0.775725 | 0.881915 | 26.53344 | 26.59634 | 26.45482 |
| **Q6DFV8** | vwde | ECM Glycoproteins | Core matrisome | 22 | 41 | 22 | -0.06268 | 0.778134 | 0.883459 | 26.61042 | 26.63828 | 26.5756 |
| **P70275** | sema3e | ECM-affiliated Proteins | Matrisome-associated | 28 | 40 | 27 | 0.096412 | 0.779654 | 0.883459 | 26.99955 | 26.9567 | 27.05311 |
| **P15089** | cpa3 | ECM-affiliated Proteins | Matrisome-associated | 14 | 15 | 14 | 0.10821 | 0.779787 | 0.883459 | 24.95987 | 24.91178 | 25.01999 |
| **P32261** | serpinc1 | ECM Regulators | Matrisome-associated | 27 | 61 | 27 | -0.0967 | 0.786471 | 0.889183 | 29.39027 | 29.43325 | 29.33654 |
| **Q8BJ66** | kazald1 | ECM Regulators | Matrisome-associated | 13 | 16 | 13 | 0.090556 | 0.786654 | 0.889183 | 26.89345 | 26.8532 | 26.94376 |
| **Q64280** | lefty1 | Secreted Factors | Matrisome-associated | 13 | 16 | 7 | 0.172671 | 0.788711 | 0.890481 | 23.73833 | 23.66158 | 23.83426 |
| **Q8CJ69** | bmper | ECM Glycoproteins | Core matrisome | 19 | 37 | 19 | -0.04087 | 0.793101 | 0.893705 | 29.01563 | 29.0338 | 28.99293 |
| **Q62381** | tll1 | ECM Regulators | Matrisome-associated | 34 | 70 | 32 | -0.07037 | 0.793578 | 0.893705 | 27.50824 | 27.53952 | 27.46914 |
| **Q8CIZ8** | vwf | ECM Glycoproteins | Core matrisome | 81 | 148 | 81 | 0.046461 | 0.794303 | 0.893705 | 28.55576 | 28.53511 | 28.58157 |
| **P20239** | zp2 | ECM Glycoproteins | Core matrisome | 21 | 36 | 21 | -0.0597 | 0.797255 | 0.895998 | 26.48206 | 26.5086 | 26.4489 |
| **Q9WTX4** | nrg4 | Secreted Factors | Matrisome-associated | 6 | 12 | 6 | -0.10534 | 0.798855 | 0.896767 | 24.1354 | 24.18222 | 24.07688 |
| **Q9DAS4** | prl8a8 | Secreted Factors | Matrisome-associated | 7 | 12 | 6 | -0.11177 | 0.801288 | 0.897177 | 23.15362 | 23.20329 | 23.09153 |
| **P01139** | ngf | Secreted Factors | Matrisome-associated | 10 | 11 | 10 | 0.064566 | 0.801416 | 0.897177 | 28.12711 | 28.09841 | 28.16298 |
| **Q99MQ4** | aspn | Proteoglycans | Core matrisome | 25 | 49 | 25 | 0.053081 | 0.803239 | 0.897177 | 26.3832 | 26.35961 | 26.41269 |
| **Q9QUR8** | sema7a | ECM-affiliated Proteins | Matrisome-associated | 44 | 86 | 44 | 0.031385 | 0.803398 | 0.897177 | 27.88302 | 27.86907 | 27.90045 |
| **P11859** | agt | ECM Regulators | Matrisome-associated | 10 | 22 | 10 | -0.05116 | 0.804667 | 0.897177 | 25.97362 | 25.99636 | 25.9452 |
| **Q9R087** | gpc6 | ECM-affiliated Proteins | Matrisome-associated | 25 | 46 | 23 | 0.056705 | 0.804713 | 0.897177 | 27.31561 | 27.2904 | 27.34711 |
| **Q1HCM0** | fgfbp3 | Secreted Factors | Matrisome-associated | 18 | 46 | 17 | 0.042232 | 0.807322 | 0.899063 | 26.98143 | 26.96266 | 27.00489 |
| **P05208** | cela2a | ECM Regulators | Matrisome-associated | 9 | 16 | 9 | -0.54457 | 0.810688 | 0.901787 | 24.63923 | 24.88126 | 24.33669 |
| **O54693** | eda | Secreted Factors | Matrisome-associated | 9 | 24 | 9 | 0.096642 | 0.812405 | 0.902672 | 27.30113 | 27.25818 | 27.35482 |
| **P70124** | serpinb5 | ECM Regulators | Matrisome-associated | 21 | 26 | 21 | -0.07601 | 0.814879 | 0.904395 | 26.41719 | 26.45097 | 26.37496 |
| **Q08857** | cd36 | ECM-affiliated Proteins | Matrisome-associated | 15 | 18 | 15 | -0.10633 | 0.817133 | 0.905023 | 25.43124 | 25.4785 | 25.37217 |
| **Q9QUM0** | itga2b | ECM-affiliated Proteins | Matrisome-associated | 34 | 53 | 34 | -0.03531 | 0.818405 | 0.905023 | 28.17395 | 28.18964 | 28.15434 |
| **Q9QUN5** | prl3c1 | Secreted Factors | Matrisome-associated | 16 | 33 | 16 | -0.06103 | 0.818641 | 0.905023 | 28.29376 | 28.32089 | 28.25986 |
| **Q62165** | dag1 | ECM-affiliated Proteins | Matrisome-associated | 38 | 86 | 38 | -0.03286 | 0.819613 | 0.905023 | 28.65453 | 28.66913 | 28.63628 |
| **A2RT60** | htra4 | ECM Regulators | Matrisome-associated | 17 | 41 | 17 | 0.071916 | 0.820061 | 0.905023 | 26.72257 | 26.6906 | 26.76252 |
| **Q9QUP5** | hapln1 | Proteoglycans | Core matrisome | 13 | 24 | 13 | -0.12234 | 0.823789 | 0.908114 | 25.72702 | 25.78139 | 25.65905 |
| **O09049** | reg3g | ECM-affiliated Proteins | Matrisome-associated | 4 | 12 | 4 | -0.05597 | 0.828379 | 0.912148 | 24.3462 | 24.37107 | 24.31511 |
| **P11214** | plat | ECM Regulators | Matrisome-associated | 23 | 59 | 23 | -0.02912 | 0.830362 | 0.913305 | 26.09953 | 26.11247 | 26.08335 |
| **Q07105** | gdf9 | Secreted Factors | Matrisome-associated | 18 | 36 | 18 | -0.04059 | 0.832126 | 0.91377 | 27.85623 | 27.87427 | 27.83368 |
| **Q91VF5** | emid1 | ECM Glycoproteins | Core matrisome | 23 | 51 | 23 | -0.03523 | 0.832649 | 0.91377 | 27.3694 | 27.38506 | 27.34982 |
| **Q8BGU2** | cbln2 | Secreted Factors | Matrisome-associated | 9 | 30 | 7 | 0.077483 | 0.834721 | 0.915019 | 26.31154 | 26.2771 | 26.35458 |
| **Q91UZ4** | egln3 | ECM Regulators | Matrisome-associated | 10 | 18 | 10 | 0.058525 | 0.835949 | 0.915341 | 26.48238 | 26.45637 | 26.5149 |
| **P09225** | lta | Secreted Factors | Matrisome-associated | 7 | 8 | 7 | 0.036388 | 0.83816 | 0.916737 | 29.05223 | 29.03606 | 29.07245 |
| **P06728** | apoa4 | ECM-affiliated Proteins | Matrisome-associated | 27 | 77 | 27 | -0.04313 | 0.844014 | 0.922111 | 28.30619 | 28.32536 | 28.28223 |
| **Q61555** | fbn2 | ECM Glycoproteins | Core matrisome | 71 | 105 | 68 | 0.025618 | 0.846968 | 0.924308 | 28.81008 | 28.79869 | 28.82431 |
| **Q9WVJ9** | efemp2 | ECM Glycoproteins | Core matrisome | 9 | 22 | 9 | -0.04732 | 0.850245 | 0.926533 | 26.47344 | 26.49447 | 26.44715 |
| **O35228** | ebi3 | Secreted Factors | Matrisome-associated | 9 | 12 | 9 | 0.093418 | 0.850898 | 0.926533 | 22.95816 | 22.91664 | 23.01006 |
| **Q9Z0J7** | gdf15 | Secreted Factors | Matrisome-associated | 18 | 47 | 18 | -0.05568 | 0.852915 | 0.927699 | 27.02369 | 27.04843 | 26.99275 |
| **Q62469** | itga2 | ECM-affiliated Proteins | Matrisome-associated | 34 | 84 | 32 | -0.04264 | 0.85602 | 0.928012 | 27.78366 | 27.80262 | 27.75997 |
| **Q3UR50** | vwa5b2 | ECM Glycoproteins | Core matrisome | 33 | 79 | 33 | 0.112682 | 0.856848 | 0.928012 | 30.00789 | 29.95781 | 30.07049 |
| **Q9Z126** | pf4 | Secreted Factors | Matrisome-associated | 1 | 1 | 1 | 0.062336 | 0.857292 | 0.928012 | 23.78106 | 23.75336 | 23.81569 |
| **P50608** | fmod | Proteoglycans | Core matrisome | 26 | 61 | 26 | 0.034152 | 0.858181 | 0.928012 | 27.43749 | 27.42231 | 27.45646 |
| **Q9CYA0** | creld2 | ECM Glycoproteins | Core matrisome | 9 | 11 | 9 | 0.051263 | 0.858395 | 0.928012 | 25.9048 | 25.88201 | 25.93327 |
| **P43431** | il12a | Secreted Factors | Matrisome-associated | 6 | 12 | 6 | 0.143314 | 0.858884 | 0.928012 | 26.53784 | 26.47415 | 26.61746 |
| **Q8BJD1** | itih5 | ECM Regulators | Matrisome-associated | 35 | 59 | 35 | -0.03657 | 0.862971 | 0.9314 | 28.898 | 28.91425 | 28.87768 |
| **Q9D1U0** | grifin | ECM-affiliated Proteins | Matrisome-associated | 2 | 2 | 2 | 0.05124 | 0.865061 | 0.931642 | 21.00248 | 20.97971 | 21.03095 |
| **P28653** | bgn | Proteoglycans | Core matrisome | 30 | 106 | 30 | 0.035567 | 0.865607 | 0.931642 | 28.90846 | 28.89265 | 28.92822 |
| **P07349** | ifna5 | Secreted Factors | Matrisome-associated | 9 | 15 | 3 | 0.046734 | 0.866047 | 0.931642 | 23.55905 | 23.53828 | 23.58501 |
| **O35639** | anxa3 | ECM-affiliated Proteins | Matrisome-associated | 20 | 42 | 20 | -0.02515 | 0.867217 | 0.931878 | 27.0612 | 27.07237 | 27.04722 |
| **O70138** | mmp8 | ECM Regulators | Matrisome-associated | 22 | 45 | 22 | 0.034992 | 0.868792 | 0.932548 | 27.9988 | 27.98325 | 28.01824 |
| **O08573** | lgals9 | ECM-affiliated Proteins | Matrisome-associated | 9 | 16 | 9 | 0.059998 | 0.872538 | 0.935545 | 26.15932 | 26.13266 | 26.19265 |
| **Q9R1V6** | adam22 | ECM Regulators | Matrisome-associated | 44 | 85 | 44 | -0.02698 | 0.874817 | 0.93672 | 28.24226 | 28.25425 | 28.22727 |
| **P35441** | thbs1 | ECM Glycoproteins | Core matrisome | 70 | 173 | 70 | -0.01556 | 0.876978 | 0.93672 | 29.1899 | 29.19682 | 29.18126 |
| **Q64527** | wnt8a | Secreted Factors | Matrisome-associated | 20 | 37 | 20 | -0.02466 | 0.877148 | 0.93672 | 27.48084 | 27.4918 | 27.46714 |
| **P14069** | s100a6 | Secreted Factors | Matrisome-associated | 5 | 11 | 5 | -0.04927 | 0.877459 | 0.93672 | 26.14346 | 26.16535 | 26.11608 |
| **Q9JJY9** | il22 | Secreted Factors | Matrisome-associated | 8 | 10 | 3 | 0.046725 | 0.880488 | 0.938932 | 25.54396 | 25.5232 | 25.56992 |
| **Q8R4K8** | pappa | ECM Regulators | Matrisome-associated | 49 | 133 | 49 | 0.023173 | 0.881837 | 0.939348 | 29.6448 | 29.6345 | 29.65767 |
| **Q8CBR6** | tsku | ECM Glycoproteins | Core matrisome | 9 | 18 | 9 | 0.04154 | 0.885005 | 0.939923 | 24.37719 | 24.35873 | 24.40027 |
| **P07321** | epo | Secreted Factors | Matrisome-associated | 7 | 23 | 7 | -0.03242 | 0.88501 | 0.939923 | 26.00809 | 26.0225 | 25.99008 |
| **Q9R0B9** | plod2 | ECM Regulators | Matrisome-associated | 36 | 45 | 36 | -0.02208 | 0.885254 | 0.939923 | 27.25031 | 27.26012 | 27.23804 |
| **Q8R5M2** | wnt9a | Secreted Factors | Matrisome-associated | 15 | 68 | 15 | -0.01932 | 0.886238 | 0.939949 | 28.71036 | 28.71895 | 28.69963 |
| **Q499E0** | brinp3 | Secreted Factors | Matrisome-associated | 30 | 56 | 26 | 0.019935 | 0.888505 | 0.941335 | 27.15479 | 27.14593 | 27.16587 |
| **Q8C1T8** | clec2h | ECM-affiliated Proteins | Matrisome-associated | 5 | 8 | 5 | 0.064651 | 0.891504 | 0.943384 | 24.44077 | 24.41204 | 24.47669 |
| **Q9R0S2** | mmp24 | ECM Regulators | Matrisome-associated | 13 | 22 | 13 | -0.03735 | 0.892364 | 0.943384 | 26.56316 | 26.57976 | 26.54241 |
| **P59900** | emilin3 | ECM Glycoproteins | Core matrisome | 29 | 72 | 29 | 0.01992 | 0.89577 | 0.945964 | 26.52098 | 26.51213 | 26.53204 |
| **Q61488** | dhh | Secreted Factors | Matrisome-associated | 25 | 59 | 25 | 0.026606 | 0.902762 | 0.952321 | 27.48104 | 27.46921 | 27.49582 |
| **O88992** | c1ql1 | ECM-affiliated Proteins | Matrisome-associated | 11 | 46 | 11 | -0.02359 | 0.905443 | 0.954122 | 27.50853 | 27.51901 | 27.49543 |
| **P63084** | s100a5 | Secreted Factors | Matrisome-associated | 7 | 11 | 7 | -0.11744 | 0.911619 | 0.959599 | 28.27446 | 28.32666 | 28.20922 |
| **P09813** | apoa2 | ECM-affiliated Proteins | Matrisome-associated | 4 | 10 | 4 | 0.052237 | 0.913018 | 0.96004 | 21.70151 | 21.6783 | 21.73053 |
| **P47873** | il11 | Secreted Factors | Matrisome-associated | 13 | 47 | 13 | 0.032859 | 0.915734 | 0.960899 | 26.92516 | 26.91056 | 26.94341 |
| **P11276** | fn1 | ECM Glycoproteins | Core matrisome | 60 | 122 | 60 | -0.01256 | 0.915867 | 0.960899 | 29.12577 | 29.13135 | 29.1188 |
| **Q9ET39** | slamf6 | ECM Glycoproteins | Core matrisome | 9 | 21 | 9 | 0.040261 | 0.916776 | 0.960899 | 24.70436 | 24.68646 | 24.72673 |
| **Q9JL96** | ctsm | ECM Regulators | Matrisome-associated | 13 | 18 | 13 | 0.01839 | 0.921161 | 0.964463 | 26.77284 | 26.76467 | 26.78306 |
| **Q8K0E8** | fgb | ECM Glycoproteins | Core matrisome | 16 | 40 | 16 | -0.01782 | 0.9227 | 0.965044 | 28.39766 | 28.40558 | 28.38776 |
| **P47878** | igfbp3 | ECM Glycoproteins | Core matrisome | 14 | 24 | 14 | -0.02984 | 0.927263 | 0.968129 | 28.37914 | 28.39241 | 28.36256 |
| **P07146** | prss2 | ECM Regulators | Matrisome-associated | 5 | 10 | 5 | 0.027203 | 0.927626 | 0.968129 | 23.95201 | 23.93992 | 23.96713 |
| **P10168** | il7 | Secreted Factors | Matrisome-associated | 3 | 6 | 3 | -0.09323 | 0.936439 | 0.9758 | 23.6238 | 23.66523 | 23.57201 |
| **P70194** | clec4f | ECM-affiliated Proteins | Matrisome-associated | 27 | 39 | 27 | 0.020652 | 0.938302 | 0.9758 | 26.12057 | 26.1114 | 26.13205 |
| **Q9DC11** | plxdc2 | ECM-affiliated Proteins | Matrisome-associated | 14 | 22 | 14 | -0.03587 | 0.938887 | 0.9758 | 24.99214 | 25.00808 | 24.97221 |
| **P41160** | lep | Secreted Factors | Matrisome-associated | 2 | 3 | 2 | 0.026636 | 0.938958 | 0.9758 | 22.06385 | 22.05201 | 22.07865 |
| **O35640** | anxa8 | ECM-affiliated Proteins | Matrisome-associated | 13 | 25 | 13 | -0.01239 | 0.946498 | 0.982593 | 26.19635 | 26.20186 | 26.18947 |
| **Q3U515** | vwce | ECM Glycoproteins | Core matrisome | 21 | 38 | 21 | 0.043752 | 0.948108 | 0.983224 | 25.32518 | 25.30573 | 25.34949 |
| **P57110** | adamts8 | ECM Regulators | Matrisome-associated | 34 | 61 | 34 | 0.022941 | 0.950787 | 0.984026 | 26.26193 | 26.25174 | 26.27468 |
| **O54732** | mmp15 | ECM Regulators | Matrisome-associated | 20 | 43 | 20 | 0.017532 | 0.95089 | 0.984026 | 26.11947 | 26.11168 | 26.12921 |
| **Q9Z0H6** | cst9 | ECM Regulators | Matrisome-associated | 7 | 7 | 7 | -0.07811 | 0.95759 | 0.987813 | 22.02171 | 22.05642 | 21.97832 |
| **P70389** | igfals | ECM Glycoproteins | Core matrisome | 26 | 51 | 26 | -0.0086 | 0.959808 | 0.987813 | 28.41459 | 28.41842 | 28.40981 |
| **P19137** | lama1 | ECM Glycoproteins | Core matrisome | 110 | 192 | 110 | -0.00453 | 0.960386 | 0.987813 | 29.3026 | 29.30461 | 29.30008 |
| **P09586** | prl3b1 | Secreted Factors | Matrisome-associated | 6 | 6 | 6 | 0.011836 | 0.960921 | 0.987813 | 26.04287 | 26.03761 | 26.04945 |
| **P10605** | ctsb | ECM Regulators | Matrisome-associated | 10 | 14 | 10 | -0.00994 | 0.961225 | 0.987813 | 24.61709 | 24.62151 | 24.61157 |
| **Q9JHA8** | vwa7 | ECM Glycoproteins | Core matrisome | 24 | 50 | 24 | -0.01045 | 0.961539 | 0.987813 | 28.00166 | 28.0063 | 27.99586 |
| **Q8CI19** | pdgfc | Secreted Factors | Matrisome-associated | 21 | 72 | 21 | -0.01095 | 0.962021 | 0.987813 | 28.05676 | 28.06163 | 28.05068 |
| **Q9JLV9** | prl2c5 | Secreted Factors | Matrisome-associated | 15 | 30 | 13 | 0.011015 | 0.96488 | 0.987813 | 25.48168 | 25.47679 | 25.4878 |
| **P43407** | sdc2 | ECM-affiliated Proteins | Matrisome-associated | 10 | 18 | 10 | -0.00871 | 0.965018 | 0.987813 | 26.07688 | 26.08075 | 26.07204 |
| **P39876** | timp3 | ECM Regulators | Matrisome-associated | 10 | 18 | 10 | -0.01036 | 0.965568 | 0.987813 | 26.05401 | 26.05861 | 26.04825 |
| **E9PXB6** | sfta2 | ECM-affiliated Proteins | Matrisome-associated | 3 | 3 | 3 | 0.027087 | 0.965979 | 0.987813 | 22.63947 | 22.62743 | 22.65452 |
| **A2ATD1** | ism1 | Secreted Factors | Matrisome-associated | 20 | 26 | 20 | -0.00744 | 0.966645 | 0.987813 | 27.2318 | 27.23511 | 27.22767 |
| **O35235** | tnfsf11 | Secreted Factors | Matrisome-associated | 12 | 16 | 12 | 0.015529 | 0.970611 | 0.988324 | 24.958 | 24.9511 | 24.96663 |
| **P28862** | mmp3 | ECM Regulators | Matrisome-associated | 19 | 34 | 12 | 0.008394 | 0.971422 | 0.988324 | 26.87874 | 26.87501 | 26.88341 |
| **Q07076** | anxa7 | ECM-affiliated Proteins | Matrisome-associated | 8 | 11 | 8 | -0.00841 | 0.972186 | 0.988324 | 27.35437 | 27.35811 | 27.3497 |
| **Q812F3** | hyal5 | ECM Regulators | Matrisome-associated | 16 | 23 | 16 | 0.007022 | 0.972384 | 0.988324 | 27.03396 | 27.03084 | 27.03786 |
| **Q62507** | coch | ECM Glycoproteins | Core matrisome | 21 | 28 | 21 | -0.00758 | 0.972583 | 0.988324 | 26.4192 | 26.42257 | 26.41499 |
| **Q505H4** | vwc2l | Secreted Factors | Matrisome-associated | 6 | 7 | 6 | -0.02545 | 0.974494 | 0.988324 | 23.7555 | 23.76681 | 23.74137 |
| **Q9D777** | tnfsf13 | Secreted Factors | Matrisome-associated | 5 | 14 | 5 | -0.01417 | 0.97479 | 0.988324 | 27.73813 | 27.74443 | 27.73026 |
| **P63075** | fgf17 | Secreted Factors | Matrisome-associated | 11 | 16 | 10 | -0.00578 | 0.975213 | 0.988324 | 26.1133 | 26.11587 | 26.11009 |
| **P27467** | wnt3a | Secreted Factors | Matrisome-associated | 14 | 15 | 13 | 0.011116 | 0.977561 | 0.989679 | 25.4341 | 25.42916 | 25.44028 |
| **A2ASQ1** | agrn | ECM Glycoproteins | Core matrisome | 65 | 155 | 65 | 0.003532 | 0.981952 | 0.993099 | 29.78212 | 29.78055 | 29.78408 |
| **Q9DCT8** | crip2 | ECM-affiliated Proteins | Matrisome-associated | 8 | 10 | 8 | 0.020618 | 0.985124 | 0.994629 | 20.96342 | 20.95425 | 20.97487 |
| **P97430** | slpi | ECM Regulators | Matrisome-associated | 5 | 5 | 5 | 0.007677 | 0.985606 | 0.994629 | 26.07685 | 26.07344 | 26.08111 |
| **Q80T21** | adamtsl4 | ECM Regulators | Matrisome-associated | 41 | 89 | 41 | -0.00259 | 0.986509 | 0.994629 | 28.00888 | 28.01003 | 28.00744 |
| **Q5I2A0** | serpina3g | ECM Regulators | Matrisome-associated | 15 | 29 | 11 | -0.00615 | 0.989773 | 0.996087 | 23.77428 | 23.77702 | 23.77086 |
| **Q6P4P1** | serpina3a | ECM Regulators | Matrisome-associated | 12 | 20 | 10 | -0.00325 | 0.990677 | 0.996087 | 27.53718 | 27.53862 | 27.53537 |
| **Q9JIA9** | ctsr | ECM Regulators | Matrisome-associated | 6 | 9 | 6 | 0.007822 | 0.991779 | 0.996087 | 26.2451 | 26.24162 | 26.24944 |
| **Q4ZJM9** | c1ql4 | ECM-affiliated Proteins | Matrisome-associated | 19 | 44 | 18 | -0.00163 | 0.992707 | 0.996087 | 28.41537 | 28.4161 | 28.41447 |
| **Q8VCD3** | lman1l | ECM-affiliated Proteins | Matrisome-associated | 16 | 32 | 16 | 0.001656 | 0.993363 | 0.996087 | 26.95266 | 26.95192 | 26.95358 |
| **Q9D2H8** | fndc8 | ECM Glycoproteins | Core matrisome | 12 | 19 | 12 | 0.001554 | 0.994054 | 0.996087 | 26.5808 | 26.58011 | 26.58166 |
| **Q7TSL0** | ifnk | Secreted Factors | Matrisome-associated | 10 | 15 | 10 | -0.00015 | 0.999306 | 0.999808 | 25.43102 | 25.43109 | 25.43093 |
| **Q7TMF5** | serpina12 | ECM Regulators | Matrisome-associated | 20 | 31 | 20 | 3.51E-05 | 0.999808 | 0.999808 | 27.07849 | 27.07847 | 27.0785 |

Supplementary table 2. Number of total unique peptides, per category, found in each sample group and number of peptides (and as percentage in brackets) with at least one identified N-glycosylation. Peptides were counted as present if found in at least two out of eight samples in the young respectively old group. For details on protein level see peptide report available in University of Surrey Open research repository.

|  | **Young samples**  *16416 peptides* | | **Old samples**  *15574 peptides* | |
| --- | --- | --- | --- | --- |
| **Category** | **Number of unique peptides** | **Number of unique**  **N-glycosylated peptides** | **Number of unique peptides** | **Number of unique**  **N-glycosylated peptides** |
| **Collagens** | 3611 | 667 (18.5%) | 3423 | 634 (18.5%) |
| **ECM-affiliated Proteins** | 2929 | 1110 (37.9%) | 2743 | 1048 (38.2%) |
| **ECM Glycoproteins** | 3615 | 1375 (38.0%) | 3448 | 1306 (37.9%) |
| **ECM Regulators** | 2973 | 1165 (39.2%) | 2828 | 1079 (38.2%) |
| **Proteoglycans** | 627 | 260 (41.5%) | 605 | 249 (41.2%) |
| **Secreted Factors** | 2661 | 884 (33.2%) | 2527 | 828 (32.8%) |
